# Latent Dirichlet Allocation reveals tomato root-associated bacterial interactions responding to hairy root disease

**DOI:** 10.1186/s40793-025-00822-2

**Published:** 2025-11-23

**Authors:** Peiyang Huo, Pablo Vargas Ribera, Hans Rediers, Jan Aerts

**Affiliations:** 1 Augmented Intelligence for Data Analytics (AIDA) Lab, Biosystems, KU Leuven, Leuven, Belgium; 2 Microbial and Plant Genetics, Microbial and Molecular Systems, KU Leuven, Leuven, Belgium; 3 Leuven Plant Institute, KU Leuven, Leuven, Belgium; 4 Leuven.AI Institute for Artificial Intelligence, KU Leuven, Leuven, Belgium; 5 Sustainable Plant Protection, Institute of Agrifood Research and Technology (IRTA Cabrils), Barcelona, Spain

**Keywords:** Hairy root disease, 16S rRNA amplicon sequencing, Hydroponic greenhouse, Microbiota, Agrobacterium, Visual analytics, Latent Dirichlet Allocation

## Abstract

**Background:**

Hairy root disease (HRD), caused by rhizogenic *Agrobacterium* strains, is a significant disease threat to modern hydroponic greenhouses, which can result in up to 15% loss in yield. Our prior research has suggested increased alpha diversity after infection in hydroponic tomato root-associated microbiota. However, a more detailed investigation of how root-associated microbial components (MCs; clusters of weighted bacterial features) respond to disease and the underlying mechanisms remains lacking. To address this gap, we applied Latent Dirichlet Allocation (LDA) to analyze MCs from 12 Belgian commercial hydroponic tomato greenhouses. Using high-throughput amplicon sequencing of the 16S rRNA locus, three locations along each greenhouse irrigation system (beginning, middle, and end) were sampled at 5 time points throughout the 2018 growing season.

**Results:**

In this study, we used LDA to identify root-associated MCs and gained insights into temporal changes and new health statuses. First, we observed a structured temporal pattern from the early stage (ES; sampling time points 1 and 2) through a transitional stage (TS; sampling time point 3) to the late stage (LS; sampling time points 4 and 5), showing different MC trajectories by health status. Second, MC4 (characterised by *Paenibacillus* spp.) was pronounced for healthy greenhouses in the ES, MC7 (characterised by rhizogenic *Agrobacterium* spp., *Devosia* and *Limnobacter* amplicon sequence variants (ASVs)) was pronounced for pre-symptomatic status, while MC0 (characterized by *Comamonadaceae* spp. ASVs) was indicative of an intermediate state between healthy and infected conditions. Furthermore, the ratio between *Paenibacillus* ASV and rhizogenic *Agrobacterium* ASV can be used as a biomarker to assess greenhouse health status in both ES and LS.

**Conclusion:**

We investigated hydroponic tomato root-associated MCs responses to HRD using LDA, which revealed different MC trajectories in terms of plant health. Our study advances knowledge of hairy root disease regarding the mechanisms that can improve plant health monitoring in greenhouses and biocontrol strategies. From a computational perspective, we demonstrate how to apply LDA-a powerful analytical tool-to understudied subfields through visual analytics.

## Background

Hydroponics has been widely adopted in modern commercial greenhouses, which considerably increases crop yields. It provides optimal nutrient delivery, water efficiency, and controlled growing conditions that maximize plant growth and productivity. However, despite the controlled environment of hydroponic systems, plant pathogens remain a critical challenge. The recycled nutrient solution and lack of natural soil barriers can facilitate rapid disease spread throughout these systems. For example, hairy root disease (HRD) is currently a major threat to hydroponics cultivation of tomato, cucumber, eggplant, and bell pepper [1–3].

HRD is caused by rhizogenic *Agrobacterium* strains harboring the Ri-plasmid (root-inducing plasmid), and has been reported worldwide in various hydroponically grown crops over the last decades [2–4]. The most remarkable symptom is excessive root formation, which provokes a hormonal and physiological imbalance in the root-to-shoot ratio, ultimately resulting in yield losses of up to 10–15% [2, 5].

In earlier work, we investigated the root-associated microbiota in hydroponic tomato greenhouses and showed that HRD infection increases the diversity of the bacterial community [3]. Other studies demonstrated that the biocontrol organism (BCO) *Paenibacillus* *xylanexedens* considerably reduces HRD infections in greenhouses [2, 6, 7]. However, the analysis of microbiota data from greenhouse studies faces several challenges when exploring plant-microbiota interactions. These challenges include not only inherent data compositionality and sparsity, but also inter-sample heterogeneity and complex factors [8–10]. Although the causative agent of HRD has been identified, we hypothesized that substantial untapped information likely remains hidden in the microbiota profiles [11].

To address this, we applied Latent Dirichlet Allocation (LDA), an unsupervised probabilistic modeling method gaining interest in biology [12, 13]. It is widely used in natural language processing (NLP) to find topics in collections of documents, a process known as topic modeling. In the microbiota context, LDA functions as both a dimensionality reduction and clustering method, which reduces complex dataset into a smaller number of interpretable microbial components (MCs), and allows MCs to co-exist within a single sample [14]. MCs are modeled from microbial features’ co-occurring relationships; some microbial features could be shared between MCs, but each MC comprises a characteristic distribution. In microbiome research, LDA has been successfully applied mainly in clinical settings to distinguish health status [14–16], microbial niches [17, 18], enterotypes [19–21], and spatial and temporal patterns [14, 15, 22, 23].

Our previous research encountered heterogeneity that was attributed to overlapping bacterial communities within the dataset. Such complexity reduces the effectiveness of principal coordinates analysis (PCoA). We hypothesized that detailed MC analysis of the existing data could yield additional insights into the dynamics of the root-associated bacterial communities. More specifically, the following research questions were addressed: (i) can we provide deeper insights into the temporal dynamics of microbial communities before, during, and after the onset of HRD symptoms; (ii) can we identify core microbiota that may act as key drivers of plant health or disease, and that have high predictive value even before visible disease symptoms occur. In addition to these insights, the LDA results also led us to generate meaningful hypotheses.

## Methods

### Samples collected from hydroponic tomato greenhouses

**Fig. 1:**
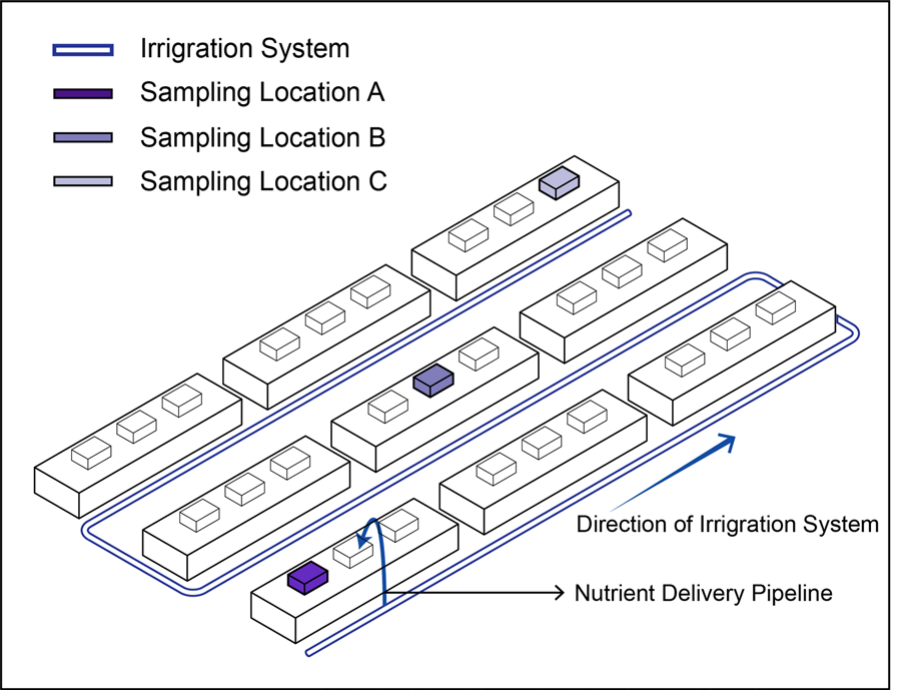
Diagram of the irrigation system in a typical tomato hydroponic greenhouse. Sampling locations are shown at the start (**A**), middle (**B**), and end (**C**) of the irrigation system

The root-associated microbiota data used in this analysis were collected from 12 commercial hydroponic tomato greenhouses using rockwool as the growing medium in Flanders, Belgium [7]. A considerable variation in cultivation techniques was observed across these greenhouses, including differences in rootstock-scion combinations and water treatment methods (see Table 1 in Additional file 1). Sampling was conducted throughout the 2018 growing season (going from January to October) and was divided into 5 sampling times. At each sampling time, samples were collected from three locations within each greenhouse, more specifically at the start, middle, and end of the irrigation system (Fig. 1). At each location, two composite samples were collected as technical replicates. For all samples, the bacterial V4 region of the 16S rRNA gene was amplified using sample-specific barcode-labelled versions of primer set 515F-806R following Kozich et al. [24].

HRD incidence was assessed through visual inspection of plant roots and confirmed using quantitative PCR (qPCR) specifically targeting rhizogenic *Agrobacterium* DNA. Detailed methods for biosample collection and processing, 16S rRNA gene amplicon sequencing, and qPCR targeting DNA from rhizogenic agrobacteria were previously described [7]. It is worthwhile mentioning that the data retrieved included? a mock community and negative DNA extraction and PCR controls. Ct values from qPCRs were available for all sampling time points, but grouped by greenhouse. Greenhouses 1, 4, 5, and 7 were diagnosed with HRD (greenhouse 5 at sampling time point 4, others at sampling time point 2). Additionally, greenhouses 2 and 12 showed limited HRD infection only at sampling time 5 by the end of the growing season. Greenhouses 3, 5 and 6 were infected in the previous year (2017). In greenhouses 8-11, no HRD symptoms were detected throughout the season, and were assigned “healthy”.

### Processing of amplicon sequence reads

Sequencing data were processed with the DADA2 (version 1.10.0) plugin for denoising within the QIIME2 (version 2020.2) platform and using default parameters [25, 26], resulting in amplicon sequence variants (ASVs). The resultant ASV table was processed in QIIME2 (version 2024.2) according to the Amplicon SOP v2 from Microbiome Helper [27].

After DADA2 processing, 336 samples were retained. Using the ’feature-table filter-features’ command in QIIME2, 653 rare ASVs were removed with frequencies below 20 (i.e. 0.1% of the average sequencing depth of 20,156) across all samples. These ASVs were filtered out to prevent rare sequencing errors from being considered real ASVs and to avoid artefacts from MiSeq bleed-through between runs [27]. Taxonomic classification was performed using a Naive Bayes classifier that was pre-trained on the SILVA database (release 138.1) [28, 29]. A total of 108 contaminant and unclassified ASVs were subsequently removed, including those classified as *Mitochondria*, *Chloroplast*, and ASVs without Phylum-level taxonomic assignments. A minimum sequencing depth threshold of 7,000 reads for downstream analyses was established; the majority of rarefaction curves demonstrate a notable inflection point at this depth (Fig. 1 in Additional file 1). After quality assessment, 47 samples with sequencing depth below 7,000 reads were excluded from further analysis. The feature table after these processes was comprised of 289 samples and 6,866 ASVs, with technical replicates maintained without consolidation. Shannon diversity indices were computed using the ’Core metrics’ command for subsequent analyses.

**Fig. 2:**
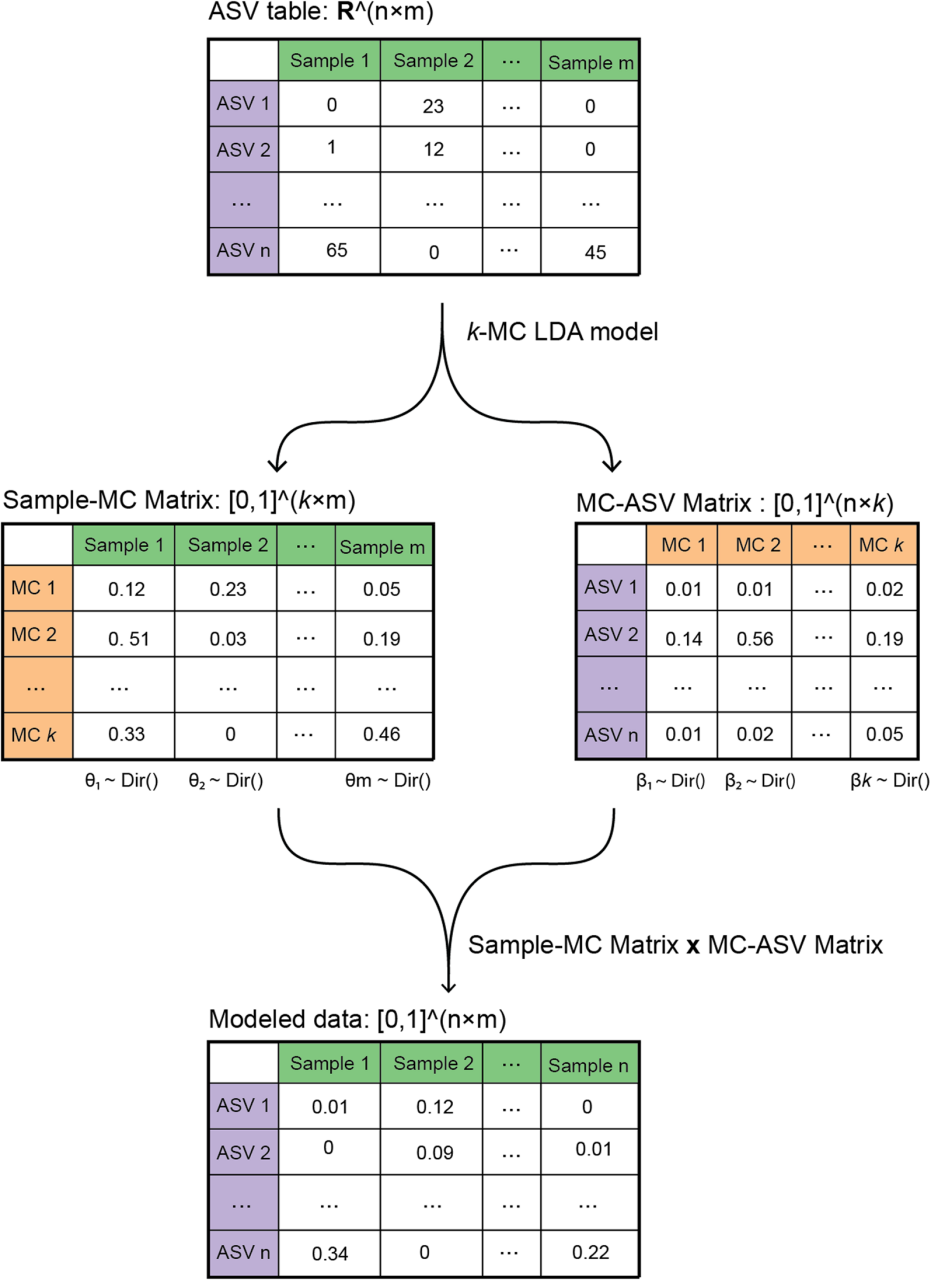
Application of Latent Dirichlet Allocation (LDA) to microbiota data. The input ASV table ($\mathbb {R}^{n \times m}$) containing raw counts of *n* Amplicon Sequence Variants (ASVs) across *m* samples is decomposed by *k*-MC LDA into two probability matrices: (1) Sample-MC Matrix $\in [0,1]^{k \times m}$ represents the distribution of *k* Microbial Components (MCs) in each sample; and (2) MC-ASV Matrix $\in [0,1]^{n \times k}$ represents the composition of ASVs within each MC. The product of Sample-MC matrix and MC-ASV matrix generates a modeled data matrix $\in [0,1]^{n \times m}$

Regarding taxonomic assignment, it is worth mentioning that ASVs identified as *Allorhizobium, Neorhizobium, Pararhizobium, and Rhizobium* were grouped as“Rhizobium complex”. Further, ASVs corresponding to Rhizobium complex 25 were subjected to a NCBI BLAST (version 2.10.1) search against an in-house dataset of 21 16S rRNA gene sequences of rhizogenic *Agrobacterium* bv. 1 strains (GenBank accessions MZ298106-MZ298126) (Fig. 2 in Additional file 1) [30, 31].

### Analysis

We employed visual analytics as the underlying research approach. Visual analytics deeply integrates data analysis and machine learning methods with interactive data visualization. Python programming language version 3.9.21 was used in this study, with the exception of Linear discriminant analysis Effect Size (LEfSe), which was implemented in R (version 4.0.1) [32]. LDA was applied with an exploratory purpose; interactive visualization was particularly valuable in addressing interpretation challenges inherent to LDA (see Sect. 2.3.1). Following LDA modeling, we conducted correlation analysis and biomarker identification by LEfSe. Newly identified patterns were subsequently validated through confirmatory testing (see Sect. 2.3.2).

#### Latent Dirichlet Allocation (LDA)

LDA is a hierarchical Bayesian probabilistic modeling technique widely used in machine learning [33]. Originally developed for natural language processing (NLP), LDA reveals latent topics in document collections by assuming each document contains multiple semantic topics. In this study, we adapt LDA to microbiota data, where corpus (collection of documents), document, topic, and word from the NLP context correspond to environment, biological sample, microbial component, and ASV, respectively. In terms of mapping ’topic’ to ’microbial component’, other research either retains ’topic’ [18] or adopts alternative terms such as ’community’ [13], ’sub-communities’ [17], ’subgroup’ [21], or ’assemblages’ [20]. Our terminology approach aims to enhance interpretability, conceptually paralleling principal components (PCs) in principal component analysis (PCA) while carrying more biological meaning than PCs (see Sect. 3.2).

LDA functions as both a dimensionality reduction similar to PCA and as a clustering method in which each MC represents a cluster of weighted ASVs. Unlike hard clustering methods that strictly assign ASVs to a certain cluster, LDA allows MCs to share ASVs but with different probabilities. This soft clustering approach enables MCs to maintain distinct overall compositions while still reflecting the inherent overlap in ASVs. Previous applications of LDA in microbiome research have agreed that LDA’s assumptions align with the microbiota context [13, 14, 17, 20, 21], where each sample is typically represented by multiple MCs, with individual ASVs potentially associated with multiple MCs at varying probabilities.

LDA offers two key advantages for microbiome analysis. First, it uses Dirichlet distributions as priors, which naturally handle compositional data by generating positive components that sum to 1. This mathematical alignment has driven LDA’s adoption in numerous microbiome tools including SourceTracker [34], DMM [19], ALDEx2 [35], and SparCC [36]. Second, LDA uses Bayesian inference via Gibbs sampling to infer latent MCs based on ASV co-occurrences, enabling robust handling of sparse data and sampling zeros [22, 37].

LDA produces one Sample-MC probabilistic matrix and one MC-ASV probabilistic matrix (Fig. 2). Samples are represented by MCs with varying probabilities (e.g., sample A: 0.70$\times$MC0, 0.25$\times$MC1, 0.05$\times$MC2; sample B: 0.30$\times$MC0, 0.10$\times$MC1, 0.40$\times$MC2). Higher probabilities indicate stronger MC characteristics in a sample. Similarly, MCs are composed of ASVs with varying probabilities (e.g., MC0: 0.50$\times$ASV1, 0.30$\times$ASV2, 0.20$\times$ASV3; MC1: 0.10$\times$ASV1, 0.10$\times$ASV2, 0.80$\times$ASV3). Higher ASV probabilities within an MC indicate that the ASV is more representative of that MC. Shafiei et al. have used this framework to model relative abundance by combining the Sample-MC matrix and the MC-ASV matrix [14]. This aligns with the Law of Large Numbers, where theoretical probability distributions converge to observed relative abundances as sample size increases. We have also adopted this extension in our study.

For implementation, we used the *MALLET* toolkit (version 202108) through the *little-mallet-wrapper* package (version 0.5.0) in Python, which trains LDA by Gibbs sampling [37, 38]. It was trained using the *train-topics* command with 1000 iterations to ensure convergence.

LDA requires a predefined parameter *k* that specifies the number of MCs to extract (Fig. 2). This parameter was selected from a smaller range (2-20; compare with NLP applications) to facilitate biological interpretation. This strategy aligns with approaches used in other microbiota applications [13, 16, 17, 20, 21, 23]. First, each model’s perplexity and coherence scores were calculated [33, 39]; these two metrics are widely used for evaluation in NLP, where lower perplexity indicates better statistical prediction of data and higher coherence scores suggest improved human interpretability. Second, all 209 MCs generated across all models were stored to identify potential MC hierarchy. These MCs were treated as vectors in high-dimensional space and were clustered using HDBSCAN (Hierarchical Density-Based Spatial Clustering of Applications with Noise; using the Python package *hdbscan*, version 0.8.39), enhanced by UMAP (Uniform Manifold Approximation and Projection, using the Python package *umap-learn* version 0.5.7) [40]. Because MC hierarchy existed as *k* increased, one MC might split into two or more. Therefore, similar MCs across different models were clustered, and then the number of clusters was used to further narrow down the selection range. The final *k* value was selected based on three criteria: maximizing the number of MC clusters while achieving low perplexity and high coherence scores.

**Fig. 3:**
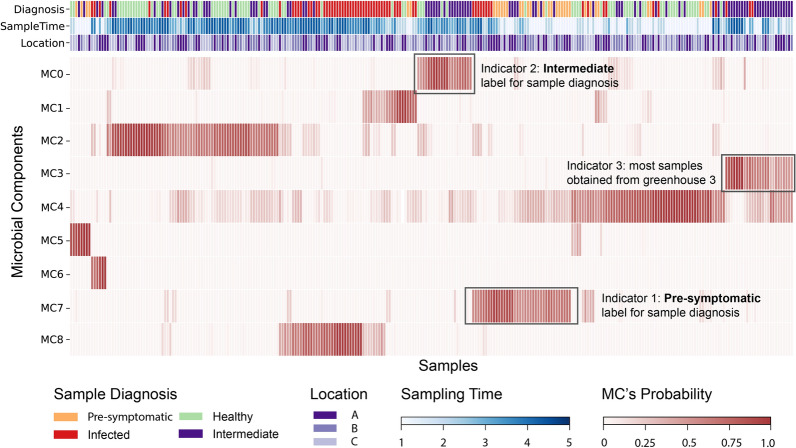
Clustered Sample-MC matrix heatmap annotated with sample metadata. Each column is one sample with different MCs' probabilities and metadata. Samples were hierarchically clustered using the *clustermap* function from the *Seaborn* package (version 0.13.2) in Python. Black boxes illustrate our exploratory visual analysis, which contributes to the new labels. Indicator 1: Samples clustered based on MC 7 were initially labeled as healthy. An interactive analysis revealed that a large proportion of these samples were actually collected from infected greenhouses in the time point just before disease symptoms were detected (“pre-infection”). Indicator 2: Samples clustered based on MC 0 were initially labeled as healthy. Interactive visual analytics revealed that these greenhouses either exhibited limited infection at the end of the growing season or had experienced infection in the previous year (i.e., “intermediate” healthy and diseased status). Indicator 3: Most samples clustered based on MC 3, and most of them were obtained from greenhouse 3. Heatmaps with different ordering strategies and interface URL are available in Additional file 3

Once the optimal *k* value was determined, two matrices were obtained and a modeled relative abundance table was derived from multiplying these two matrices (Fig. 2). We visualized the Sample-MC matrix as a heatmap and annotated it with sample metadata (Fig. 3). Annotations facilitate insight generation when the matrix reveals patterns.

For the MC-ASV matrix, ASVs were annotated at the genus level or, when not possible, at the highest possible taxonomic level, with unique IDs assigned to distinguish ASVs within the same taxonomic group (unique IDs starting from 0). For interpretation purposes, we used a subset of the MC-ASV matrix, taking the top 8 ASVs from each MC with a minimum probability of 0.01 . This selection criterion was implemented because the resulting matrix, when combined with the Sample-MC matrix, produced a modeled table with a similar explanatory proportion as that based on the top 10 bacterial genera (see Fig. 3 in [7]). For the visualization, a clustered heatmap was used, with clustering performed using the *hierarchy* function from the *scipy* package (version 1.7.3).

These visualizations were enhanced through a custom interactive interface for exploratory analysis. The interface enables dynamic ordering strategies and allows users to hover over data points within newly identified patterns to examine their associated metadata for enhanced interpretation (the interface URL is available in Additional file 3).

#### Additional exploratory and confirmatory analysis

For exploratory purposes, we also conducted correlation analyses between MCs and qPCR results separately. For confirmation, we tested the observed patterns from LDA using various statistical approaches tailored to specific analytical goals. Permutational Multivariate Analysis of Variance (PERMANOVA) and Kruskal–Wallis tests were applied for overall differences among the multiple groups, followed by a pairwise tests. All p-values were adjusted for multiple comparisons using false discovery rate (FDR) methods. LEfSe was conducted both for validating patterns obtained from LDA and improving LDA interpretability.

**Pearson correlation analysis** was performed to examine relationships between Microbial Components (MCs) derived from LDA and qPCR cycle threshold (Ct) values. Correlations and corresponding *p*-values were calculated using the *pearsonr* function from the *scipy* package (version 1.7.3). *P*-values were adjusted using the Benjamini-Hochberg procedure (BHP) via the *multipletests* function from the *statsmodels* package (version 0.14.4). Correlations with adjusted *p*-values $< 0.05$ were considered statistically significant.

**Fig. 4:**
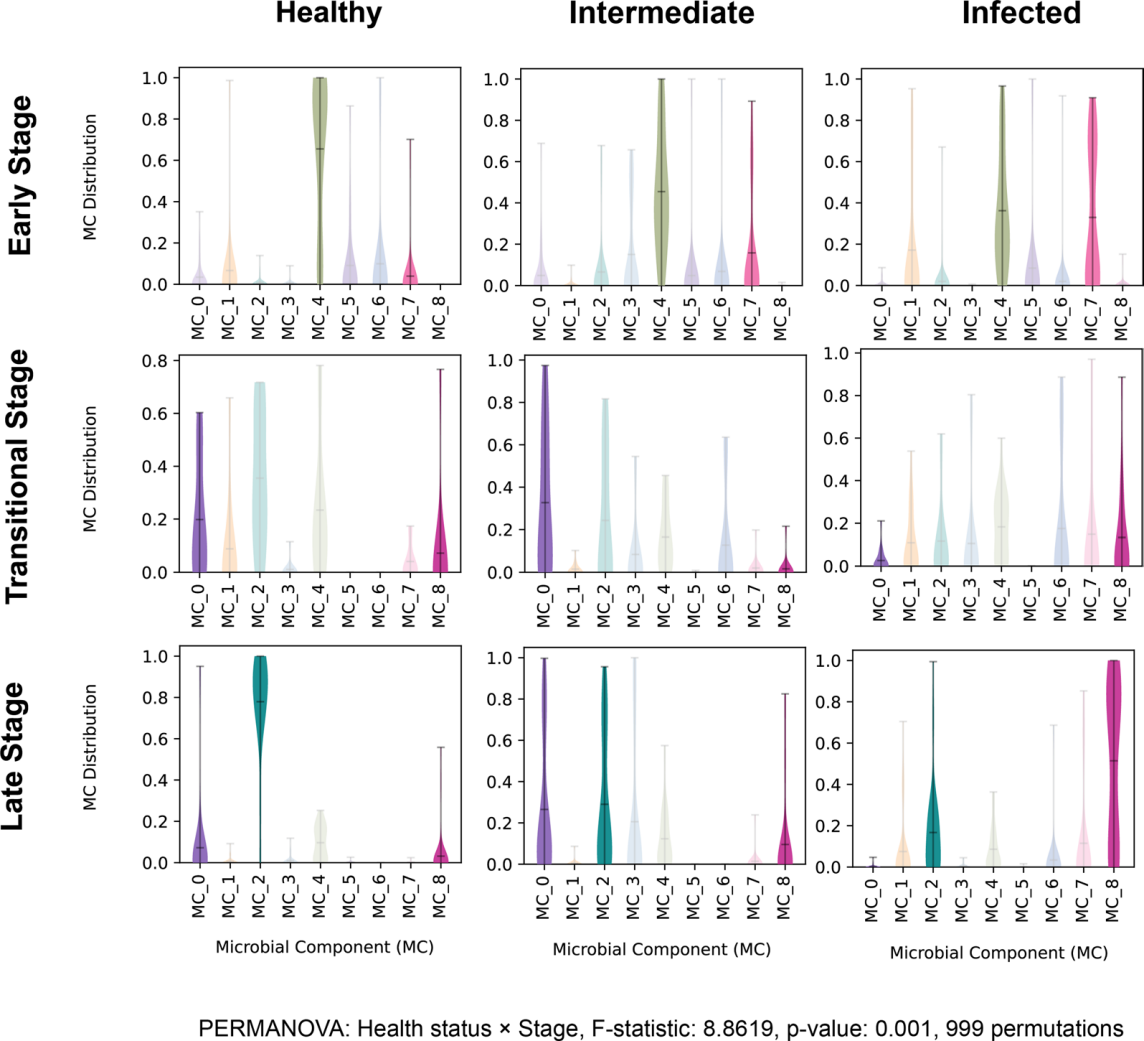
MC probability distributions across different stages throughout the season, i.e., early stage (corresponding to time point 1 and 2), a transitional stage (corresponding to time point 3), and a late stage (corresponding to time point 4 and 5). MC4 and MC7 (ES), MC0 and MC8 (TS), and MC0, MC2 and MC8 (LS) are emphasized by fading other MCs. Emphasized distributions indicate both visual and statistical significance (statistical results are available in Table 2 in Additional file 2). PERMANOVA analysis confirmed significant differences among the newly established categories (3 temporal stages and 3 types of greenhouses) (F-statistic: 8.8619, *p*-value: 0.001, 999 permutations)

**Categorical Comparisons.** PERMANOVA (with *permutations*=999) was conducted using the *permanova* function from *skbio* (version 0.6.3) in Python to test the statistical significance of the newly established greenhouse and temporal categories derived from LDA (Fig. 4). To analyze MC distributions across these new categories, Kruskal–Wallis tests were employed using the *stats.kruskal* function in the *scipy* package (version 1.7.3) in Python. These tests were followed by Dunn’s post-hoc tests with Bonferroni correction for pairwise comparisons using the *posthoc_dunn* function from *scikit-posthocs* package (version 0.11.4) in Python. The Bonferroni method was used here due to limited sample sizes.

**Fig. 5:**
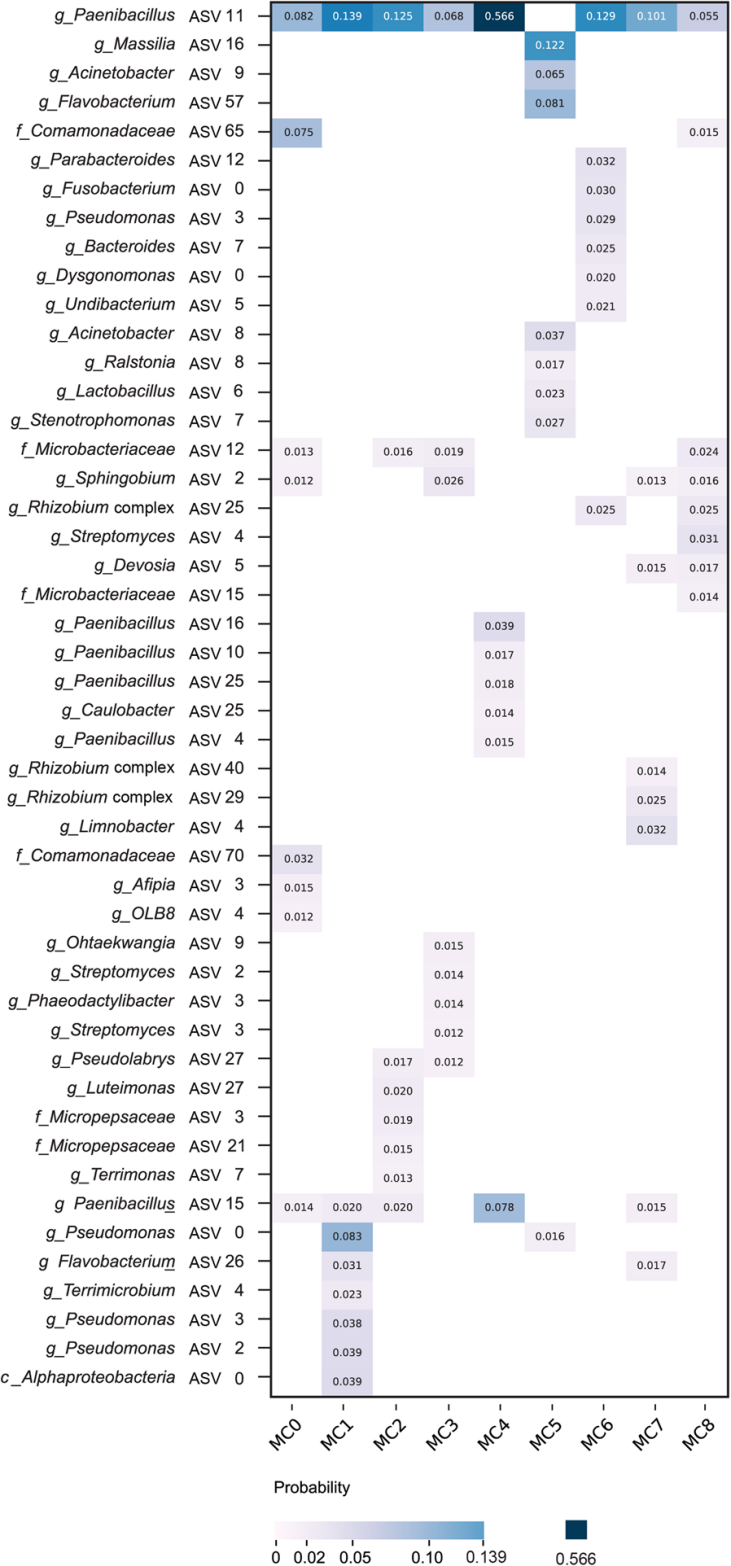
Heatmap visualization of the MC-ASV probability matrix, displaying the top 8 ASVs per microbial component with minimum probability of 0.01. For clustering of heatmap see Sect. 2.3.1. The selection criteria for the top 8 ASVs (minimum probability >0.01) were established because these ASVs collectively explained an average of 40% of the modeled ASV table (range: 18.1%$-$74.7%, median: 35.8%), which provides a similar explanatory proportion to that based on the top 10 bacterial genera (see Fig. 3 in [7]). A comparison between observed and modeled relative abundances in the ES and LS for the top 10 bacterial genera is provided in Fig. 5 of Additional file 1

**Fig. 6:**
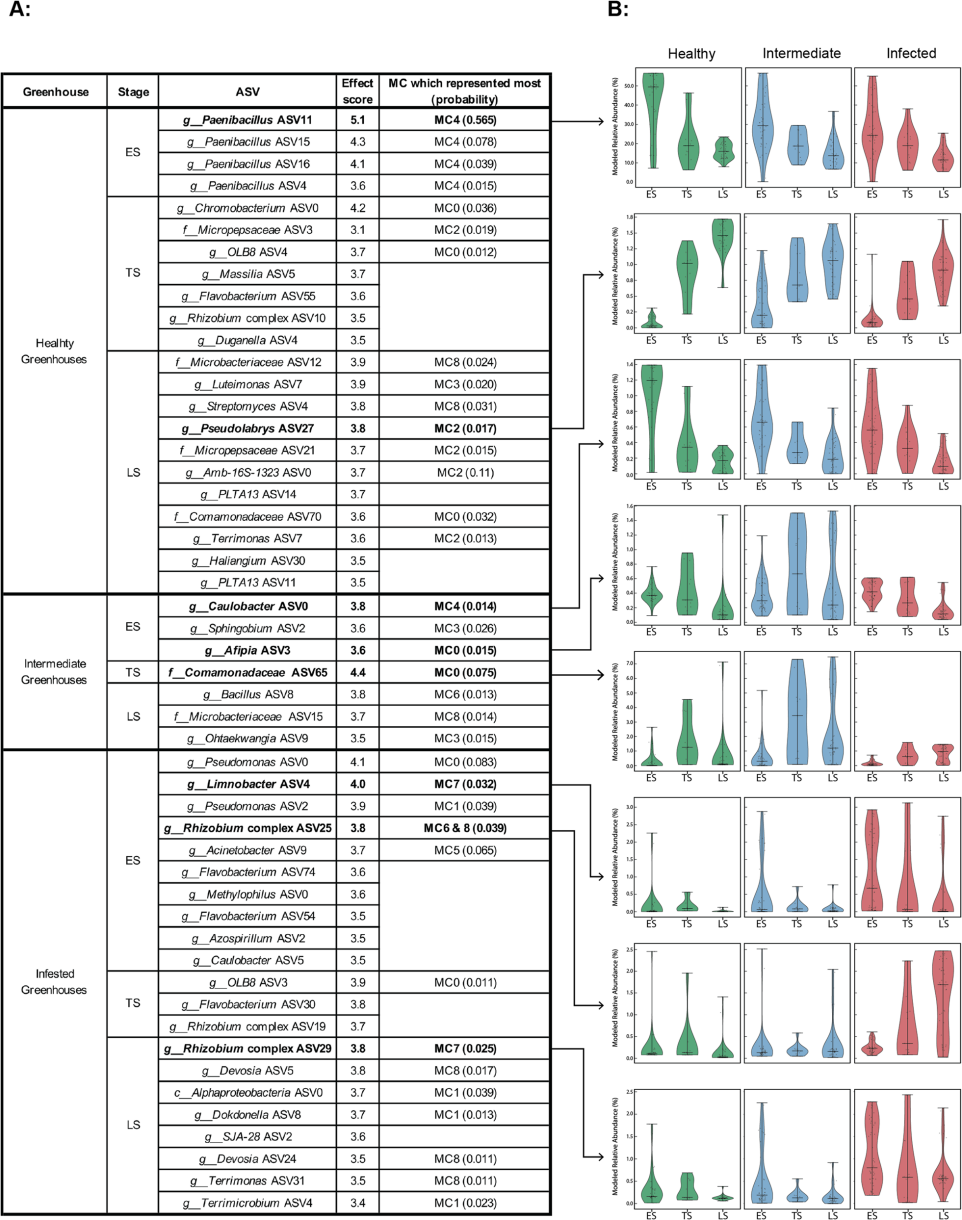
Tracking biomarkers across different types of greenhouses. **A:** LEfSe analysis results at the ASV level based on the newly established categories, with an effect size threshold of 3.5. Results include the most characteristic MC assignment and associated probability. Biomarkers of interest that are highlighted with bold font are plotted in Panel (**B**). **B:** Violin plots show the distribution of selected biomarkers across the newly established categories, based on modeled relative abundance data

**Fig. 7:**
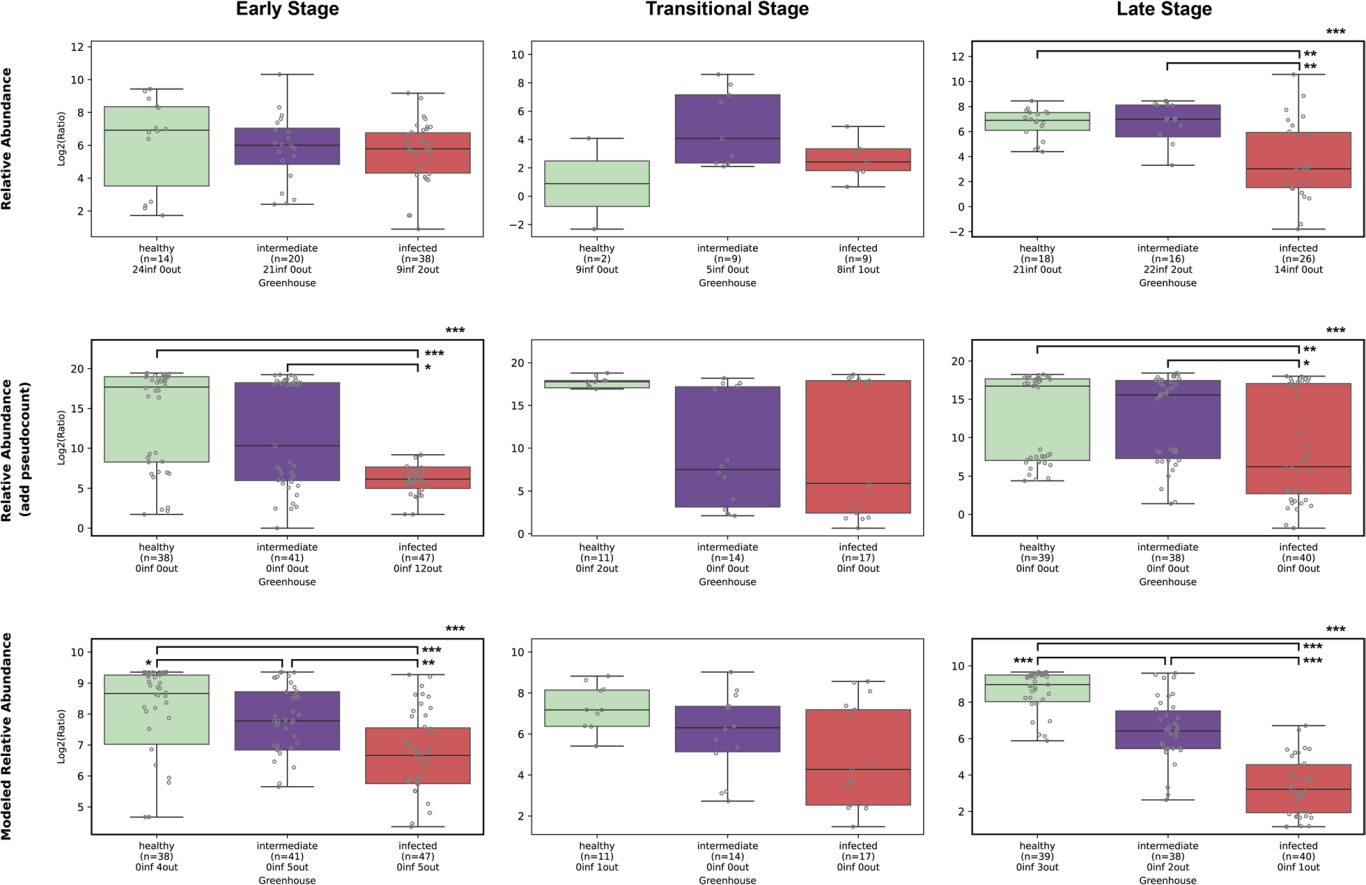
Abundance ratios (log2-transformed) of *Paenibacillus* ASV11 to *Rhizobium* complex ASV25 across greenhouse categories. Box plots show the distribution of abundance ratios (log2-transformed) calculated using Eq. (1) for observed relative abundance (top row), relative abundance with pseudocount (middle row) and modeled relative abundance (bottom row) data across three stages. Sample sizes, the number of infinity values (caused by absence of *Rhizobium* complex ASV25 in observed table), extreme points (out) are not plotted but shown under X-axis labels. Significant differences are indicated by asterisk annotations. Asterisks indicate statistical significance levels: *p < 0.05, **p < 0.01, ***p < 0.001

**Biomarker Identification.** LEfSe was conducted to find biomarker candidates from the newly established categories and as a validation for patterns found by LDA [32]. To this end, annotated taxonomy, ASV table, and metadata were used to make a *MicroEco* object [41] (version 1.15.0), which was used as input for the *trans_diff* function, specifying *lefse* as method, *ASV* as taxa level, and *fdr* as *p*-value adjustment method. Given LEfSe’s rank-based approach, we selected results with sufficient relative abundance and characteristic association with specific MC (Fig. 5), and then visualized them for biological interpretation (Fig. 6A). The selected biomarkers were tracked across all samples and plotted as a violin plot (Fig. 6B). Two biomarkers were selected for further BCO-pathogen ratio analysis within the newly defined category. These ratios were visualized using box plots across three types of data: observed relative abundance, relative abundance with a pseudocount added, and modeled relative abundance estimated by LDA (Fig. 7). A small pseudocount (0.000001) was added to both the numerator and denominator to prevent infinite ratio values caused by zeros, particularly in cases where Rhizobium complex 25 was not detected. Its absence could reflect either a true biological absence or a limitation in detection. Missing values in the denominator can result in infinite ratios, which are filtered out during statistical testing and may introduce bias. Kruskal–Wallis tests were applied for overall differences among different groups and followed by Mann–Whitney U tests and corrected by BHP. Adjusted *p*-values < 0.05 were considered statistically significant.

## Results

### Sample—MC matrix

First, LDA was used to define and assign MCs to all samples. Based on the methods described in Sect. 2.3.1 (see also Fig. 3 in Additional file 1), k=9 was selected in the LDA model. Second, the Sample-MC matrix is visualized as a heatmap annotated with sample metadata (Fig. 3; see heatmaps by different order strategies in Additional file 3). MCs revealed distinctive patterns within specific sample groups. Overall, the Sample-MC matrix revealed two main insights: (i) A structured temporal progression was observed, including early stage (ES; sampling time point 1 and 2), transitional stage (TS; sampling time point 3) and late stage (LS; sampling time point 4 and 5), with greenhouses of each health status exhibiting unique variations within this temporal pattern (Sect. 3.1.1). (ii) MC4 was pronounced for healthy greenhouses in the ES (Sect. 3.1.2); MC7 was distinctly pronounced in pre-symptomatic (this label will be introduced in Sect. 3.1.2) samples; MC0 pronounced in the LS, and 4 greenhouses previously classified as healthy were reclassified as having an“intermediate”status (see Sect. 3.1.2). Additionally, MC3 appeared predominantly in greenhouse 3, while MC5 and MC6 exhibited limited distribution patterns across all samples.

#### Temporal pattern regards to microbial component

The MC distribution revealed sample temporal patterns in two different health statuses (healthy or infected). The samples primarily clustered into ES and LS, with sampling time point 3 serving as a TS.

Within this temporal framework, healthy and infected greenhouses showed exclusive MC transitions (Fig. 3; see the heatmap ordered by sample time point in Fig. 3 in Additional file 3). In healthy greenhouses, there seems to be a shift from MC4 in the ES towards MC2 in LS. MC4 is dominated by *Paenibacillus* spp. ASVs, with *Paenibacillus* ASV11 probability up to 0.566 (Fig. 5). MC2 was also characterized by *Paenibacillus* spp. ASVs that maintained high probability but were less dominant (*Paenibacillus* ASV11 probability decreased to 0.125), along with *Micropepsaceae* spp. ASVs. Conversely, in infected greenhouses, a shift was observed from MC7 in the ES to MC8 in the LS. MC7 was characterized by comparably low probability of *Paenibacillus* ASV11 in the ES, while showing high probabilities of *Limnobacter* ASV4, *Rhizobium* complex spp., and *Devosia* ASV5. Interestingly, MC8 was characterized by high probabilities of *Rhizobium* complex ASV25 that matches the in-house pathogen. Among infected greenhouses, different temporal patterns emerged. Greenhouse 1 (with the highest infection rate: 82%) showed higher MC1 probability (characterized by *Pseudomonas* spp. ASVs) in the ES, then transitioned to MC8 in the LS. Greenhouse 4 (with the lowest infection rate: 11%) showed higher MC7 probability in the ES before shifting to MC1 in the LS.

#### Health status beyond diagnosis

MC distribution also revealed two additional and statistically significant subgroups that were not identified before, which could be labelled as: ’pre-symptomatic’ for sampling time point and ’intermediate’ for greenhouse (PERMANOVA F-statistic=8.8619: *p*-value=0.001; 999 permutations). The first category label ’pre-symptomatic’ was assigned to samples collected at the time point just prior to observation of the first disease symptoms in infected greenhouses, and was mainly characterized by MC7 (Fig. 3; see the heatmap ordered by diagnosis in Fig. 1 in Additional file 3). This suggests potential early MC signatures preceding visible HRD symptoms and pathogen detection. The second label, ’intermediate’ greenhouse, was assigned to samples from greenhouses 2, 3, 6, and 12, based on MC0 (characterized by *Comamonadaceae* spp., *Sphingobium* ASV2 and *Afipia* ASV3) in the LS. These greenhouses either shared light infection (approximately 1% of infected plants by the end of the growing season, Table 1 in Additional file 1) or were infected in the previous year. This suggested the existence of an ’intermediate’ state that did not present detectable HRD symptoms.

Our analysis further confirmed the pre-symptomatic sampling time point label. At the first sampling time point, MC7 exhibited significantly higher probability in greenhouses later diagnosed as infected than in healthy greenhouses, and was also elevated in intermediate greenhouses (visualization see Fig. 4 in Additional file 1; statistical test results are provided in Table 2 of Additional file 1). In contrast, MC4 displayed significantly lower probability in both infected and intermediate greenhouses compared to healthy greenhouses.

When comparing the temporal stages, interesting differences in MC distribution were observed (Fig. 4). In the ES, the pattern is the same as observed at pre-symptomatic sampling time point. In the TS, MC0 probability was significantly higher in healthy and intermediate greenhouses compared to infected greenhouses. Conversely, MC8 probability was significantly lower in intermediate greenhouses compared to infected greenhouses. These patterns persisted in the LS, where MC2 probabilities in both intermediate and infected categories were significantly lower than in healthy greenhouses. These findings demonstrate that intermediate greenhouses represent an intermediate state between healthy and infected greenhouses in terms of MC distributions.

These latent signals were only captured through changes in MCs instead of tracking individual parameters of interest, such as the abundance of the *Agrobacterium* pathogen. Correlations between MCs and Ct values of pathogen detection also support this. Particularly, MC7 did not show a significant correlation with the Ct value, which is a measure of pathogen level. This suggests a bacterial community signature that drives infection in which the pathogen remains at low population density. On the other hand, MC4 (r = 0.488) and MC8 (r = $-$0.518) showed significant correlations with Ct values from qPCR targeting Agrobacterium quantification (Table 1). These two correlations align diagnosis results. MC3 (r = $-$0.2) was unique to greenhouse 3 (assigned to the intermediate greenhouse).

**Table 1:** Correlation between MC and *Agrobacterium* abundance as measured by qPCR Ct values

MC	MC 0	MC 1	MC 2	MC 3	MC 4	MC 5	MC 6	MC 7	MC 8
Correlation	0.156	−0.034	−0.045	**−0.200**	**0.488**	**0.175**	0.120	$-0.040$	**−0.518**
*P*-value	0.066	0.690	0.601	**0.018**	**0.000**	**0.038**	0.158	0.639	**0.000**
BH-FDR	0.119	0.690	0.690	0.053	**0.000**	0.086	0.237	0.690	**0.000**

Values are rounded to three decimal places. Values in bold indicate significant correlations ($p< 0.05$). Negative correlation values indicate that samples with a higher probability of the MC have lower Ct values, corresponding to higher *Agrobacterium* population density

### Microbial component - amplicon sequence variant matrix

**Healthy greenhouse-related MCs.** MC4 was characterized by high probabilities of several ASVs belonging to *Paenibacillus*, including *Paenibacillus* ASV11 (0.566), *Paenibacillus* ASV15 (0.078), *Paenibacillus* ASV16 (0.039), and *Paenibacillus* ASV4 (0.015). MC2 was characterized by *Luteimonas* ASV7 (0.020), *Microbacteriaceae* ASV12 (0.016), and *Pseudolabrys* ASV27 (0.017). Additionally, *Paenibacillus* ASV11 (0.125) showed high probability in MC2, along with *Paenibacillus* ASV15 (0.020) and *Micropepsaceae* ASV3 (0.019).

**Infected greenhouse-related MCs.** MC7, an indicator of the ’pre-symptomatic’ sampling time point, showed high probabilities for *Limnobacter* ASV4 (0.032), *Rhizobium* complex ASV29 (0.025), *Flavobacterium* ASV26 (0.017), and *Devosia* ASV5 (0.015); *Paenibacillus* ASV11 (0.101) was high but comparably lower than in MC4, which was also more related to the ES. Among these, *Limnobacter* ASV4 was exclusively high in MC7. MC1 was characterized by *Pseudomonas* spp. ASVs, specifically *Pseudomonas* ASV0 (0.083), *Pseudomonas* ASV2 (0.039), and *Pseudomonas* ASV3 (0.038), along with *Paenibacillus* ASV11 (0.139) and *Paenibacillus* ASV15 (0.020). For the LS-related MCs, MC8 was characterized by *Paenibacillus* ASV11 (0.055), *Streptomyces* ASV4 (0.031), *Rhizobium* complex ASV25 (0.025), *Microbacteriaceae* ASV12 (0.024), and *Devosia* ASV5 (0.017). Notably, the sequence of the *Rhizobium* complex ASV25 matched with the in-house pathogen.

**Intermediate greenhouse-related MCs.** MC0 was characterized by *Paenibacillus* ASV11 (0.082), *Comamonadaceae* ASV65 (0.075), and *Comamonadaceae* ASV70 (0.032) ASVs.

**Other MCs** were exclusive to a certain greenhouse or not associated with a specific health status, including MC3, MC5, and MC6. MC3 was generally found in greenhouse 3 and showed high probabilities for *Paenibacillus* ASV11 (0.068), *Sphingobium* ASV2 (0.026), *Microbacteriaceae* ASV12 (0.019), and *Ohtaekwangia* ASV9 (0.015). MC5 had a high probability of *Acinetobacter* ASV9 (0.065), while MC6 had a high probability of *Paenibacillus* ASV11 (0.129). These two MCs showed low distribution across samples, and their compositions barely overlapped with others (Fig. 3).

### Biomarker discovery in LDA-derived categories

Based on the newly established categories, we conducted LEfSe analysis to identify potential biomarkers [32]. The high linear discriminant analysis scores overlapped with representative ASVs in the MCs (Fig. 6A). Given their meaningful relative abundance levels and characteristic associations with specific MCs, some biomarkers of interest were selected and visualized using modeled data (modeling approach shown in Fig. 2).

**Healthy greenhouses:** ASVs annotated as *Paenibacillus* were identified as potential biomarkers in the ES, all contributing to MC4. The most abundant taxon, *Paenibacillus* ASV11, showed a decreasing trend across all greenhouse types but exhibited higher modeled relative abundance in healthy greenhouses’ ES compared to other categories (Fig. 6B). In the LS, *Pseudolabrys* ASV27 was identified as a biomarker. This taxon showed an increasing trend across greenhouses, with higher modeled relative abundance in healthy greenhouses compared to other greenhouse types in the LS.

**Intermediate greenhouses:** ASVs annotated as *Caulobacter* ASV0 and *Afipia* ASV3 were identified as potential biomarkers in the ES. Although *Caulobacter* ASV0 was statistically identified as a biomarker for intermediate greenhouses, it showed higher modeled relative abundance in healthy greenhouse ES than in the other two categories. Additionally, this taxon had a high probability in MC4, which is characteristic of healthy greenhouse ES. *Afipia* ASV3 exhibited a more variable distribution in intermediate greenhouses compared to the other two categories, where it maintained consistently low modeled relative abundance. This taxon also showed high probability in MC0. In the TS, *Caulobacter* ASV65 displayed higher modeled relative abundance in intermediate greenhouses with variable distribution, a pattern that persisted in the LS.

**Infected greenhouses:** ASVs annotated as *Limnobacter* ASV4 and *Rhizobium* complex ASV25 were identified as biomarkers in the ES; the latter’s sequence also matched the in-house pathogenic rhizogenic *Agrobacterium*. *Limnobacter* ASV4 showed higher modeled relative abundance in infected greenhouses during the ES, exhibited variable distribution, and demonstrated high probability in MC7. *Rhizobium* complex ASV25 showed a similar increasing trend in infected greenhouses while remaining comparably low in intermediate greenhouses and rare in healthy greenhouses. It had a high probability in MC6 and MC8. In the LS, *Rhizobium* complex ASV29 was identified as a biomarker characteristic of MC7, consistently showing higher modeled relative abundance than other categories. Within infected greenhouses, it was slightly higher in the ES but less variable in the LS.

**The BCO-pathogen ratio as a biomarker.** We calculated the ratio between the ASV annotated as *Paenibacillus* ASV11 and the ASV annotated as *Rhizobium* complex ASV25 according to Equation (1), where *R* is the ratio and *A* represents the relative abundance of each ASV across different data: observed, observed with pseudocount adjustment (add $1 \times 10^{-6}$ to both numerator and denominator, see Sect. 2.3.2) and modeled.

1 $$\begin{aligned} R = \frac{A_{\textit{Paenibacillus}~ASV11}}{A_{\textit{Rhizobium}~\text {complex}~ASV25}} \end{aligned}$$

The ratio between these two ASVs was selected because *Paenibacillus* ASV11 was identified as a biomarker in healthy greenhouses’ ES samples by both LDA and LEfSe, while *Rhizobium* complex ASV25 was identified as a biomarker in infected greenhouses’ ES samples and matched with in-house pathogen sequences (other ratio combinations between *Paenibacillus* and *Rhizobium* complex ASVs are available in Additional file 2).

In the observed data, the distribution of log2-transformed *R* values differed significantly among the three greenhouse types in the LS (Fig. 7; see distribution of untransformed *R* values in Fig. 6 in Additional file 1). Both healthy and intermediate greenhouse samples, with median values of 6.9 and 6.98, respectively, were significantly higher than infected greenhouse samples (median = 3.03). Following pseudocount adjustment, these patterns persisted, but distribution was elevated. This elevation resulted from the pseudocount addition preventing zero denominators, which avoided statistical bias introduced by discarding infinity values. Additionally, the distribution among healthy and intermediate greenhouse samples became significantly different from infected greenhouse samples in the ES.

For the modeled data, log2-transformed *R* distributions differed significantly among all three greenhouse types in both the ES and LS, with all pairwise comparisons showing significant differences. In the ES, median *R* values were highest for healthy greenhouse samples (8.66), followed by intermediate samples (7.78), and lowest for infected samples (6.66). Similarly, in the LS, healthy greenhouse samples had the highest median value (8.97), while intermediate and infected samples had median values of 6.42 and 3.22, respectively.

## Discussion

### ASV-based LDA highlights bacterial community signatures of HRD infection

The analysis of amplicon sequencing data using an LDA approach yielded more detailed insights into the bacterial community associated with tomato rockwool and root samples. Regarding temporal patterns, the current study grouped the five sampling time points in three stages, namely early, transition, and late stage. Greenhouses annotated as healthy were associated with MC4, which is enriched with *Paenibacillus* sequences, in contrast to greenhouses annotated as infected, which were mainly associated with MC7 that was enriched with rhizogenic *Agrobacterium*, *Limnobacter*, and *Devosia* sequences, with *Limnobacter* being more prevalent during the ES. This suggests two insights: (i) possible indicator organisms in addition to rhizogenic *Agrobacterium*;(ii) infected greenhouses harboured potential pathogen inoculum already at the start of the crop cycle. Both of them emphasizing the need for management strategies at this stage. Chemical disinfection, including treatment with sodium hypochlorite or hydrogen peroxide, is usually conducted before placing tomato seedlings in hydroponic greenhouses [42, 43]. Alternatively, the application of biocontrol agents such as *Paenibacillus* spp. at the establishment of the crop has produced positive effects, reducing HRD infection pressure down to 4% [3]. Either way, this new analysis approach could be highly valuable for growers and assist in decision-making of treatments.

Indeed, the current LDA approach allowed the determination of two new infection statuses that provide additional information regarding diagnostics, namely“pre-symptomatic”with higher probabilities of MC7 and“intermediate”with a high probability of MC0 in the TS and LS. Greenhouses classified as intermediate presented a light infection incidence (2% in greenhouse 2; 1% in greenhouse 12) or had an infection episode a year prior (2017) to sample collection (in the case of greenhouses 3 and 6). Unlike samples from healthy greenhouses at the ES, samples from intermediate greenhouses showing higher MC7 probability. Remarkably, MC7 also included ASVs assigned to *Paenibacillus*, the genus of the identified BCO. The limited protection against HRD in these cases could be explained by the lower abundance of *Paenibacillus* BCO strains. Alternatively, it has been shown that only a limited number of *Paenibacillus* species show activity against *Agrobacterium*, and possibly in these cases inactive *Paenibacillus* ASVs are detected. This aligns with the ratio (*R*) distribution in intermediate greenhouses (Fig. 7). However, intermediate samples had higher probabilities of MC0 by the LS. This demonstrates that previous year infections increase the risk of suboptimal microbiomes in subsequent seasons. These MCs could be considered as core microbiota that drive plant health or disease, providing predictive value even before visible symptoms appear.

Pre-symptomatic samples were characterized by higher probabilities of MC7, containing members of the *Rhizobium* complex. These results agree with Vanlommel et al. [44], who suggested that rhizogenic *Agrobacterium* need to exceed $3.2 \times 10^{3}$ CFU mL$^{-1}$ at 17 weeks after planting to have a 50% chance of plants showing visual symptoms a week later. Also in other studies, negative significant correlations based on co-occurrence network analysis have been observed, e.g. between pathogen *Fusarium* spp. responsible for *Fusarium* head blight in maize and candidate antagonistic bacterial (*Sphingomonas*) and fungal (*Sarocladium* and *Epicoccum*) OTUs [45].

A core collection of biocontrol bacteria may be present in MC4 and MC0, including *Paenibacillus*, and *Caulobacter*. While some *Paenibacillus* strains have shown great promise as a biocontrol agent against rhizogenic agrobacteria [2, 7], *Caulobacter* showed biocontrol activity against bacterial wilt (caused by *Ralstonia solanaceaerum*) [46] and has demonstrated plant growth-promoting activity in varied crops including soybean, corn, and wheat [47]. Members of the *Comamonadaceae* have been found in the rhizosphere of healthy tomato and cucumber, while absent in plants affected by Fusarium wilt disease, suggesting biocontrol activity [48, 49]. Furthermore, members of this family are considered beneficial bacteria transferred through the seed of tomato, highlighting its importance for the plant holobiont [50]. The novel insights obtained in this study are extremely valuable for designing biocontrol strategies. For instance, these three bacterial genera could be considered strong candidates to be included in a synthetic microbial communities with protective activity for hydroponic tomato against HRD, and possibly other plant diseases, such as Fusarium wilt.

Similarly, MC7 and MC8 could be considered a core bacterial community of HRD-inducive bacteria, including *Devosia*, *Limnobacter*, *Streptomyces*, and undescribed members of the *Microbacteraceae* family, showing probabilities of 0.03$-$0.12. Interestingly, in another study *Limnobacter* has also been found in high abundance $(\sim 10\%)$ in the nutrient solution of tomato hydroponic greenhouses infected with wildfire leaf disease caused by *Pseudomonas syringae* [51]. Intriguingly, *Paenibacillus* was also part of the so-called core collection of HRD-inducive bacteria, albeit in lower proportions (0.06$-$0.012) in contrast to its high relative abundance in MC4 (0.76). This highlights the possibility of not only a threshold of rhizogenic *Agrobacterium* needed to produce an HRD infection, but also of *Paenibacillus* to have effective biocontrol activity. This is supported by the modeled ratio between *Paenibacillus* and rhizogenic *Agrobacterium*, which differed significantly among greenhouse categories in the ES, with healthy greenhouses showing the highest ratio followed by intermediate, and lowest in infected greenhouses (Fig. 7). This pattern was even more pronounced in the LS samples. This same pattern was observed in the LS and aligns with similar findings in other pathosystems [52].

Furthermore, our results suggest that MC1-containing ASVs identified as *Pseudomonas* were more likely to occur in GH1’s ES (the most heavily infested greenhouse) and GH4’s LS (the least heavily infested greenhouse). GH1 showed the MC trajectory from MC1 in the ES to MC8 in the LS, GH4 switched from MC7 in the ES to the MC1 in the LS. This associated with findings that reported a reduction in HRD in hydroponically grown tomato plants following the application of three *Pseudomonas* strains: *P. protegens* 1B1, *P. brassicacearum* 93G8, and *P. chlororaphis* 48G9 [53]. It is possible that the *Pseudomonas* ASVs detected in MC1 were recruited BCO strains during infection, or it represent different strains from those studied by de Freitas et al. [53].

### Visual analytics in plant microbiota research

The growing complexity of microbiota data has exposed the limitations of linear analytical algorithms such as PCoA [54]. This challenge requires both innovative computational approaches and greater integration of domain expertise in the analytical workflow. Meanwhile, plant microbiota research remains comparatively under-explored compared to human gut microbiota studies. This knowledge gap creates both challenges and opportunities for discovery. The strength of applying LDA in our study lies in how human interpretation guided the computational analysis of field-derived data. This approach enabled us to translate patterns into biological insights while generating meaningful hypotheses.

First, we illustrated how to identify new patterns through a data-driven approach. In microbiota applications, annotated heatmaps primarily demonstrate LDA’s ability to distinguish health status [17], potentially overlooking its capacity to discover latent sample groups. Our interactive Sample-MC heatmap (Fig. 3) revealed such latent groups. Though this approach becomes cluttered with more than five metadata variables and needs refinement, we addressed an important gap by creating accessible visualizations for LDA applications in understudied subfields [12].

Second, the patterns from LDA drove new insights. Biomarker analysis benefited from newly established labels, as reported in Sect. 3 and discussed in Sect. 4.1. These observations and insights remained undetected in previous analyses using infected/healthy binary categories, further emphasizing the methodology for handling complexity.

Beyond biological insights and methodology, this study generates valuable hypotheses. More specifically, (i) greenhouse4 (with the lightest infection) exhibited a transition from MC7 to MC1, raising the question of whether a progressive infection pathway exists following the sequence MC7$\rightarrow$MC1$\rightarrow$MC8. (ii) Given that *Pseudomonas* (main members of MC1) is an established biocontrol agent [53, 55], we hypothesize these organisms are recruited in response to pathogen stress. (iii) Intermediate greenhouses showed moderate probability of MC4 and MC7 in the ES and high MC0 probability in the LS, suggesting BCO-to-pathogen ratios indicate plant health in the ES and highlighting MC0’s biocontrol potential in the LS.

## Conclusion

We employed LDA within a visual analytics framework to study tomato root-associated microbiota in hydroponic greenhouses affected by HRD. Our analysis revealed structured temporal changes in microbial components and provided insights into complex plant-microbe interactions beyond single-strain tracking. These findings can enhance greenhouse monitoring, allowing more accurate and early prediction of disease onset, and contribute to improved biocontrol strategies. From a computational perspective, we demonstrate how to apply LDA-a powerful analytical tool-to understudied subfields by creating accessible visualizations.

## Additional file

Additional file 1.

Additional file 2.

Additional file 3.

Additional file 4.

## Data Availability

The sequences used in this study were deposited in the Sequence Read Archive (SRA) at NCBI under Bioproject PRJNA734858. The sequence data supporting the conclusions of this article are available in the BioProject PRJNA734858 repository at NCBI. The processed feature table obtained from QIIME2 is included within the article and its additional file 4. Relative abundance table, modeled abundance table, LDA implementation and other analysis scripts are available at https://gitlab.kuleuven.be/aida-lab/public/LDA4HRD
