# Supplementary material for: Latent Dirichlet Allocation reveals tomato root-associated bacterial interactions responding to hairy root disease
Source: Environ Microbiome. 2025 Nov 23;20:161. doi: 10.1186/s40793-025-00822-2 (PMC12751256; doi:10.1186/s40793-025-00822-2)
Supplement: Supplementary file 1 — Additional file 1. [file 40793_2025_822_MOESM1_ESM.docx]

## Supplementary Figures:


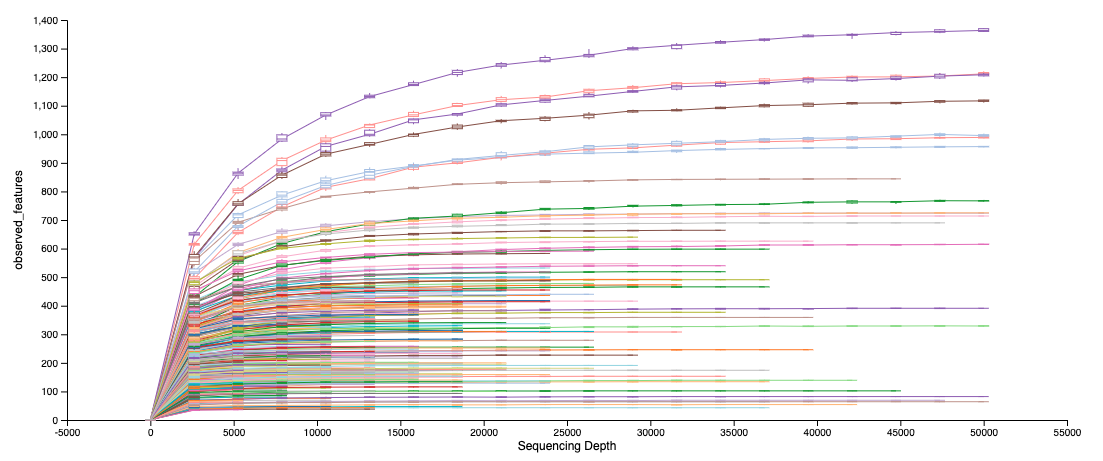


Fig. 1: Rarefaction curves created in Qiime2 by using qiime diversity alpha-rarefaction command.


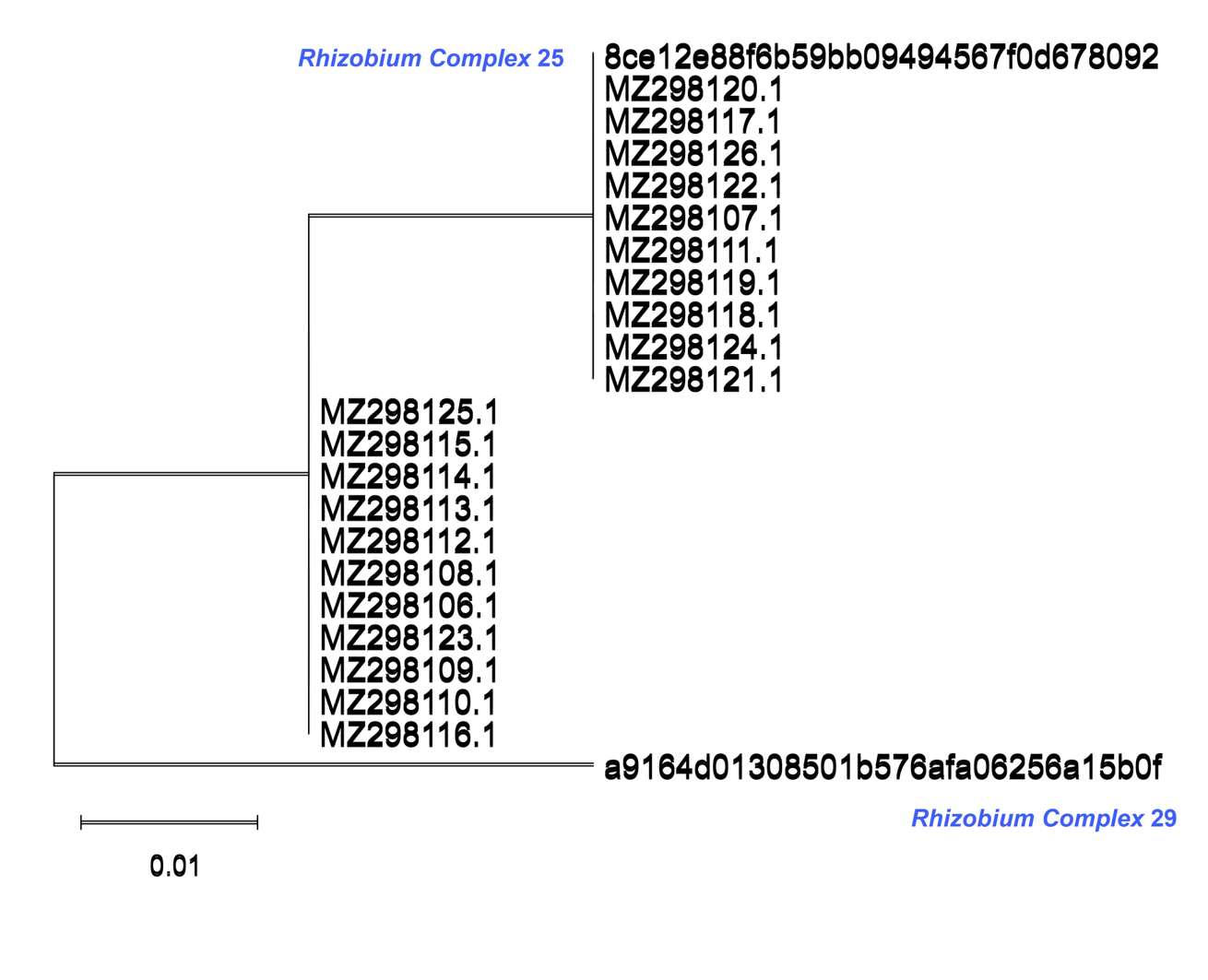
Fig.2: Pathogen sequence matching. ASV annotated as Rhizobium Complex 25 match with the 16S rRNA V4 sequence of previously identified rhizogenic Agrobacterium bv. 1 strain isolated from HRD-infested greenhouses.


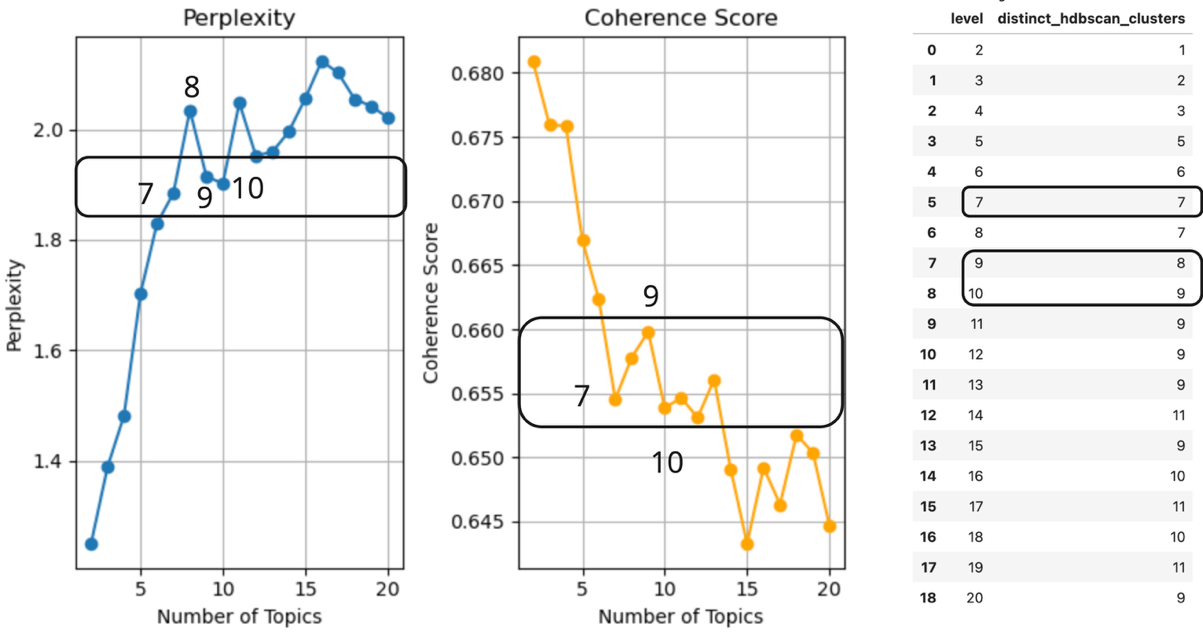


Fig. 3: Metrics of model selection, from left to right are Perplexity, Coherence Score and Persistent MC clusters within each model.


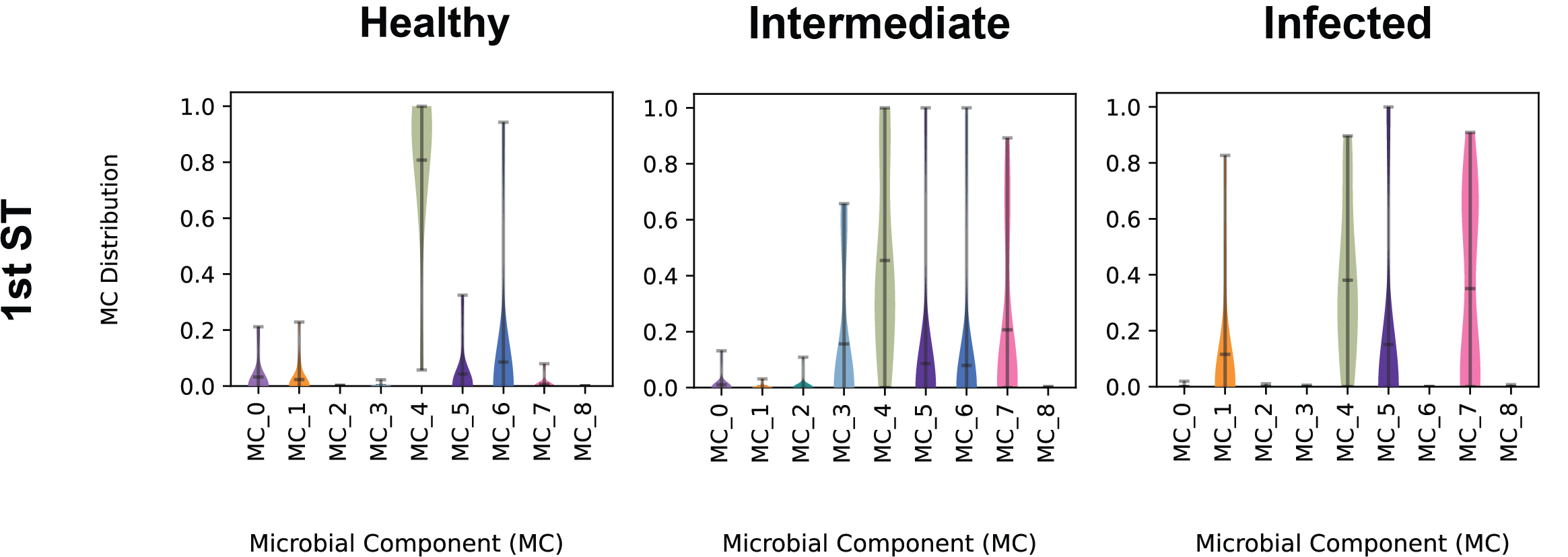


Fig. 4: MC distribution among three different types of greenhouses in the first sampling time point.


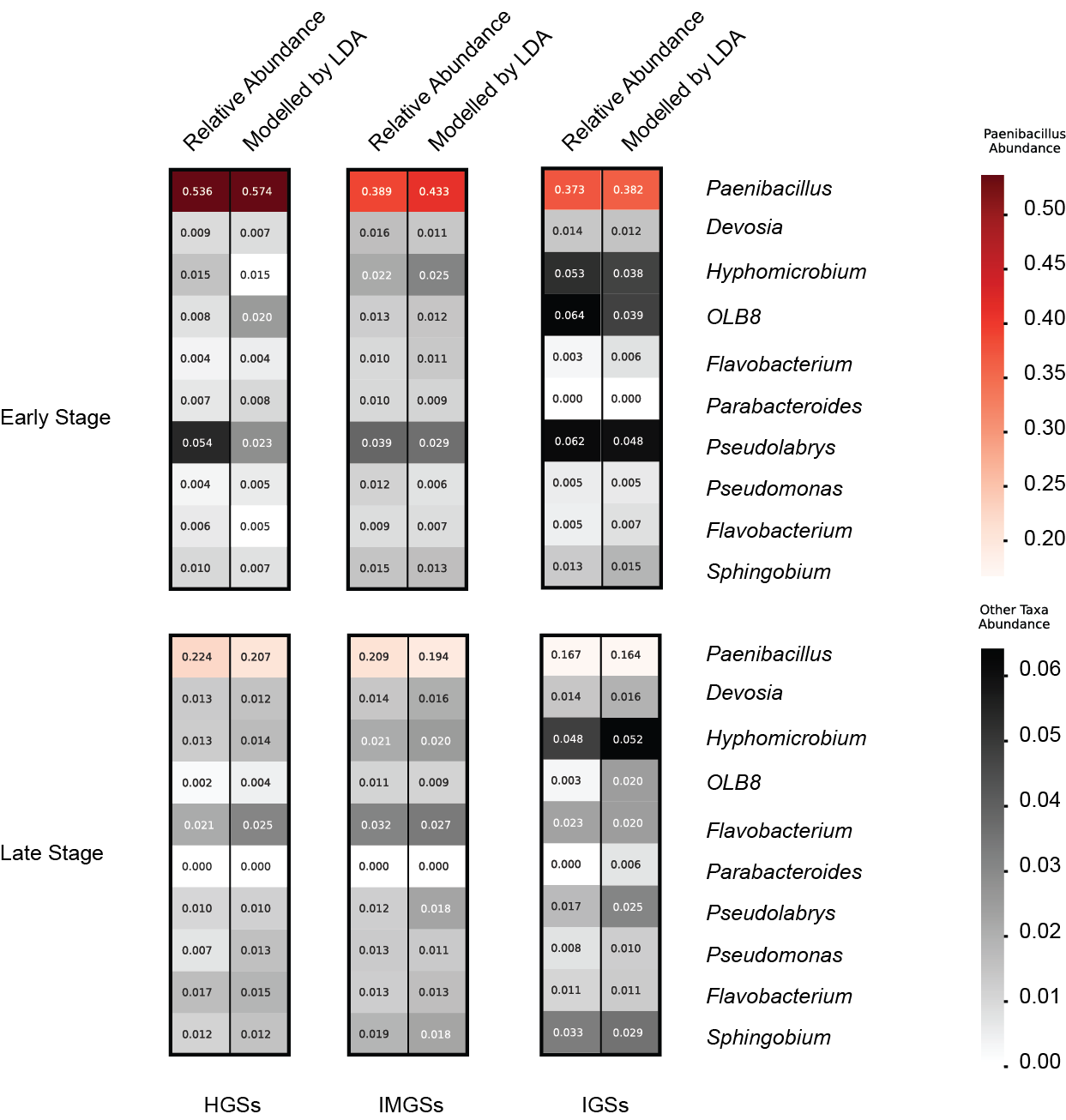


Fig.5: Comparison between relative abundance and modelled relative abundance by LDA regard to top ten bacterial genera. HGSs: healthy greenhouses samples; IMGSs: intermediate greenhouses samples; IGSs: infected greenhouses samples.


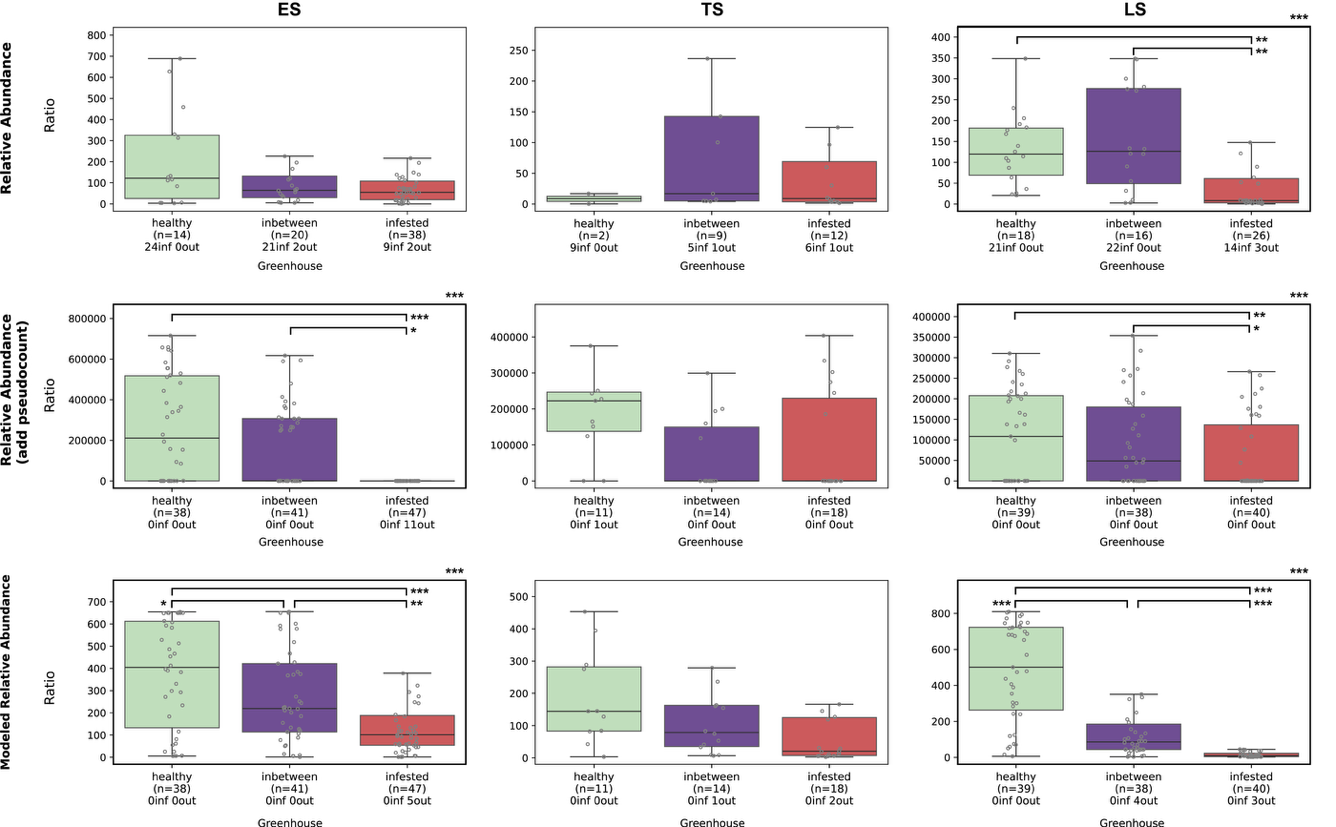


Fig.6: Abundance ratios of *Paenibacillus* ASV11 to *Rhizobium* complex ASV25 across greenhouse categories. It shares the same layout as Fig. 7 in the main text.

Supplementary Tables:

Table 1: Greenhouses’ metadata

Table 2: statistical test of temporal and diagnosis patterns. Only significant results are listed.
