# Supplementary material for: Latent Dirichlet Allocation reveals tomato root-associated bacterial interactions responding to hairy root disease
Source: Environ Microbiome. 2025 Nov 23;20:161. doi: 10.1186/s40793-025-00822-2 (PMC12751256; doi:10.1186/s40793-025-00822-2)

Abundance Ratio Analysis: Paenibacillus_11 to Rhizobium_complex_25

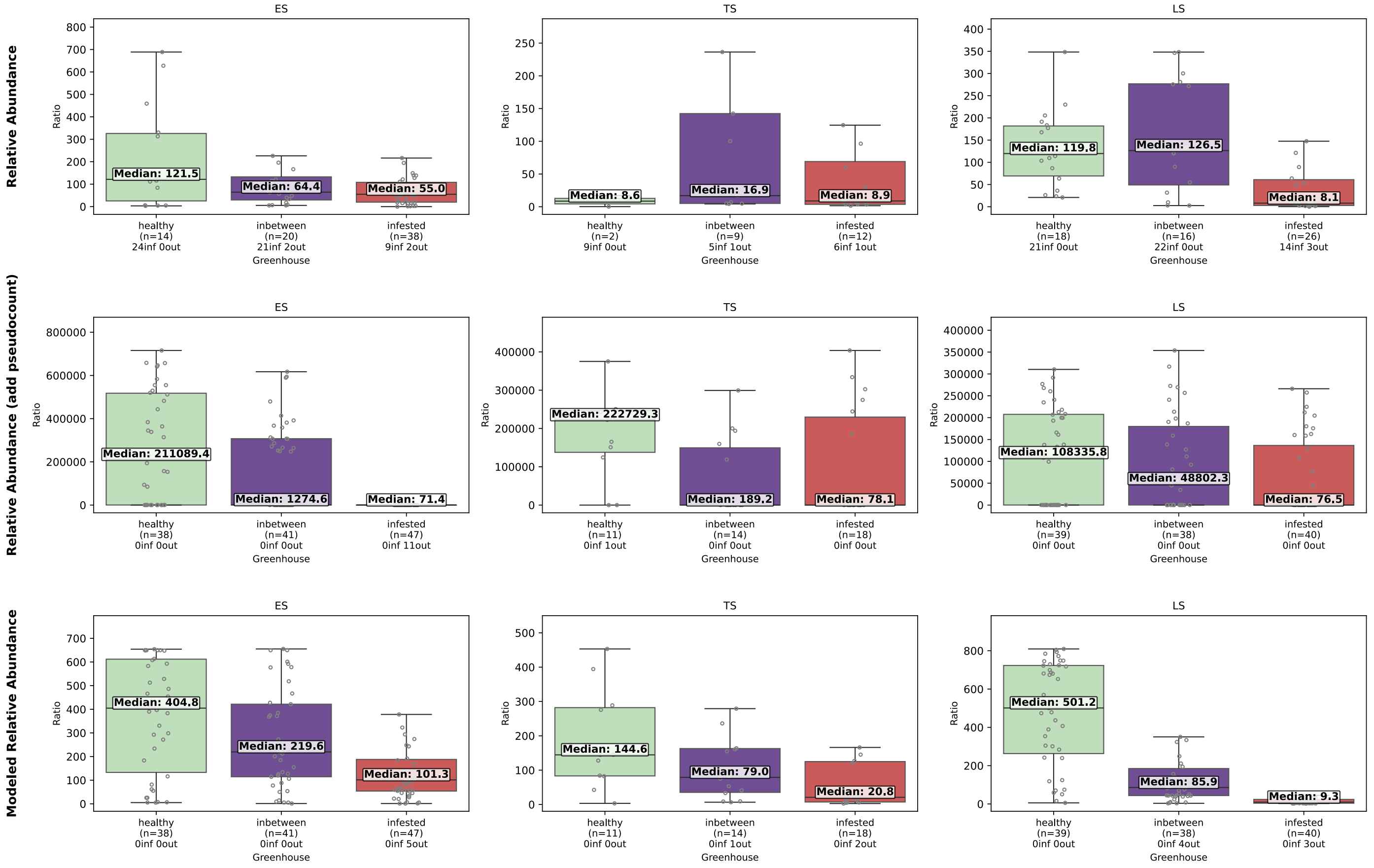

Abundance Ratio Analysis: Paenibacillus_11 to Rhizobium_complex_29

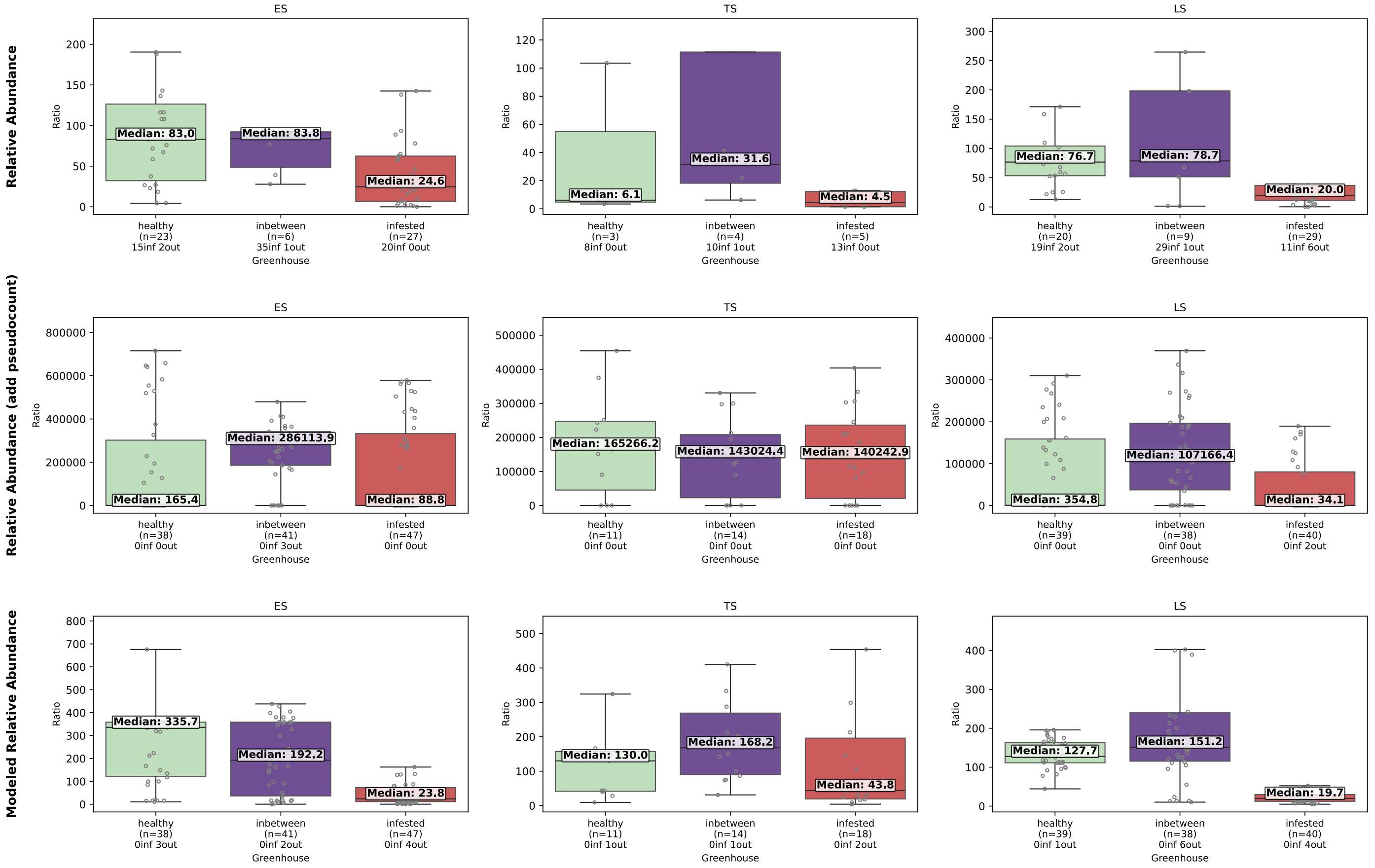

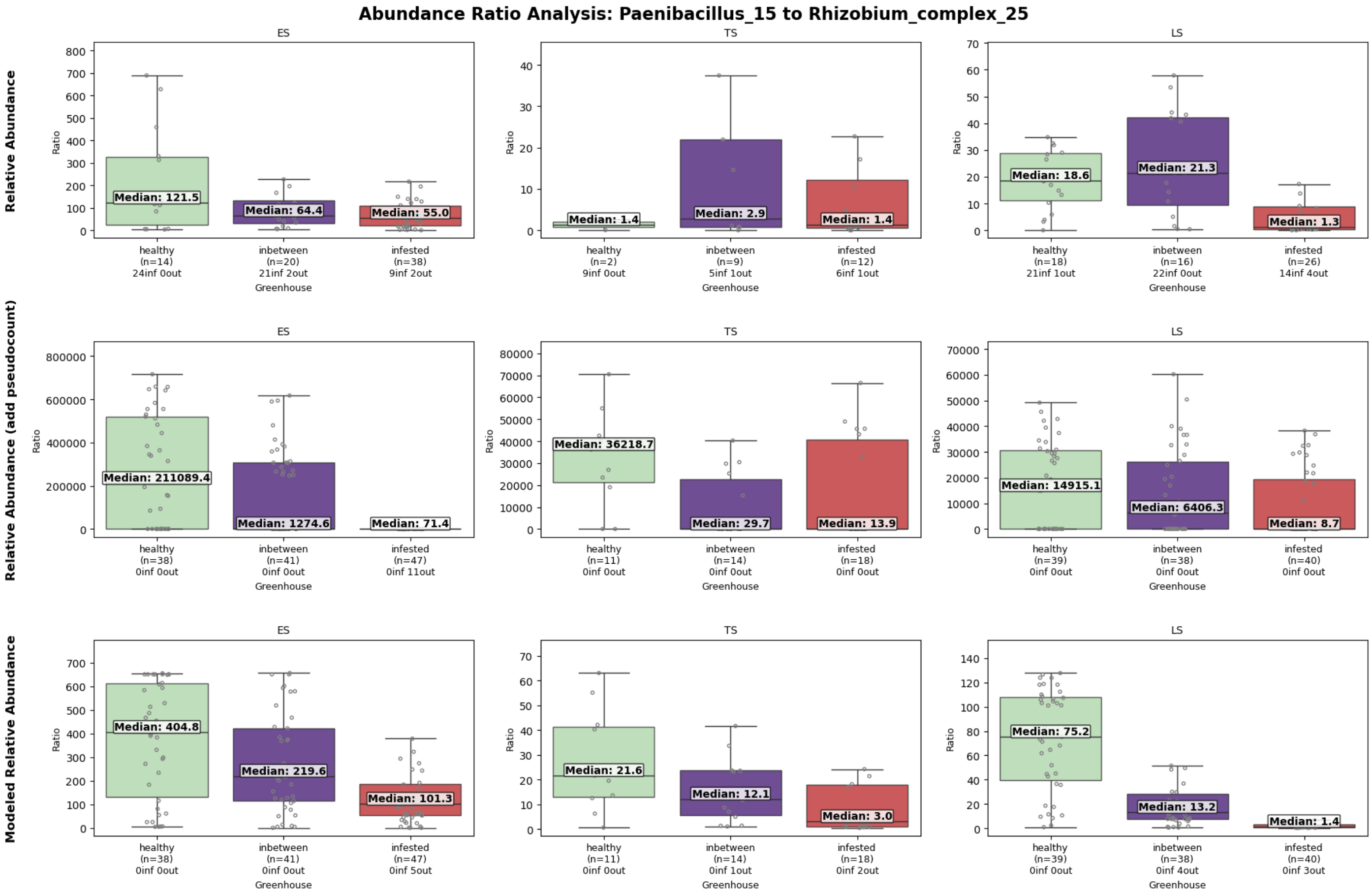
Abundance Ratio Analysis: Paenibacillus_15 to Rhizobium_complex_25
ES
TS
LS
Relative Abundance
Relative Abundance (add pseudocount)
Modeled Relative Abundance
Ratio
Greenhouse
Median: 121.5
Median: 64.4
Median: 55.0
healthy (n=14) 24inf 0out
inbetween (n=20) 21inf 2out
infested (n=38) 9inf 2out
Median: 1.4
Median: 2.9
Median: 1.4
healthy (n=2) 9inf 0out
inbetween (n=9) 5inf 1out
infested (n=12) 6inf 1out
Median: 18.6
Median: 21.3
Median: 1.3
healthy (n=18) 21inf 1out
inbetween (n=16) 22inf 0out
infested (n=26) 14inf 4out
Median: 211089.4
Median: 1274.6
Median: 71.4
healthy (n=38) 0inf 0out
inbetween (n=41) 0inf 0out
infested (n=47) 0inf 11out
Median: 36218.7
Median: 29.7
Median: 13.9
healthy (n=11) 0inf 0out
inbetween (n=14) 0inf 0out
infested (n=18) 0inf 0out
Median: 14915.1
Median: 6406.3
Median: 8.7
healthy (n=39) 0inf 0out
inbetween (n=38) 0inf 0out
infested (n=40) 0inf 0out
Median: 404.8
Median: 219.6
Median: 101.3
healthy (n=38) 0inf 0out
inbetween (n=41) 0inf 0out
infested (n=47) 0inf 5out
Median: 21.6
Median: 12.1
Median: 3.0
healthy (n=11) 0inf 0out
inbetween (n=14) 0inf 1out
infested (n=18) 0inf 2out
Median: 75.2
Median: 13.2
Median: 1.4
healthy (n=39) 0inf 0out
inbetween (n=38) 0inf 4out
infested (n=40) 0inf 3out

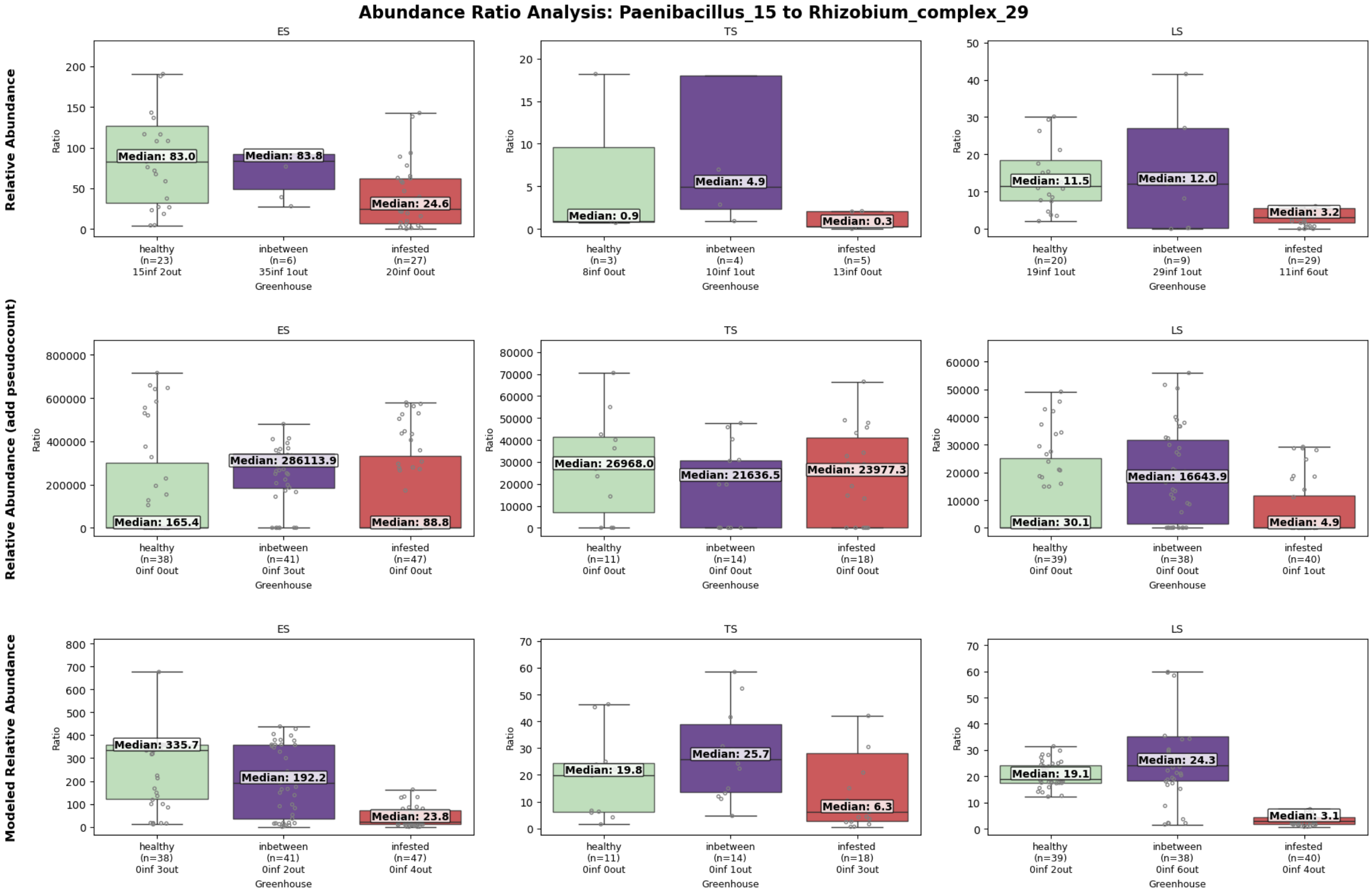
Abundance Ratio Analysis: Paenibacillus_15 to Rhizobium_complex_29
ES
TS
LS
Relative Abundance
Relative Abundance (add pseudocount)
Modeled Relative Abundance
Ratio
Greenhouse
Median: 83.0
Median: 83.8
Median: 24.6
healthy (n=23) 15inf 2out
inbetween (n=6) 35inf 1out
infested (n=27) 20inf 0out
Median: 0.9
Median: 4.9
Median: 0.3
healthy (n=3) 8inf 0out
inbetween (n=4) 10inf 1out
infested (n=5) 13inf 0out
Median: 11.5
Median: 12.0
Median: 3.2
healthy (n=20) 19inf 1out
inbetween (n=9) 29inf 1out
infested (n=29) 11inf 6out
Median: 165.4
Median: 286113.9
Median: 88.8
healthy (n=38) 0inf 0out
inbetween (n=41) 0inf 3out
infested (n=47) 0inf 0out
Median: 26968.0
Median: 21636.5
Median: 23977.3
healthy (n=11) 0inf 0out
inbetween (n=14) 0inf 0out
infested (n=18) 0inf 0out
Median: 30.1
Median: 16643.9
Median: 4.9
healthy (n=39) 0inf 0out
inbetween (n=38) 0inf 0out
infested (n=40) 0inf 1out
Median: 335.7
Median: 192.2
Median: 23.8
healthy (n=38) 0inf 3out
inbetween (n=41) 0inf 2out
infested (n=47) 0inf 4out
Median: 19.8
Median: 25.7
Median: 6.3
healthy (n=11) 0inf 0out
inbetween (n=14) 0inf 1out
infested (n=18) 0inf 3out
Median: 19.1
Median: 24.3
Median: 3.1
healthy (n=39) 0inf 2out
inbetween (n=38) 0inf 6out
infested (n=40) 0inf 4out

Abundance Ratio Analysis: Paenibacillus_16 to Rhizobium_complex_25

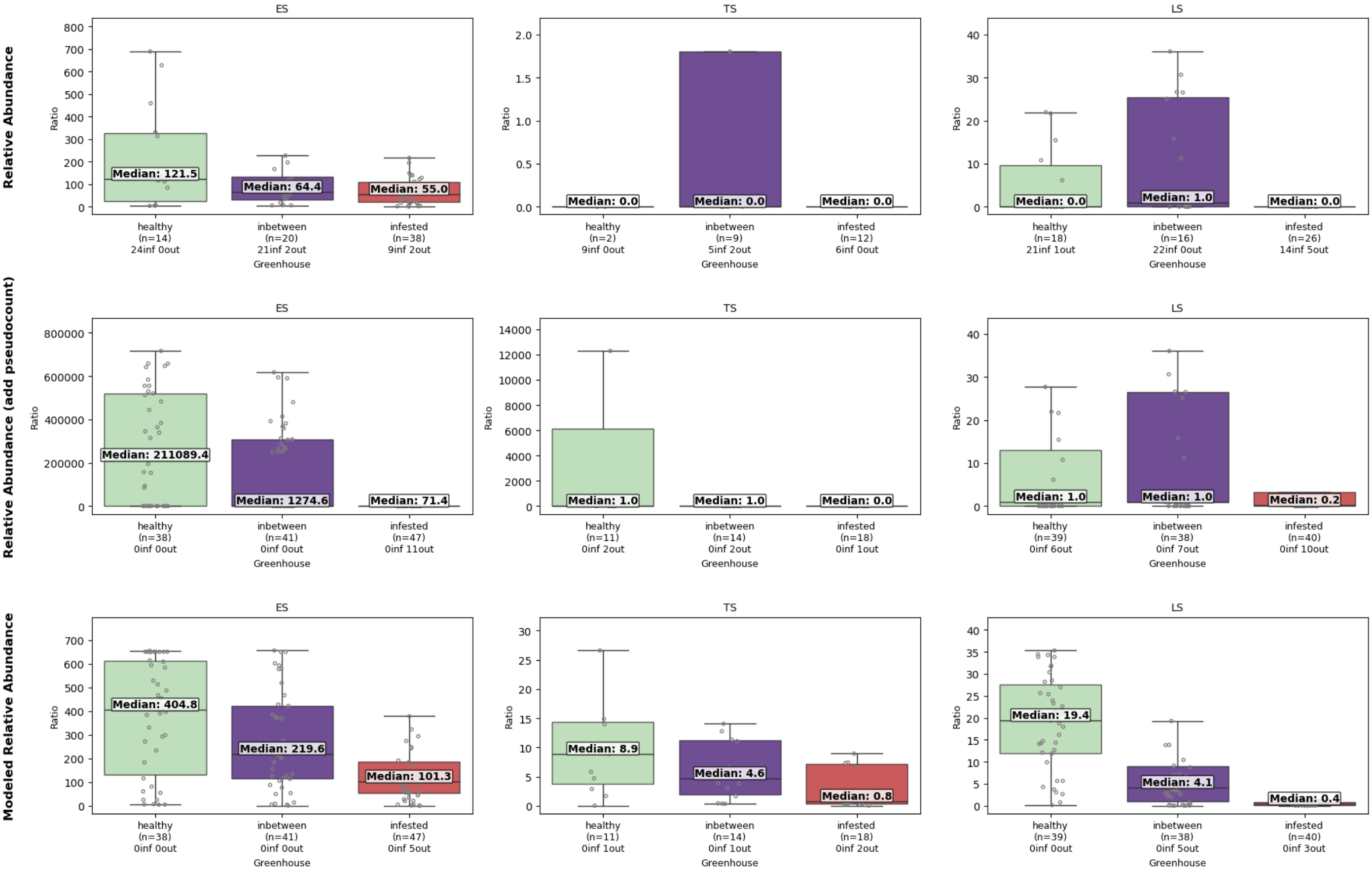

Abundance Ratio Analysis: Paenibacillus_16 to Rhizobium_complex_29

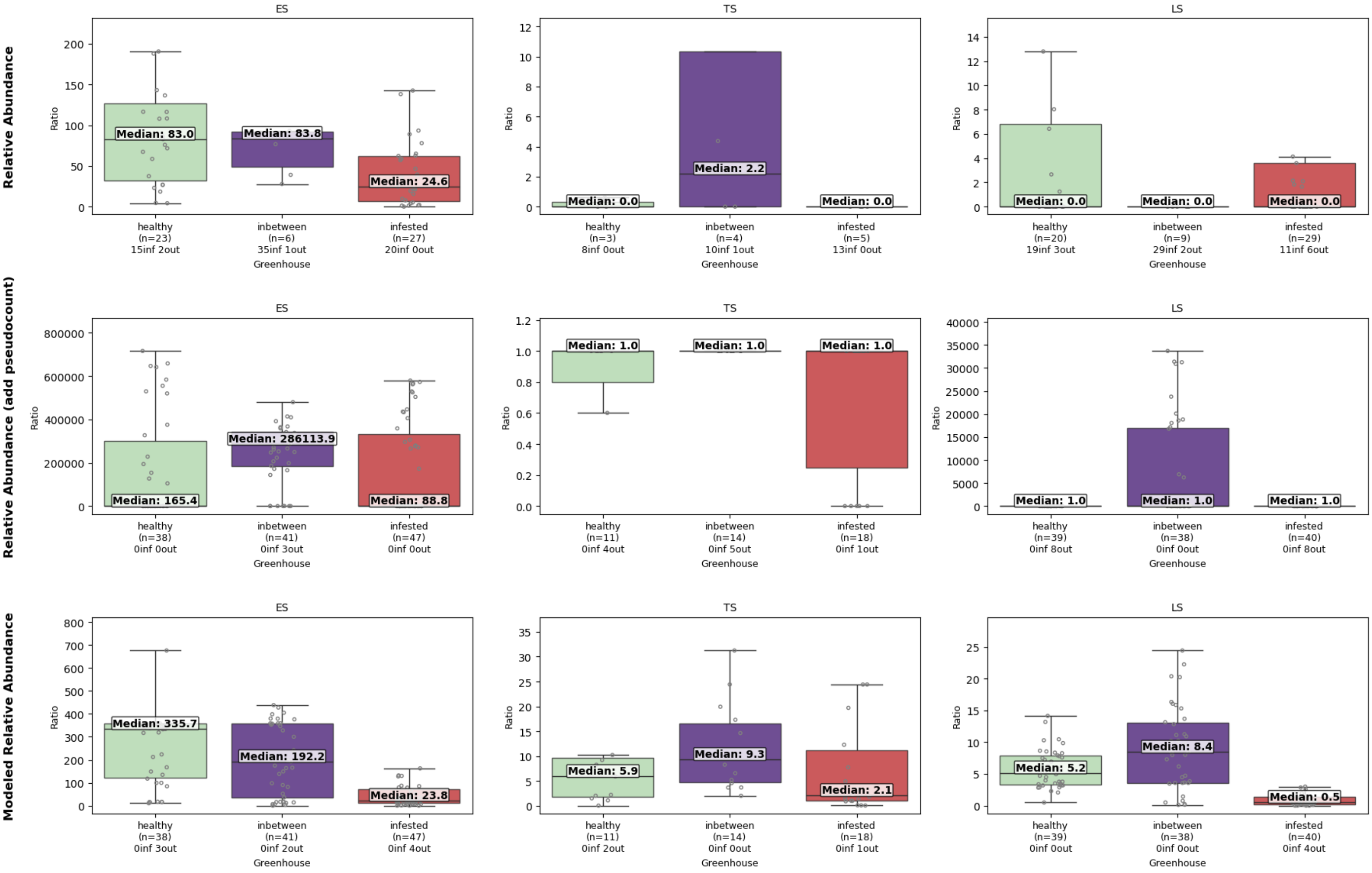

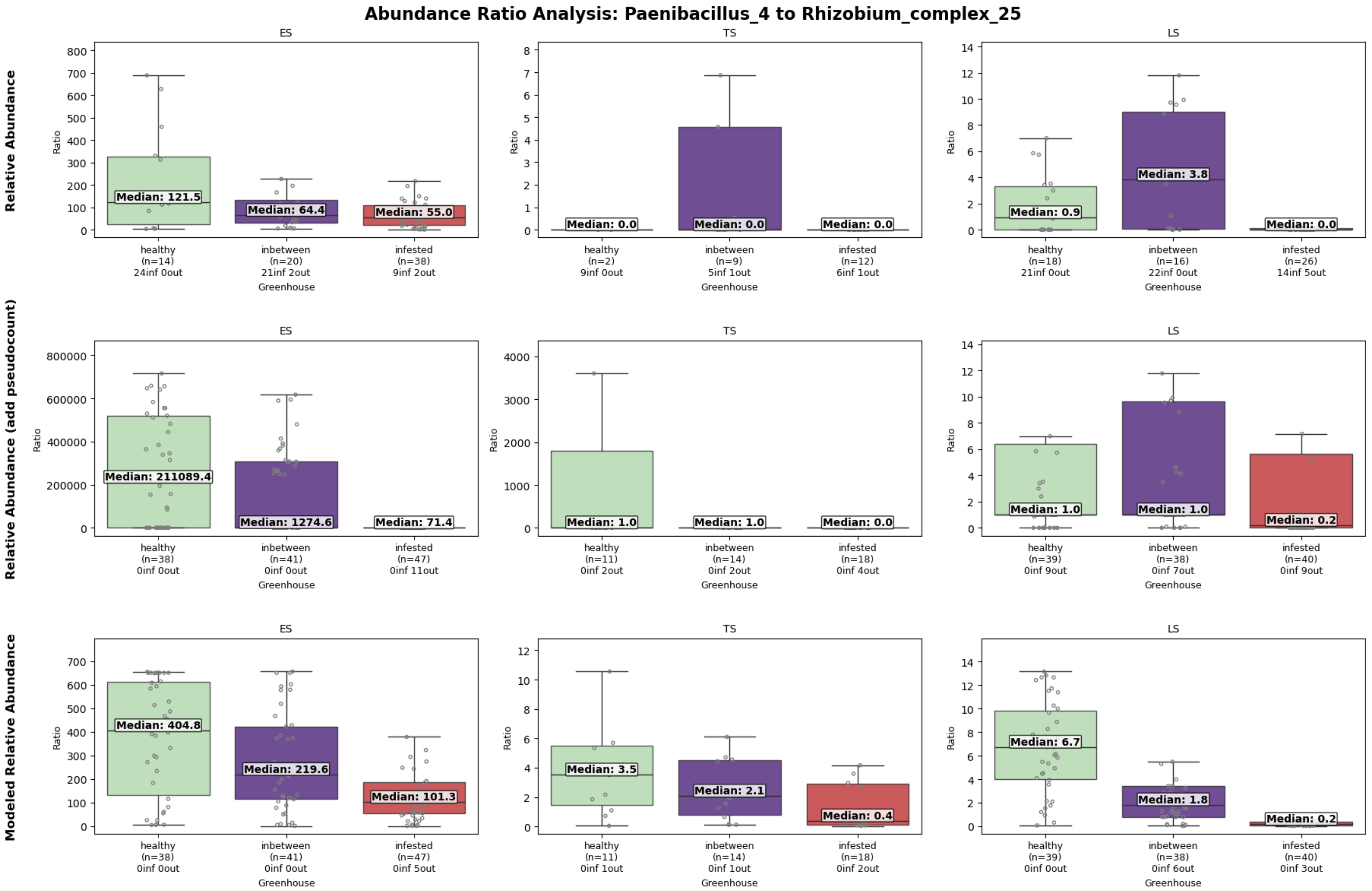
Abundance Ratio Analysis: Paenibacillus_4 to Rhizobium_complex_25
Relative Abundance
Relative Abundance (add pseudocount)
Modeled Relative Abundance
ES
TS
LS
Ratio
Greenhouse
Median: 121.5
Median: 64.4
Median: 55.0
healthy (n=14) 24inf 0out
inbetween (n=20) 21inf 2out
infested (n=38) 9inf 2out
Median: 0.0
healthy (n=2) 9inf 0out
inbetween (n=9) 5inf 1out
infested (n=12) 6inf 1out
Median: 0.9
Median: 3.8
healthy (n=18) 21inf 0out
inbetween (n=16) 22inf 0out
infested (n=26) 14inf 5out
Median: 211089.4
Median: 1274.6
Median: 71.4
healthy (n=38) 0inf 0out
inbetween (n=41) 0inf 0out
infested (n=47) 0inf 11out
Median: 1.0
healthy (n=11) 0inf 2out
inbetween (n=14) 0inf 2out
infested (n=18) 0inf 4out
Median: 0.2
healthy (n=39) 0inf 9out
inbetween (n=38) 0inf 7out
infested (n=40) 0inf 9out
Median: 404.8
Median: 219.6
Median: 101.3
healthy (n=38) 0inf 0out
inbetween (n=41) 0inf 0out
infested (n=47) 0inf 5out
Median: 3.5
Median: 2.1
Median: 0.4
healthy (n=11) 0inf 1out
inbetween (n=14) 0inf 1out
infested (n=18) 0inf 2out
Median: 6.7
Median: 1.8
Median: 0.2
healthy (n=39) 0inf 0out
inbetween (n=38) 0inf 6out
infested (n=40) 0inf 3out

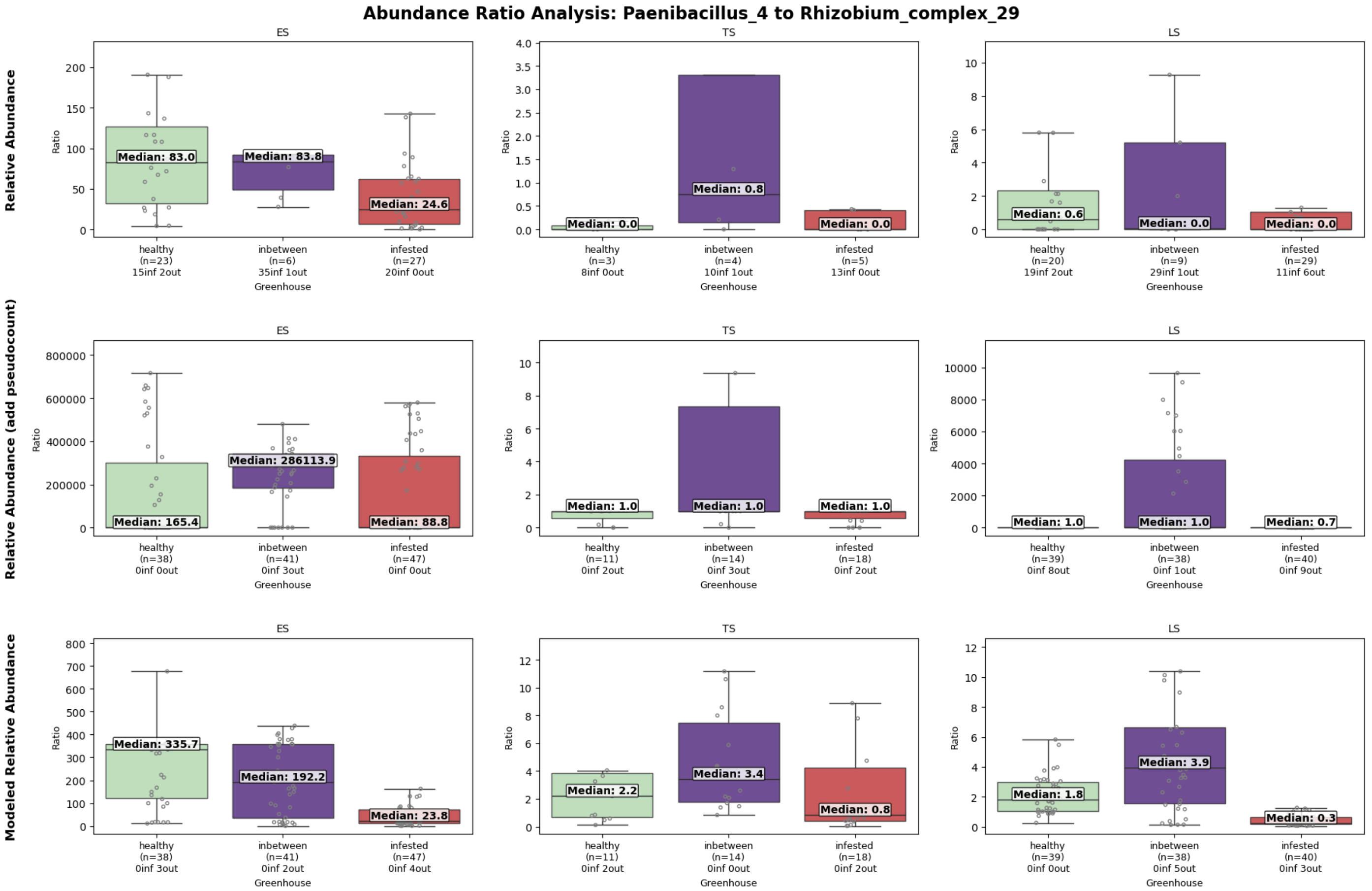
Abundance Ratio Analysis: Paenibacillus_4 to Rhizobium_complex_29
Relative Abundance
Relative Abundance (add pseudocount)
Modeled Relative Abundance
ES
TS
LS
Ratio
Greenhouse
Median: 83.0
Median: 83.8
Median: 24.6
healthy (n=23) 15inf 2out
inbetween (n=6) 35inf 1out
infested (n=27) 20inf 0out
Median: 0.0
Median: 0.8
healthy (n=3) 8inf 0out
inbetween (n=4) 10inf 1out
infested (n=5) 13inf 0out
Median: 0.6
healthy (n=20) 19inf 2out
inbetween (n=9) 29inf 1out
infested (n=29) 11inf 6out
Median: 165.4
Median: 286113.9
Median: 88.8
healthy (n=38) 0inf 0out
inbetween (n=41) 0inf 3out
infested (n=47) 0inf 0out
Median: 1.0
healthy (n=11) 0inf 2out
inbetween (n=14) 0inf 3out
infested (n=18) 0inf 2out
Median: 0.7
healthy (n=39) 0inf 8out
inbetween (n=38) 0inf 1out
infested (n=40) 0inf 9out
Median: 335.7
Median: 192.2
Median: 23.8
healthy (n=38) 0inf 3out
inbetween (n=41) 0inf 2out
infested (n=47) 0inf 4out
Median: 2.2
Median: 3.4
healthy (n=11) 0inf 2out
inbetween (n=14) 0inf 0out
infested (n=18) 0inf 2out
Median: 1.8
Median: 3.9
Median: 0.3
healthy (n=39) 0inf 0out
inbetween (n=38) 0inf 5out
infested (n=40) 0inf 3out

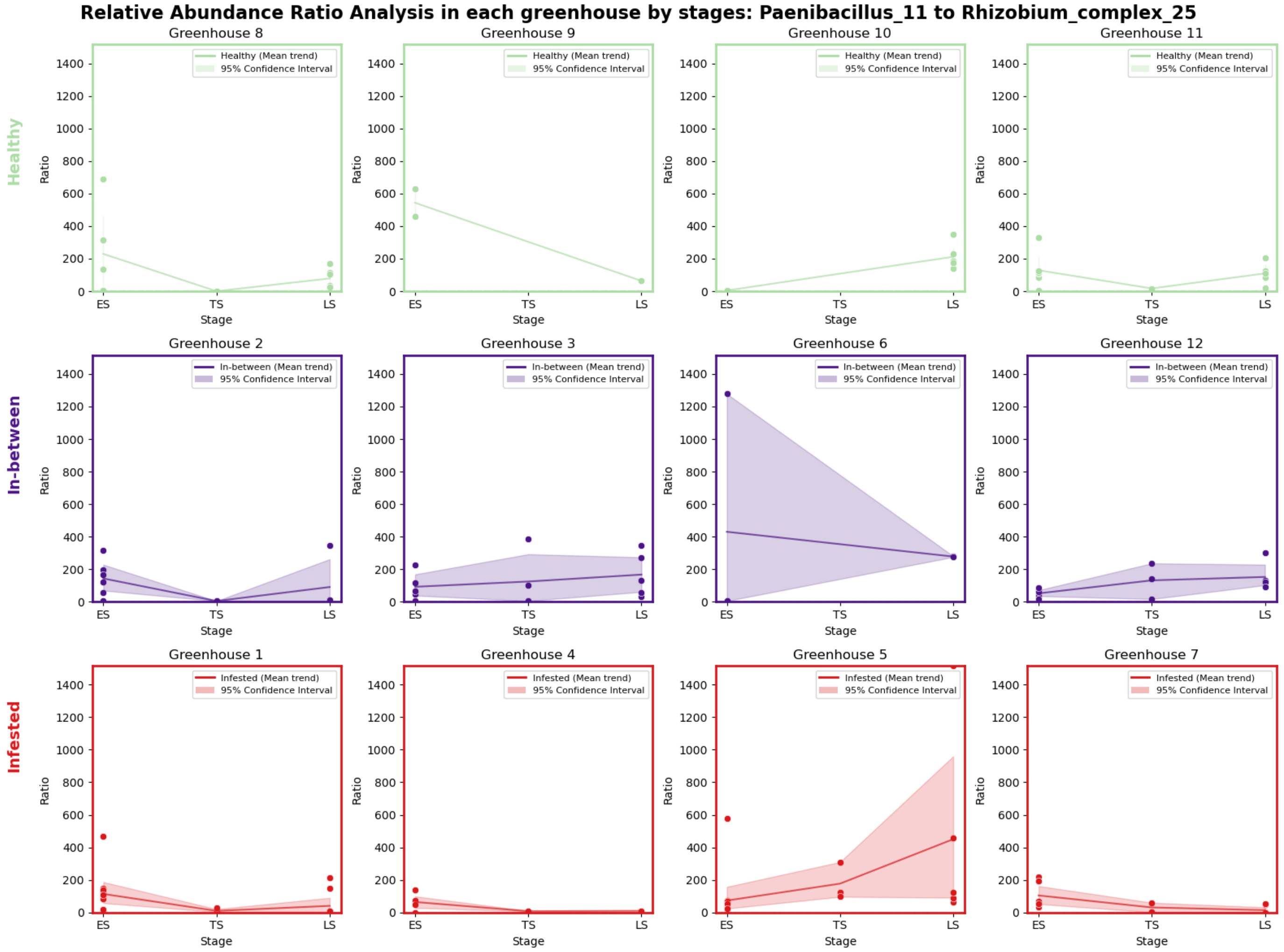
Relative Abundance Ratio Analysis in each greenhouse by stages: Paenibacillus_11 to Rhizobium_complex_25
Greenhouse 8
Greenhouse 9
Greenhouse 10
Greenhouse 11
Greenhouse 2
Greenhouse 3
Greenhouse 6
Greenhouse 12
Greenhouse 1
Greenhouse 4
Greenhouse 5
Greenhouse 7
Healthy
In-between
Infested
Healthy (Mean trend)
In-between (Mean trend)
Infested (Mean trend)
95% Confidence Interval
Ratio
Stage
ES
TS
LS
0
200
400
600
800
1000
1200
1400

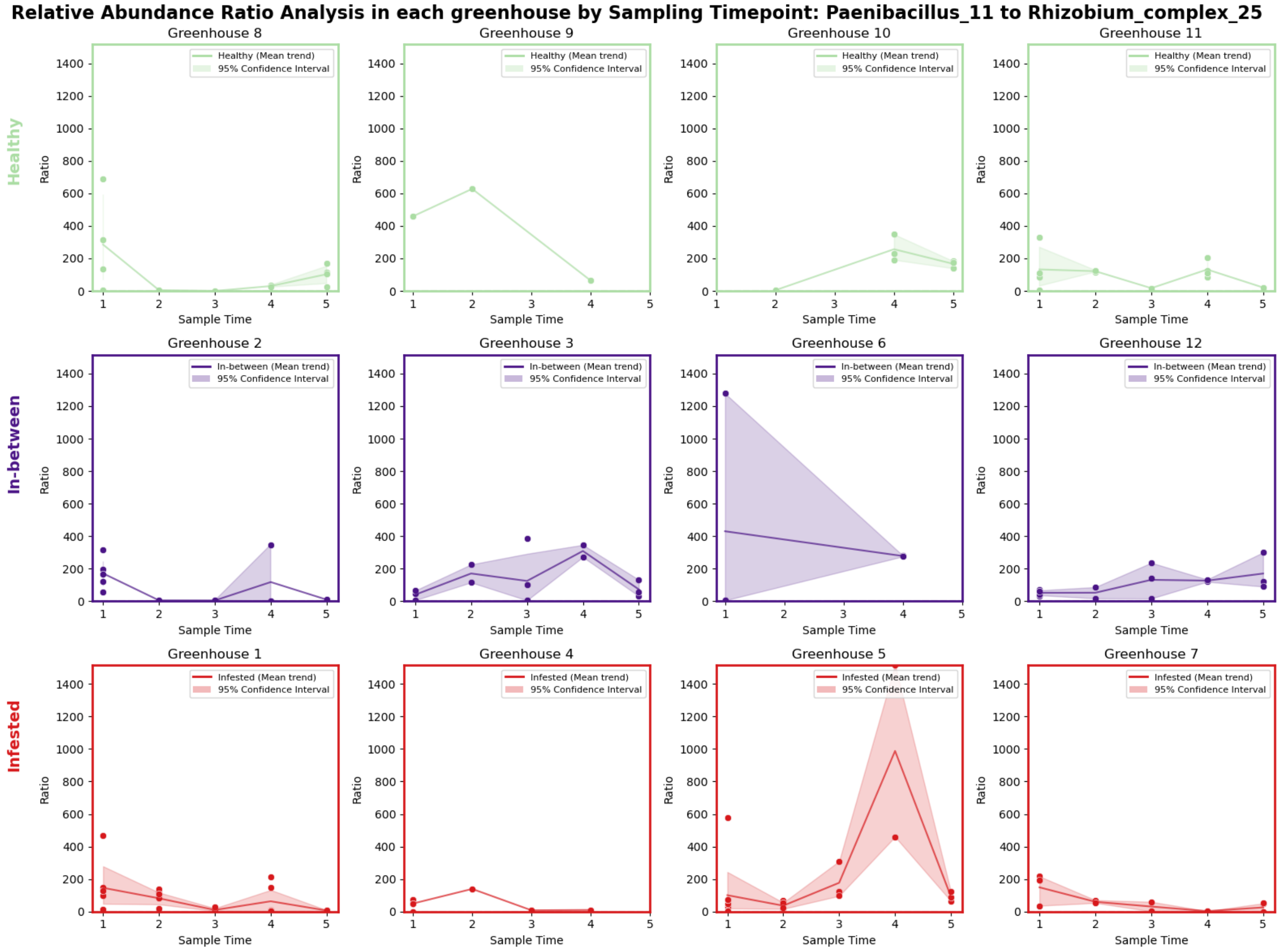
Relative Abundance Ratio Analysis in each greenhouse by Sampling Timepoint: Paenibacillus_11 to Rhizobium_complex_25
Greenhouse 8
Greenhouse 9
Greenhouse 10
Greenhouse 11
Greenhouse 2
Greenhouse 3
Greenhouse 6
Greenhouse 12
Greenhouse 1
Greenhouse 4
Greenhouse 5
Greenhouse 7
Healthy
In-between
Infested
Healthy (Mean trend)
In-between (Mean trend)
Infested (Mean trend)
95% Confidence Interval
Ratio
Sample Time
1
2
3
4
5
0
200
400
600
800
1000
1200
1400

Relative Abundance Ratio Analysis in each greenhouse by Sampling Timepoint: Paenibacillus_11 to Rhizobium_complex_29

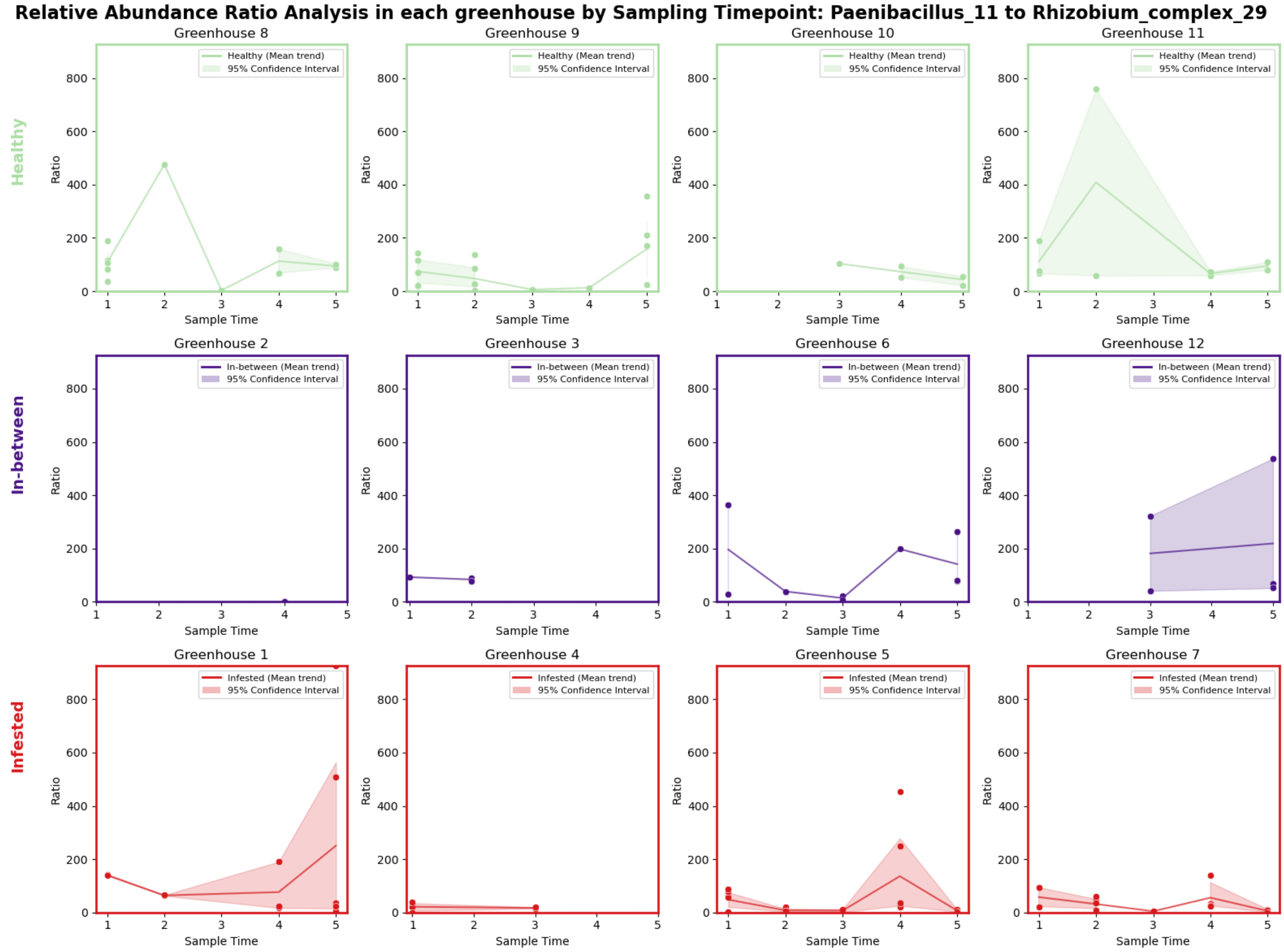

Relative Abundance Ratio Analysis in each greenhouse by stages: Paenibacillus_11 to Rhizobium_complex_29

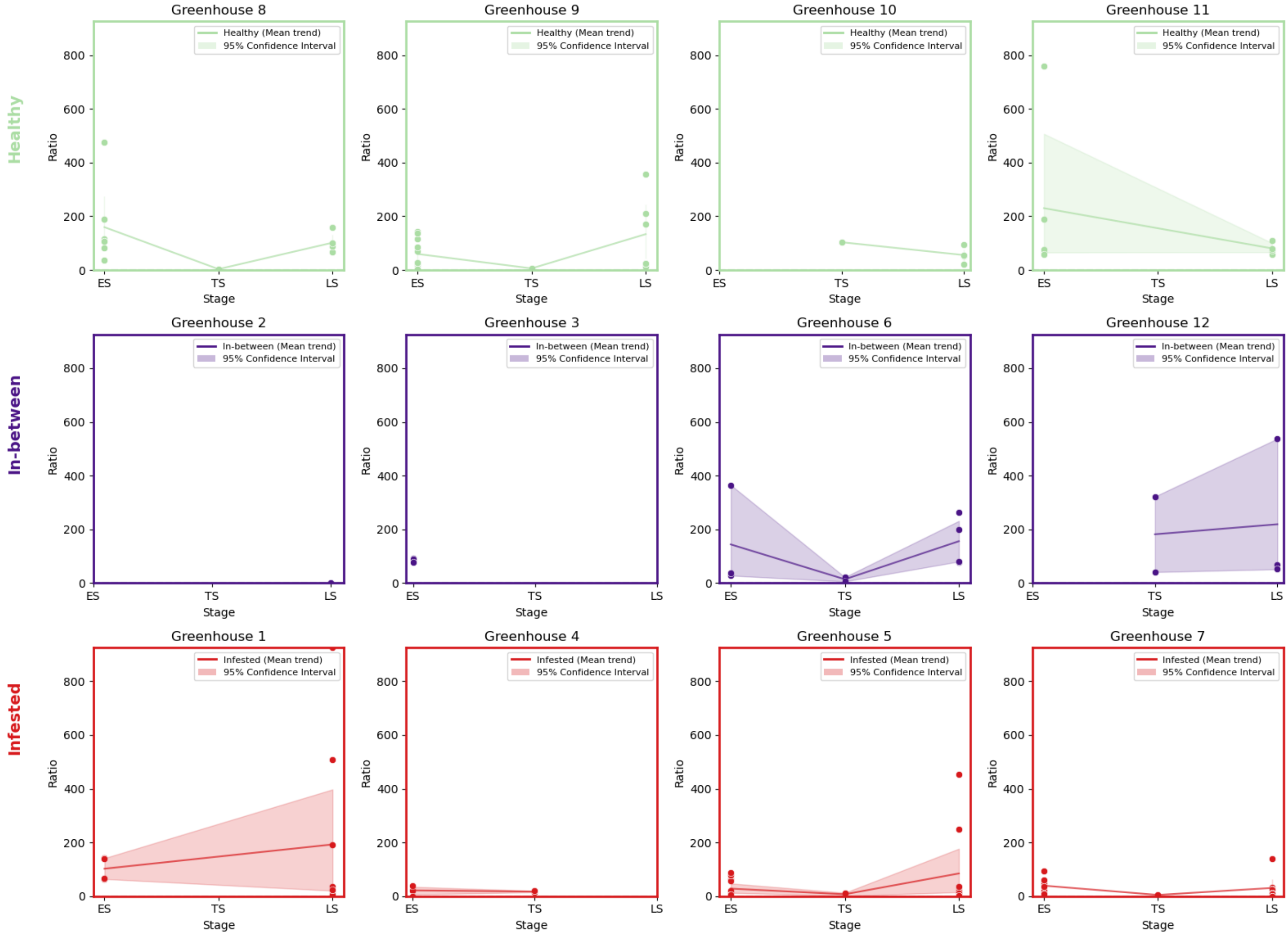

Relative Abundance Ratio Analysis in each greenhouse by Sampling Timepoint: Paenibacillus_15 to Rhizobium_complex_25

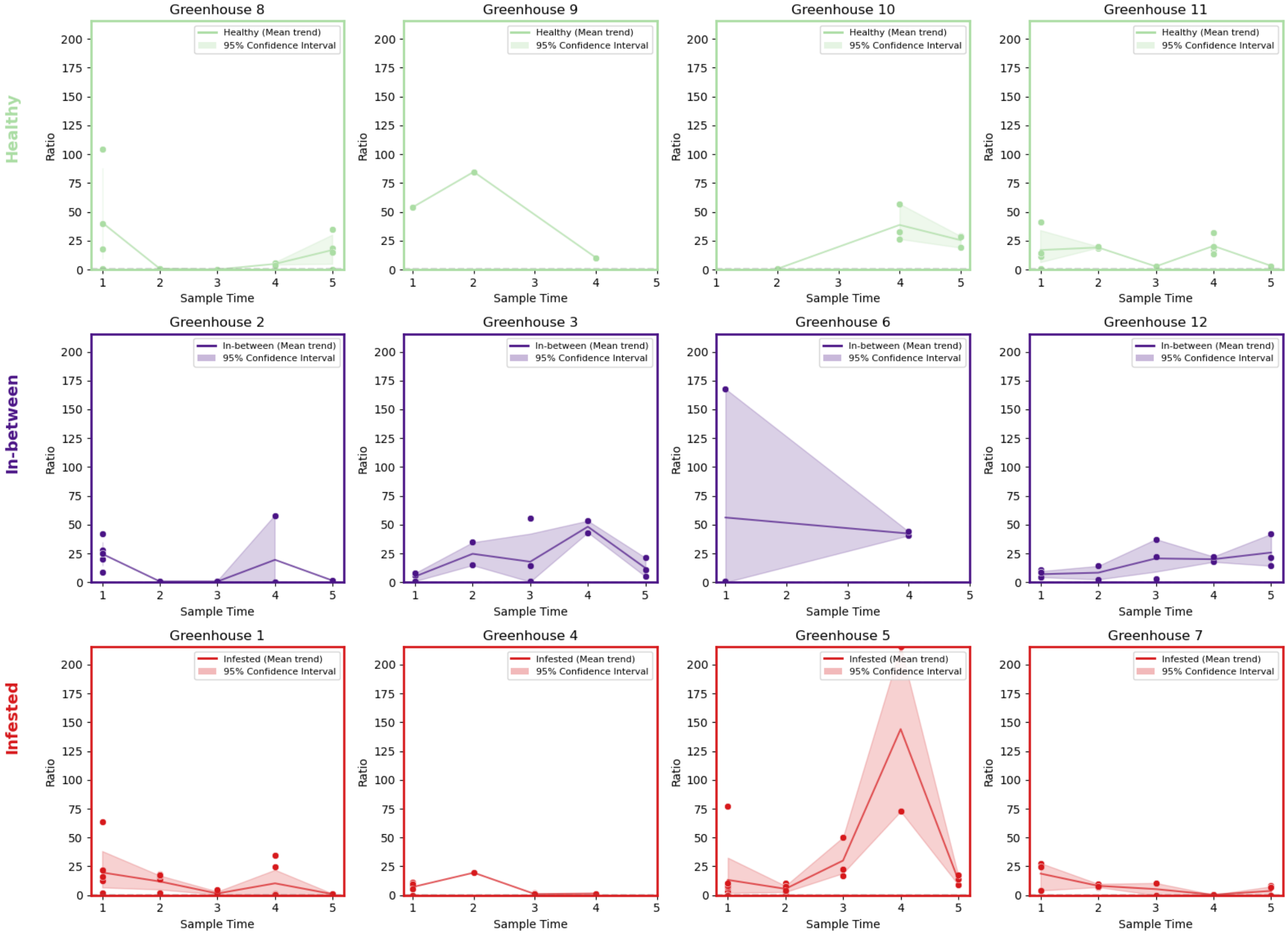

Relative Abundance Ratio Analysis in each greenhouse by stages: Paenibacillus_15 to Rhizobium_complex_25

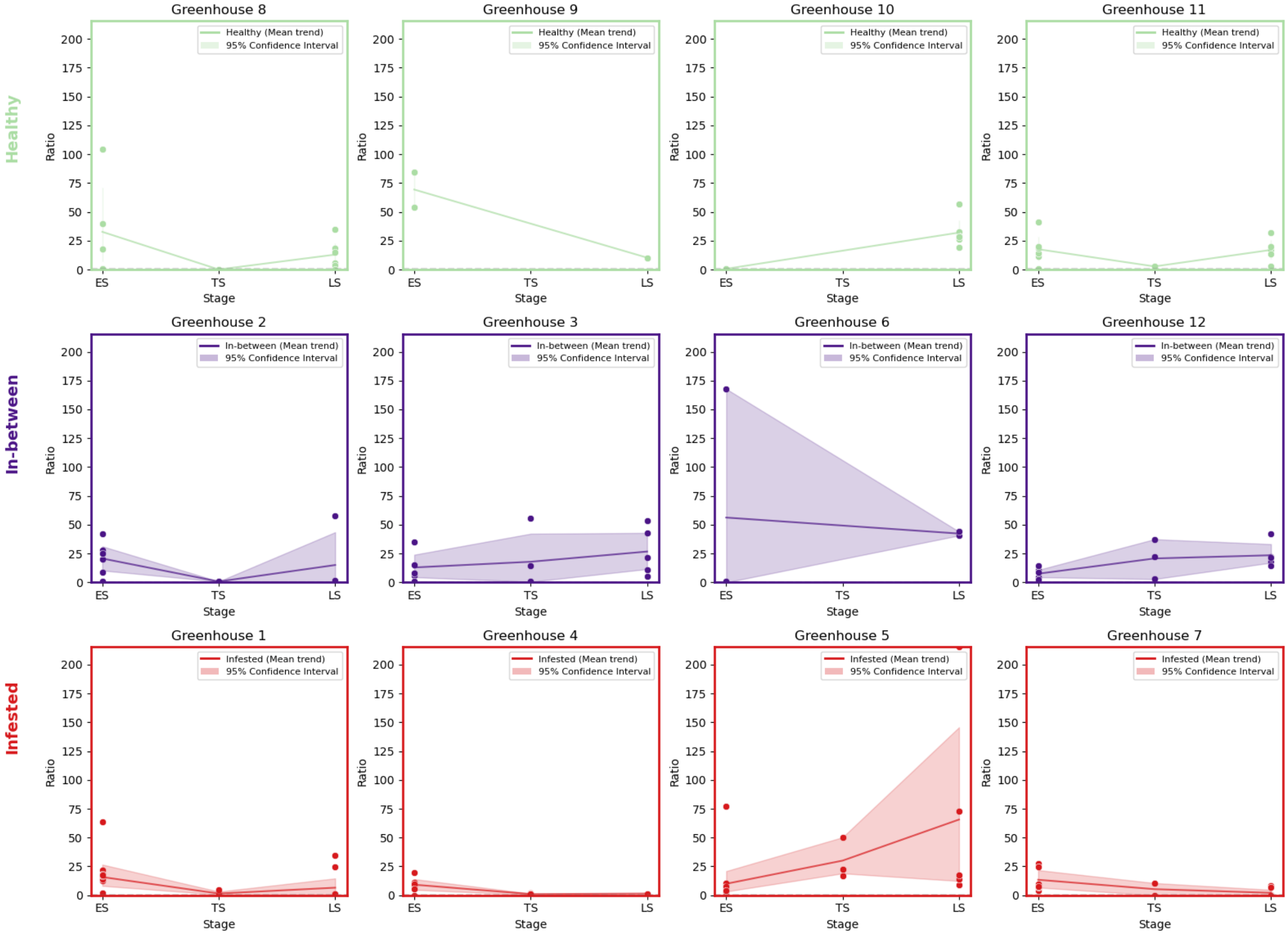

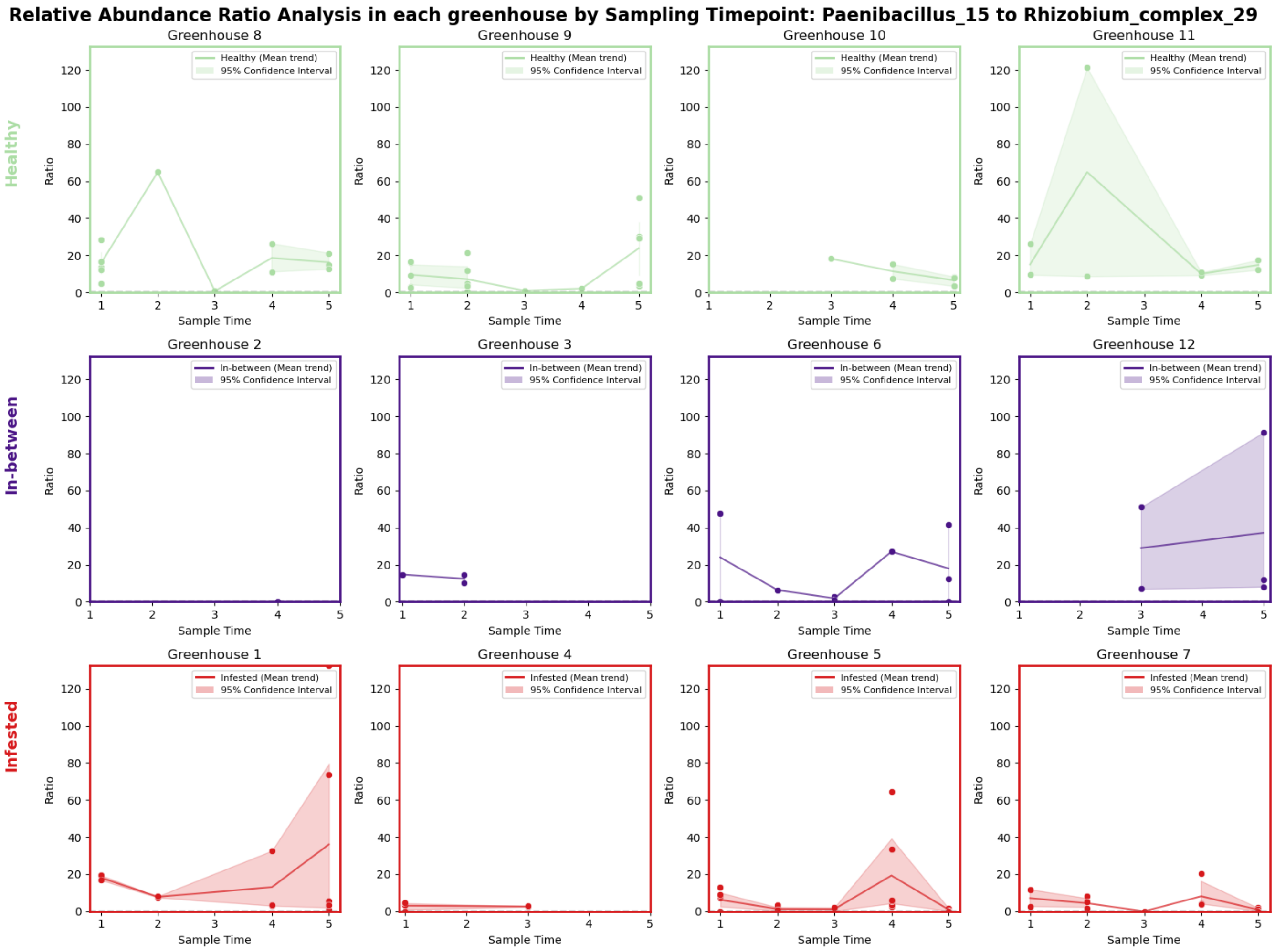
Relative Abundance Ratio Analysis in each greenhouse by Sampling Timepoint: Paenibacillus_15 to Rhizobium_complex_29
Greenhouse 8
Greenhouse 9
Greenhouse 10
Greenhouse 11
Greenhouse 2
Greenhouse 3
Greenhouse 6
Greenhouse 12
Greenhouse 1
Greenhouse 4
Greenhouse 5
Greenhouse 7
Healthy
In-between
Infested
Healthy (Mean trend)
In-between (Mean trend)
Infested (Mean trend)
95% Confidence Interval
Ratio
Sample Time

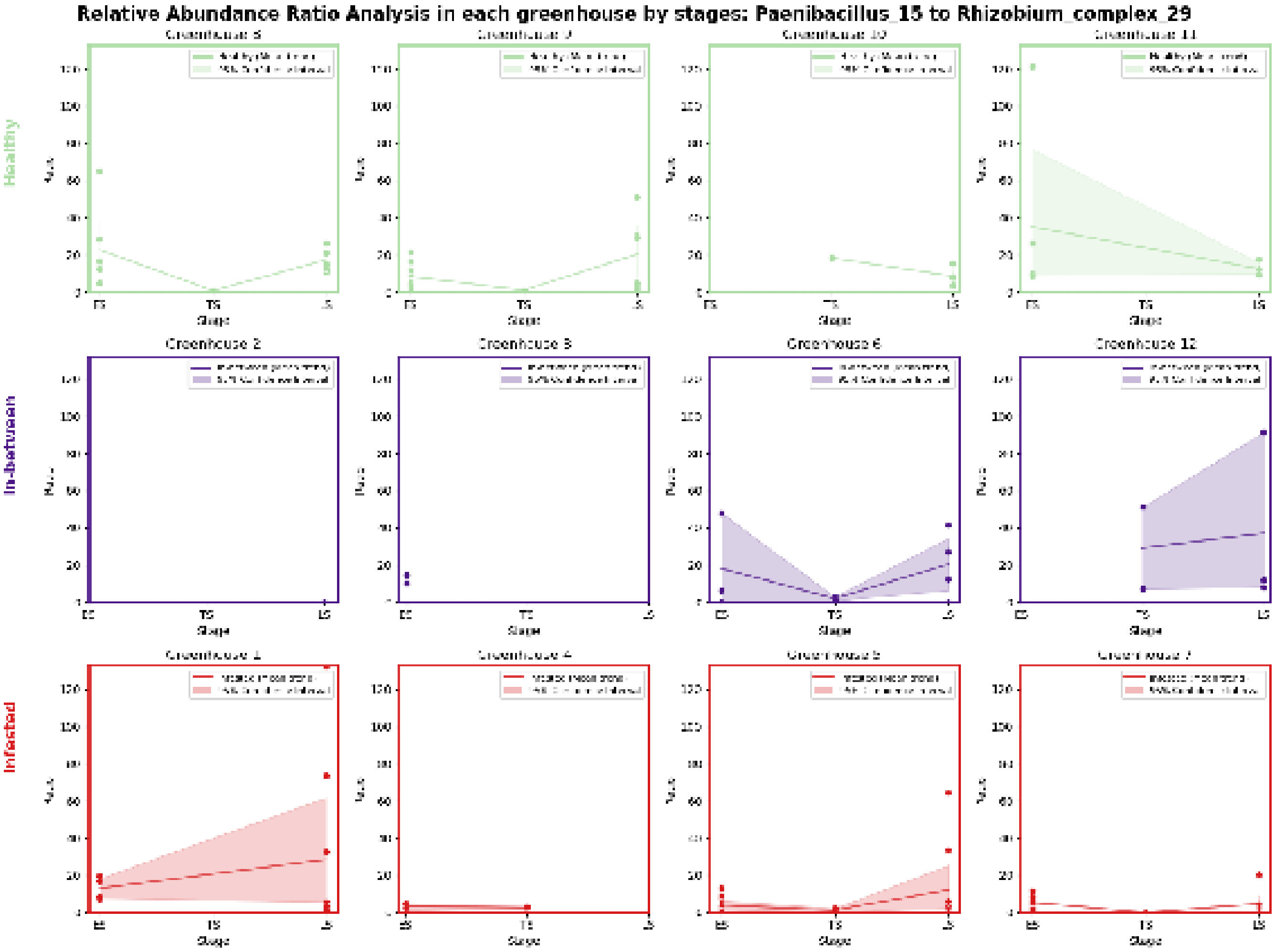
Relative Abundance Ratio Analysis in each greenhouse by stages: Paenibacillus_15 to Rhizobium_complex_29
Greenhouse 2
Greenhouse 3
Greenhouse 6
Greenhouse 12
Healthy
In-between
Infested

Relative Abundance Ratio Analysis in each greenhouse by Sampling Timepoint: Paenibacillus_16 to Rhizobium_complex_25

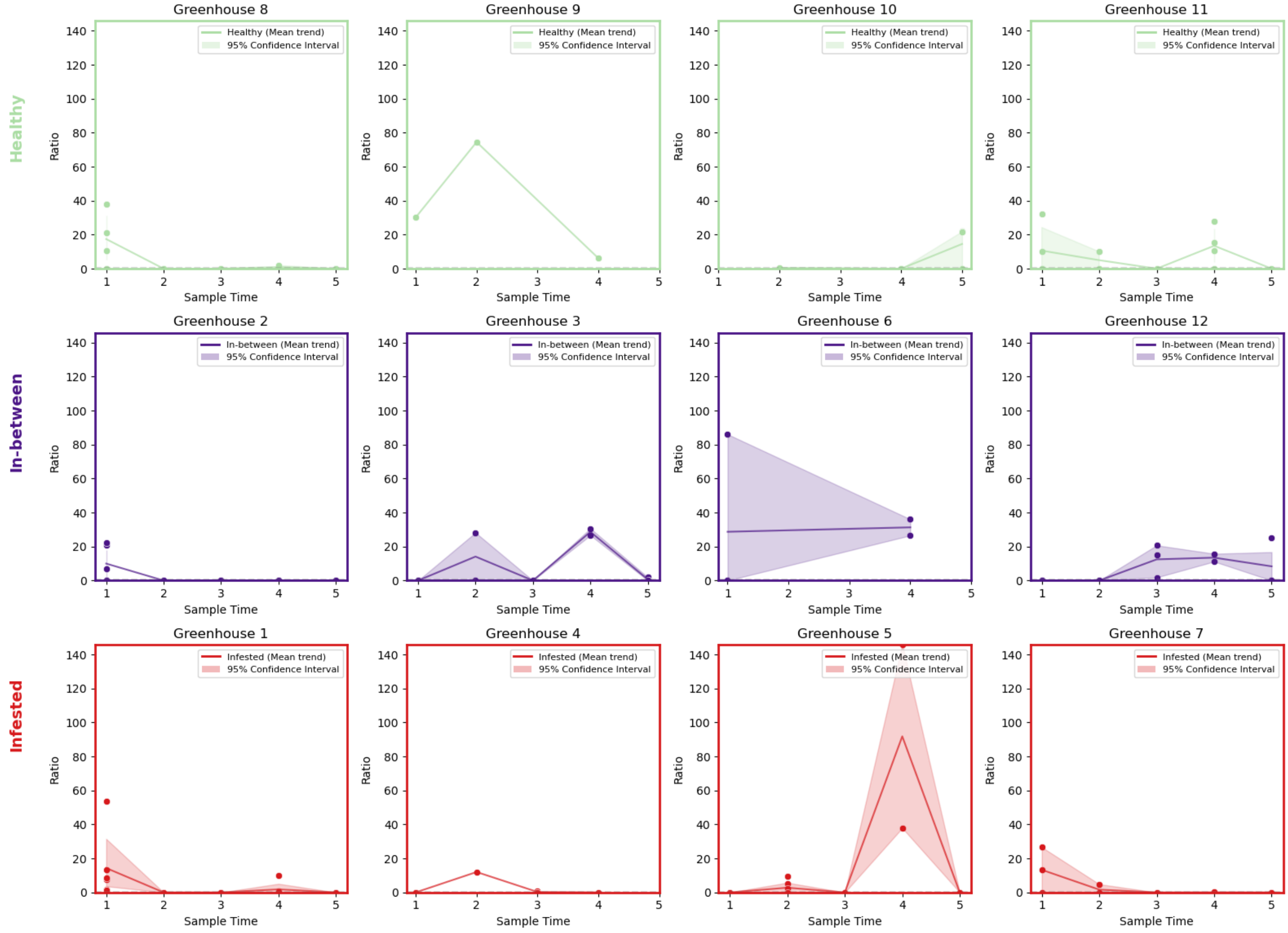

Relative Abundance Ratio Analysis in each greenhouse by stages: Paenibacillus_16 to Rhizobium_complex_25

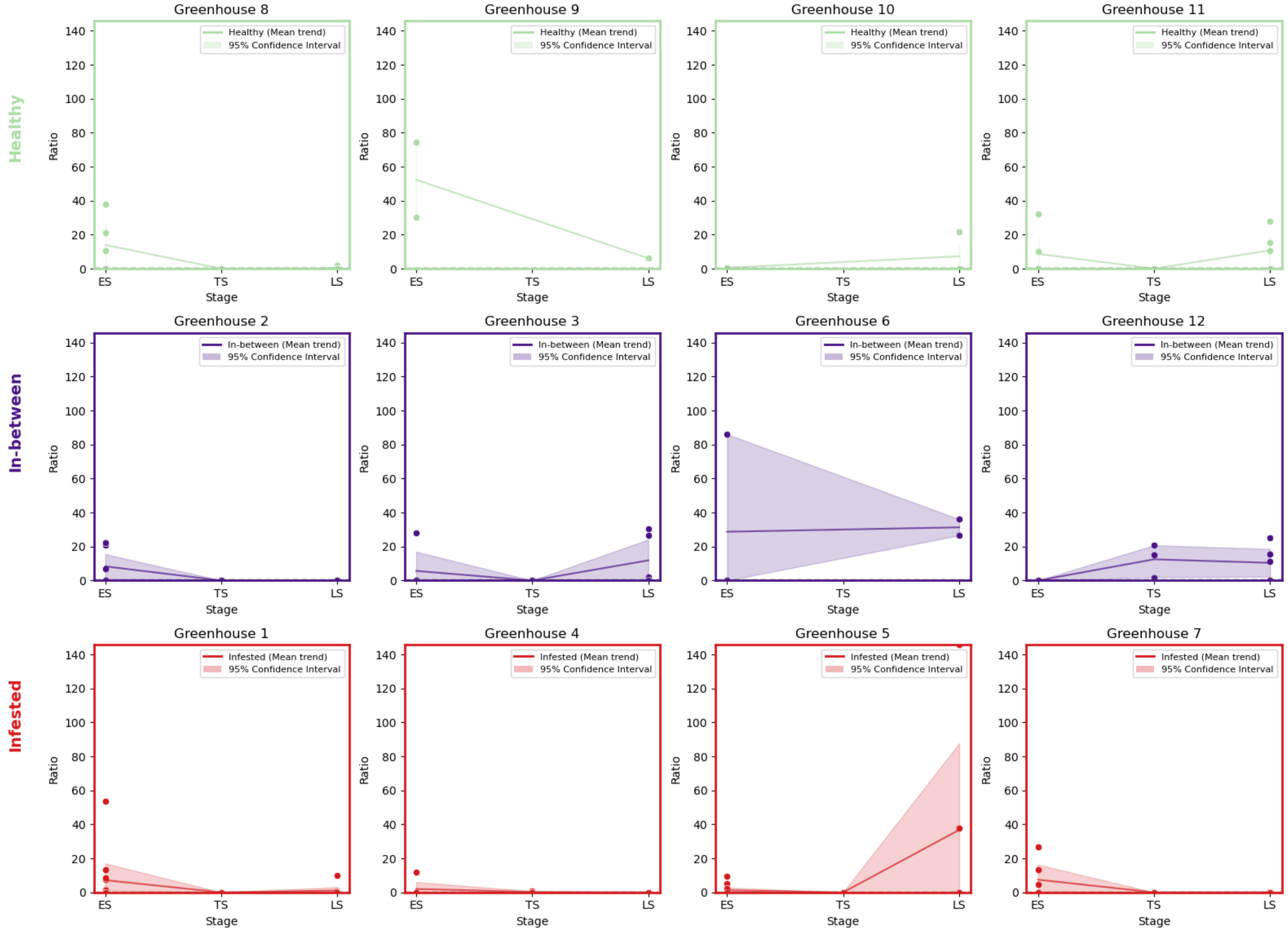

Relative Abundance Ratio Analysis in each greenhouse by Sampling Timepoint: Paenibacillus_16 to Rhizobium_complex_29

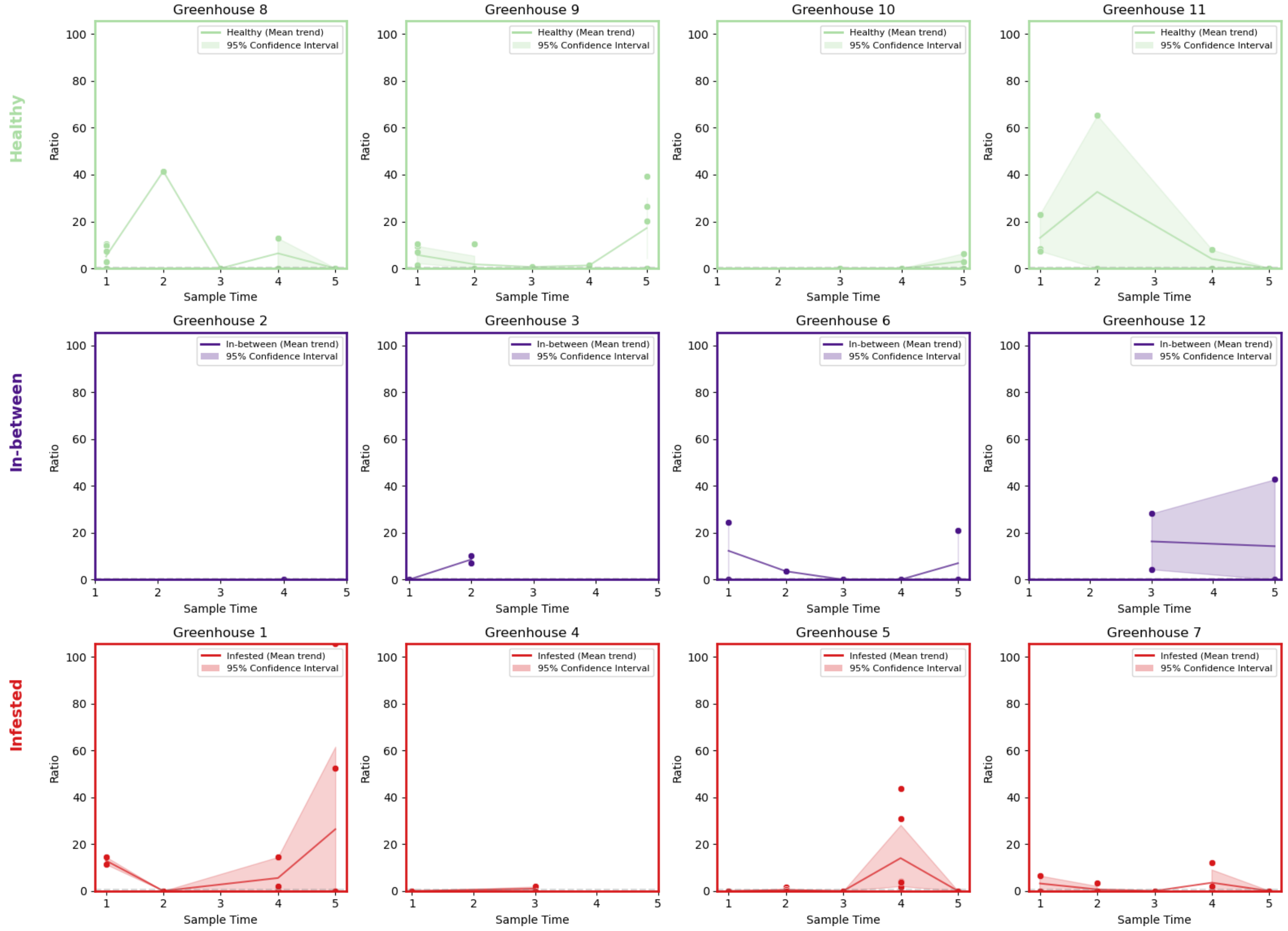

Relative Abundance Ratio Analysis in each greenhouse by stages: Paenibacillus_16 to Rhizobium_complex_29

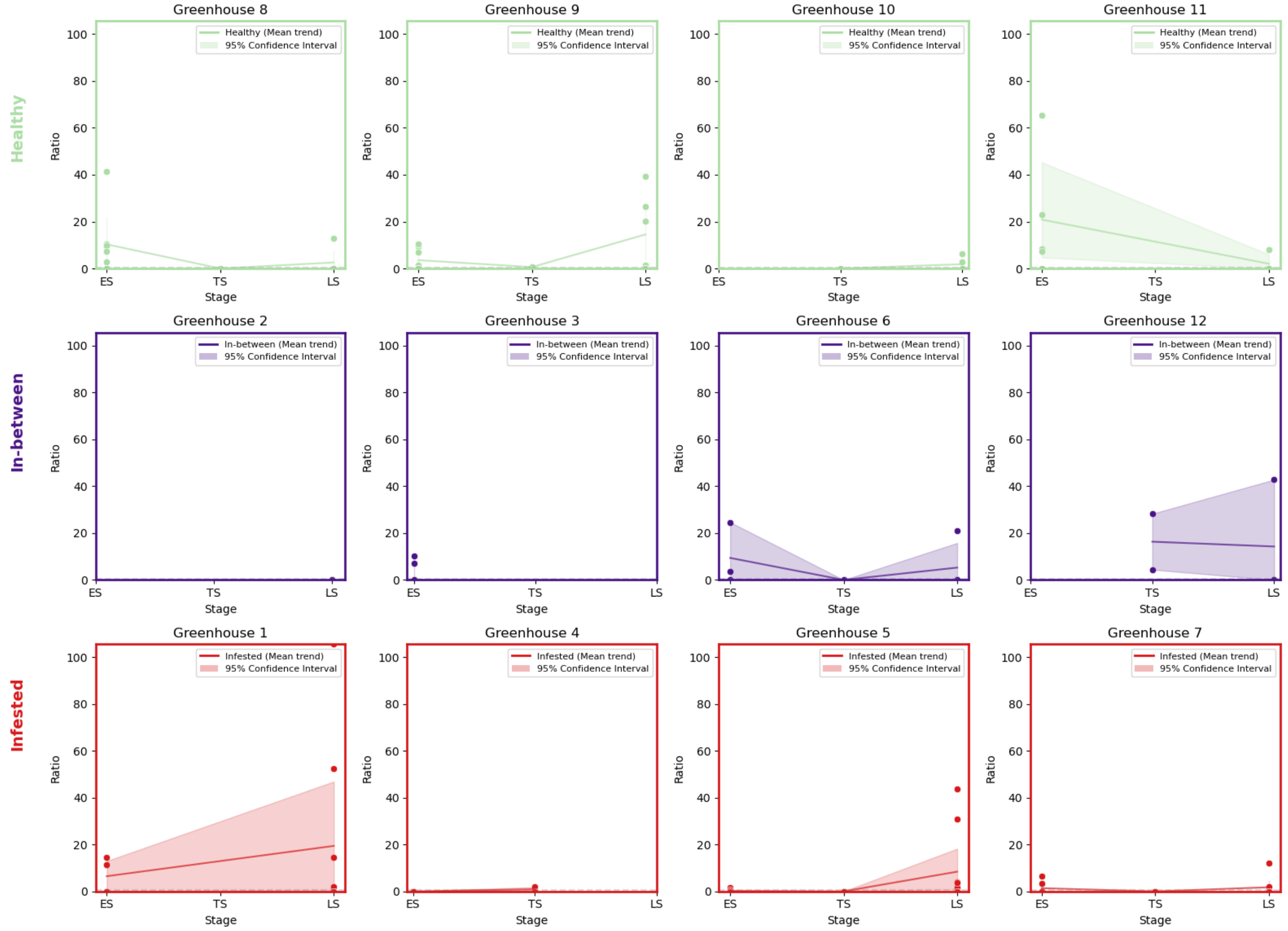

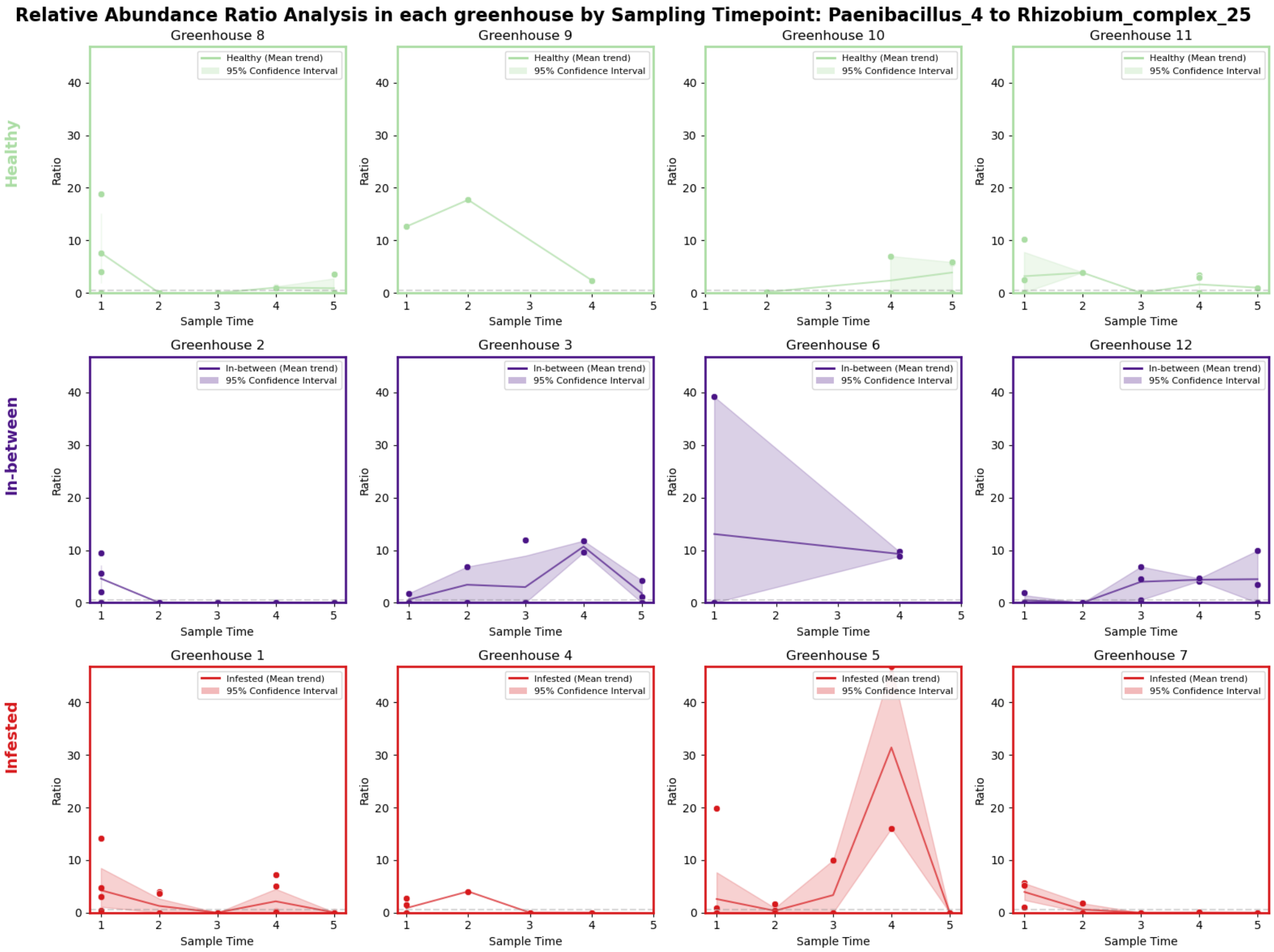
Relative Abundance Ratio Analysis in each greenhouse by Sampling Timepoint: Paenibacillus_4 to Rhizobium_complex_25
Healthy
In-between
Infested
Greenhouse 8
Greenhouse 9
Greenhouse 10
Greenhouse 11
Greenhouse 2
Greenhouse 3
Greenhouse 6
Greenhouse 12
Greenhouse 1
Greenhouse 4
Greenhouse 5
Greenhouse 7
Healthy (Mean trend)
In-between (Mean trend)
Infested (Mean trend)
95% Confidence Interval
Ratio
Sample Time

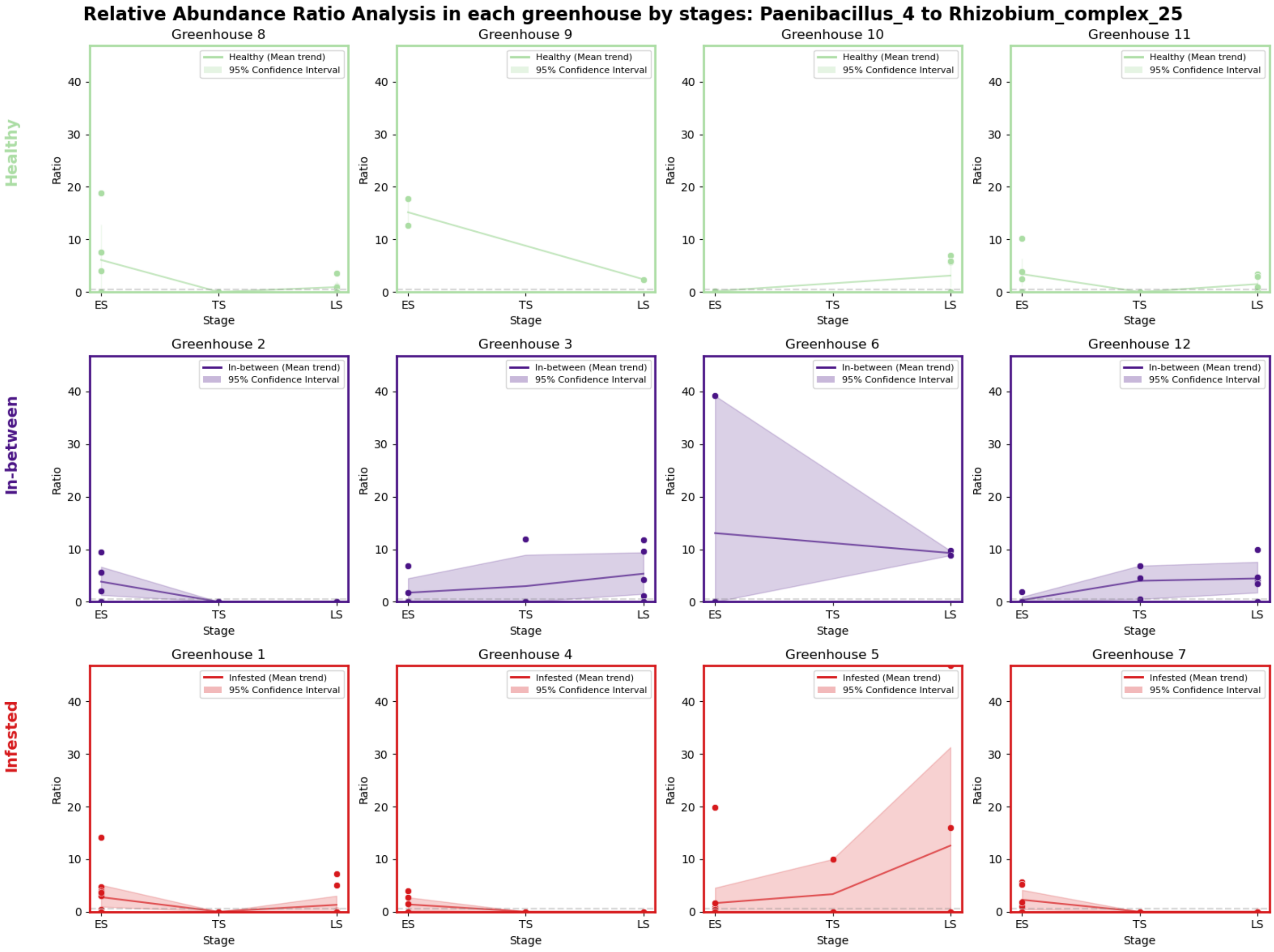
Relative Abundance Ratio Analysis in each greenhouse by stages: Paenibacillus_4 to Rhizobium_complex_25
Healthy
In-between
Infested
Greenhouse 8
Greenhouse 9
Greenhouse 10
Greenhouse 11
Greenhouse 2
Greenhouse 3
Greenhouse 6
Greenhouse 12
Greenhouse 1
Greenhouse 4
Greenhouse 5
Greenhouse 7
Healthy (Mean trend)
In-between (Mean trend)
Infested (Mean trend)
95% Confidence Interval
Ratio
Stage
ES
TS
LS

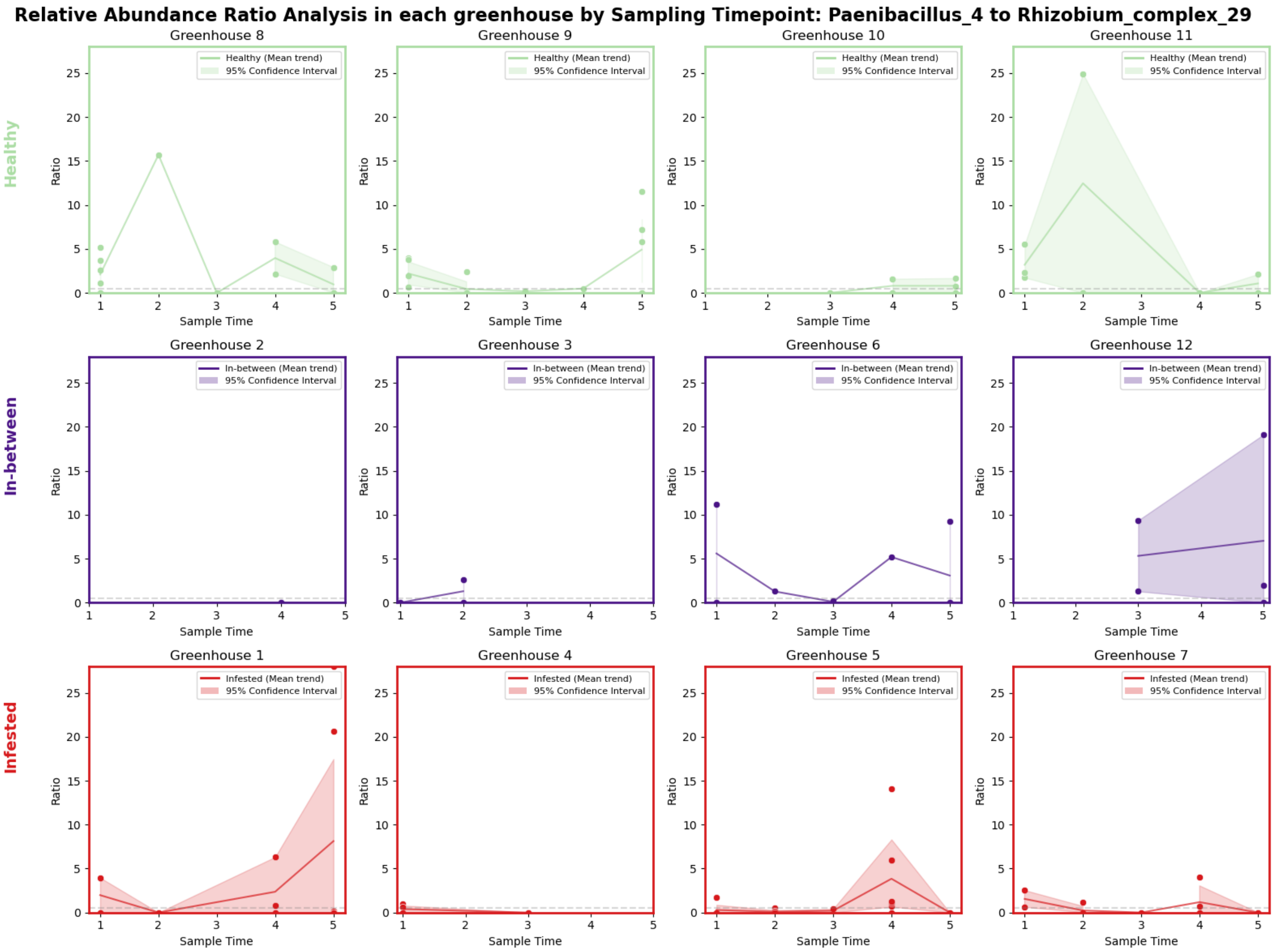
Relative Abundance Ratio Analysis in each greenhouse by Sampling Timepoint: Paenibacillus_4 to Rhizobium_complex_29
Healthy
In-between
Infested
Greenhouse 8
Greenhouse 9
Greenhouse 10
Greenhouse 11
Greenhouse 2
Greenhouse 3
Greenhouse 6
Greenhouse 12
Greenhouse 1
Greenhouse 4
Greenhouse 5
Greenhouse 7
Healthy (Mean trend)
In-between (Mean trend)
Infested (Mean trend)
95% Confidence Interval
Ratio
Sample Time

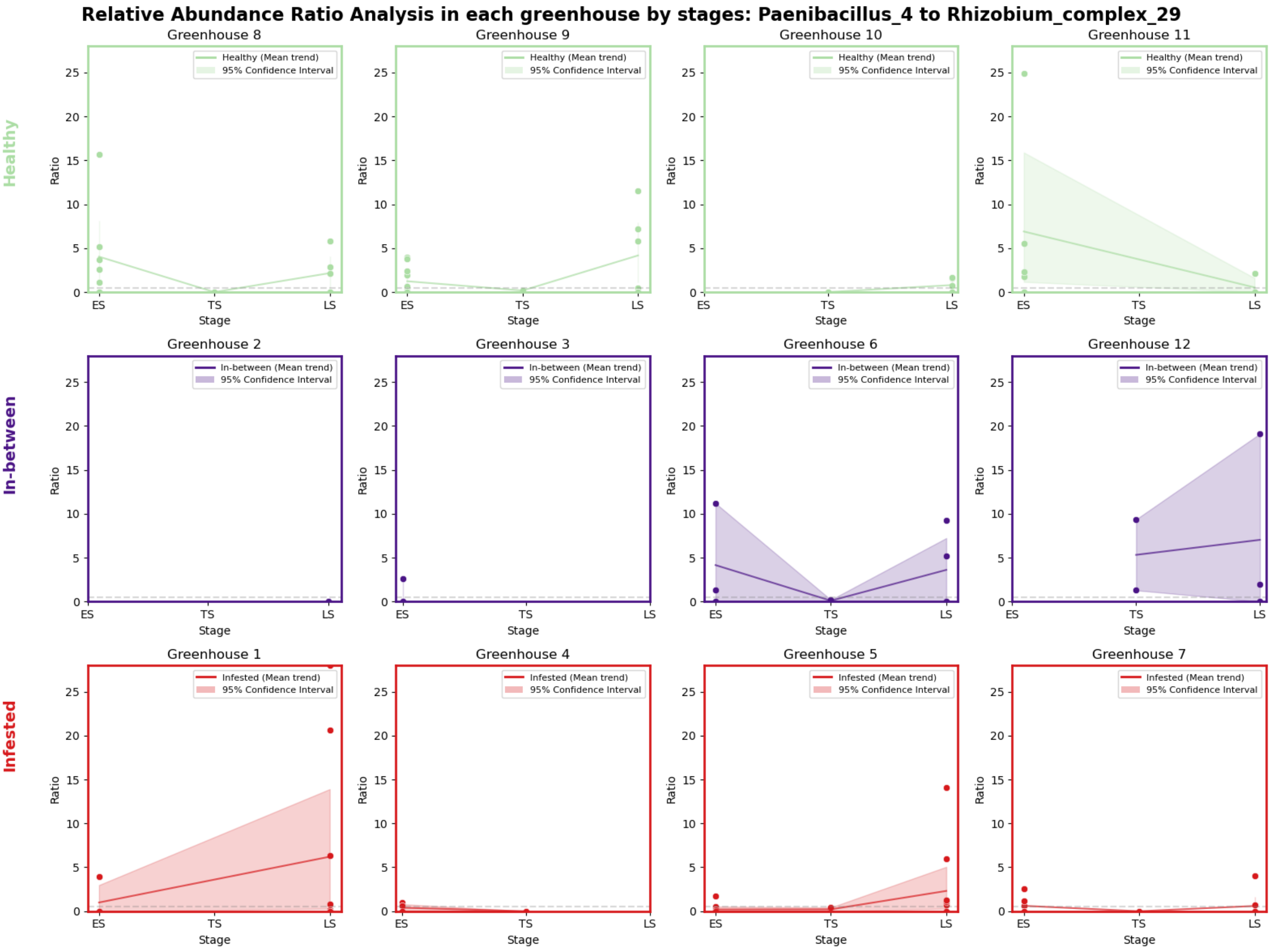
Relative Abundance Ratio Analysis in each greenhouse by stages: Paenibacillus_4 to Rhizobium_complex_29
Healthy
In-between
Infested
Greenhouse 8
Greenhouse 9
Greenhouse 10
Greenhouse 11
Greenhouse 2
Greenhouse 3
Greenhouse 6
Greenhouse 12
Greenhouse 1
Greenhouse 4
Greenhouse 5
Greenhouse 7
Healthy (Mean trend)
In-between (Mean trend)
Infested (Mean trend)
95% Confidence Interval
Ratio
Stage
ES
TS
LS

Modeled Relative Abundance Ratio Analysis in each greenhouse by Sampling Timepoint: Paenibacillus_11 to Rhizobium_complex_25

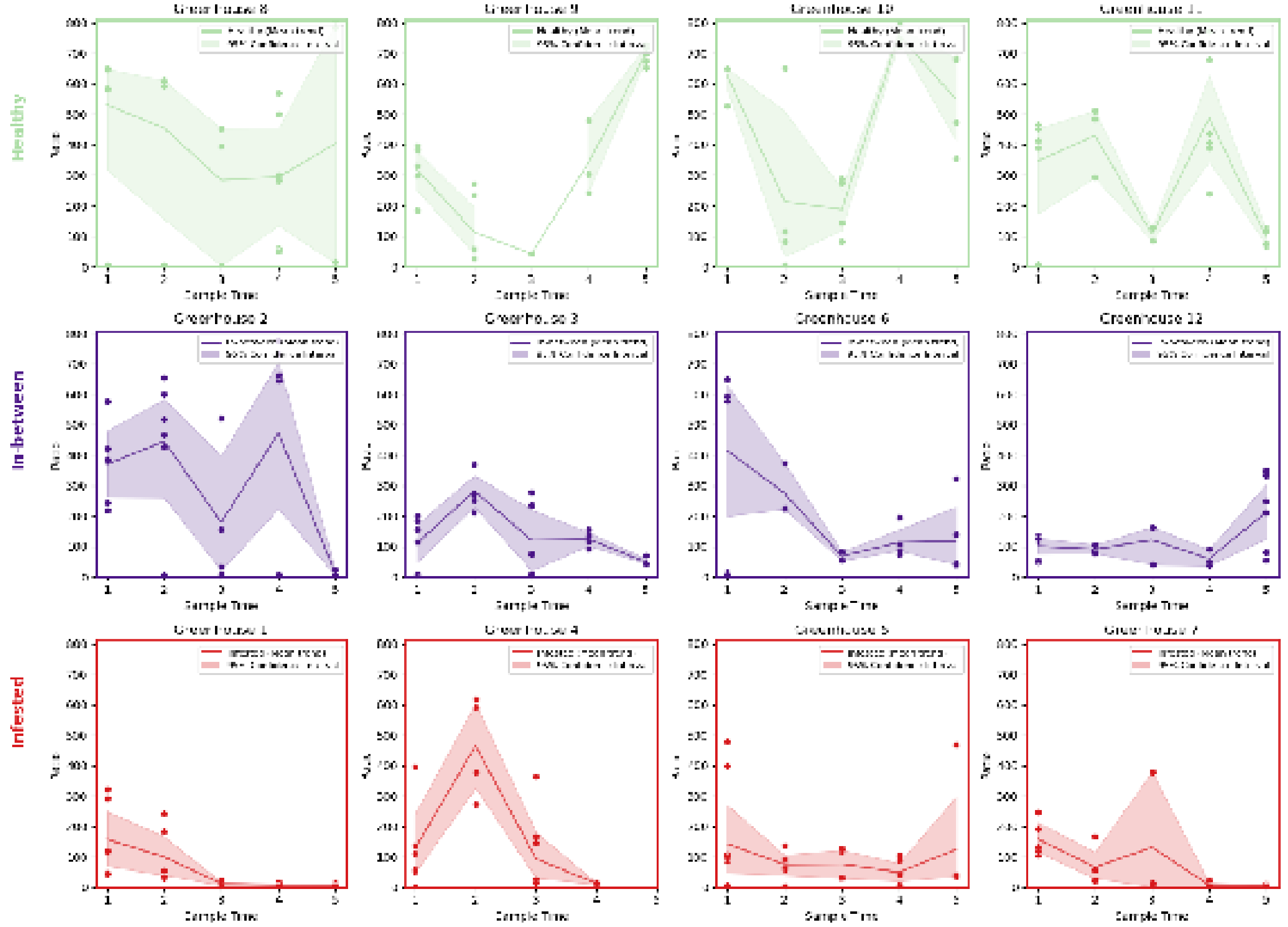

Modeled Relative Abundance Ratio Analysis in each greenhouse by stages: Paenibacillus_11 to Rhizobium_complex_25

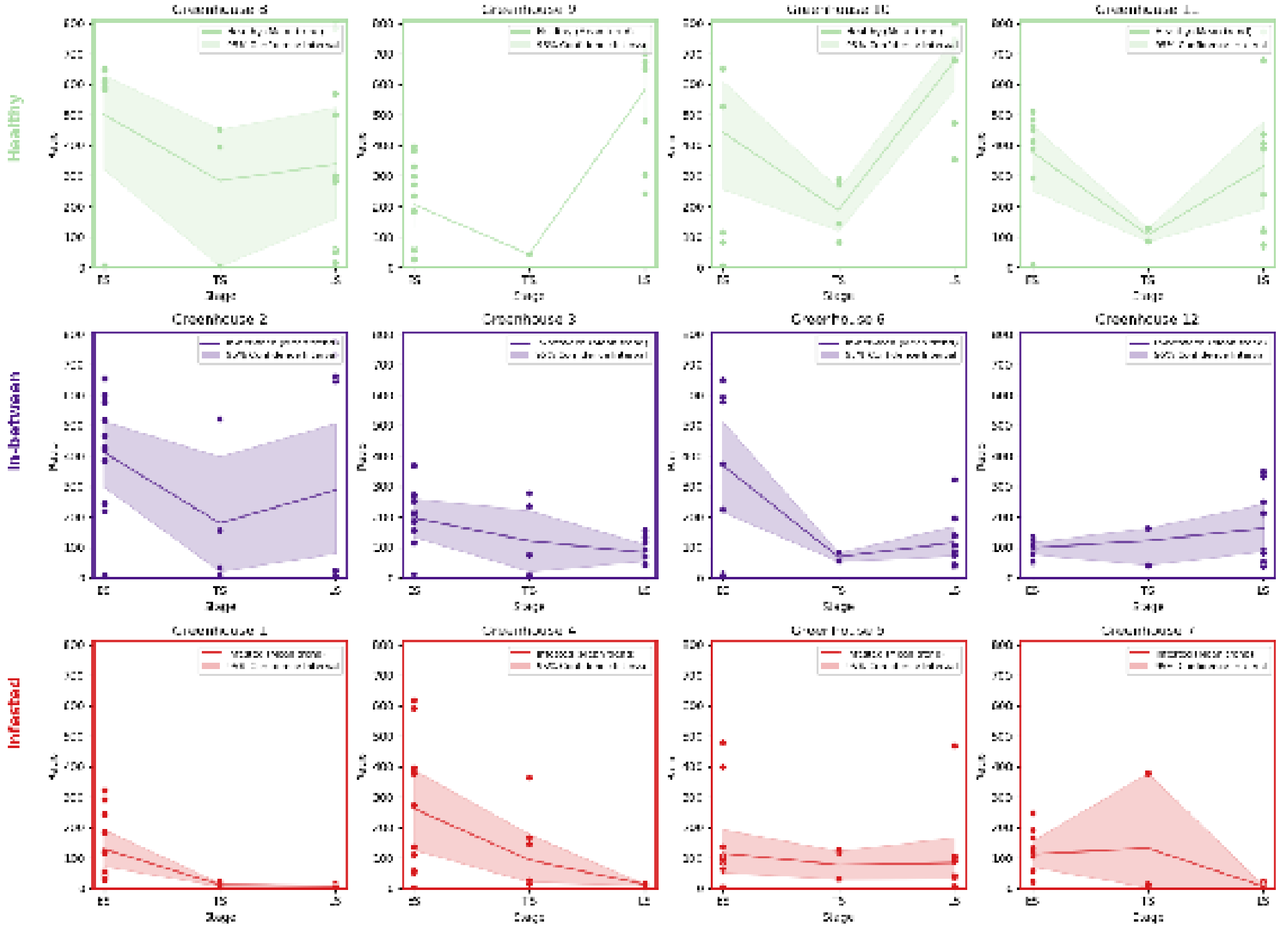

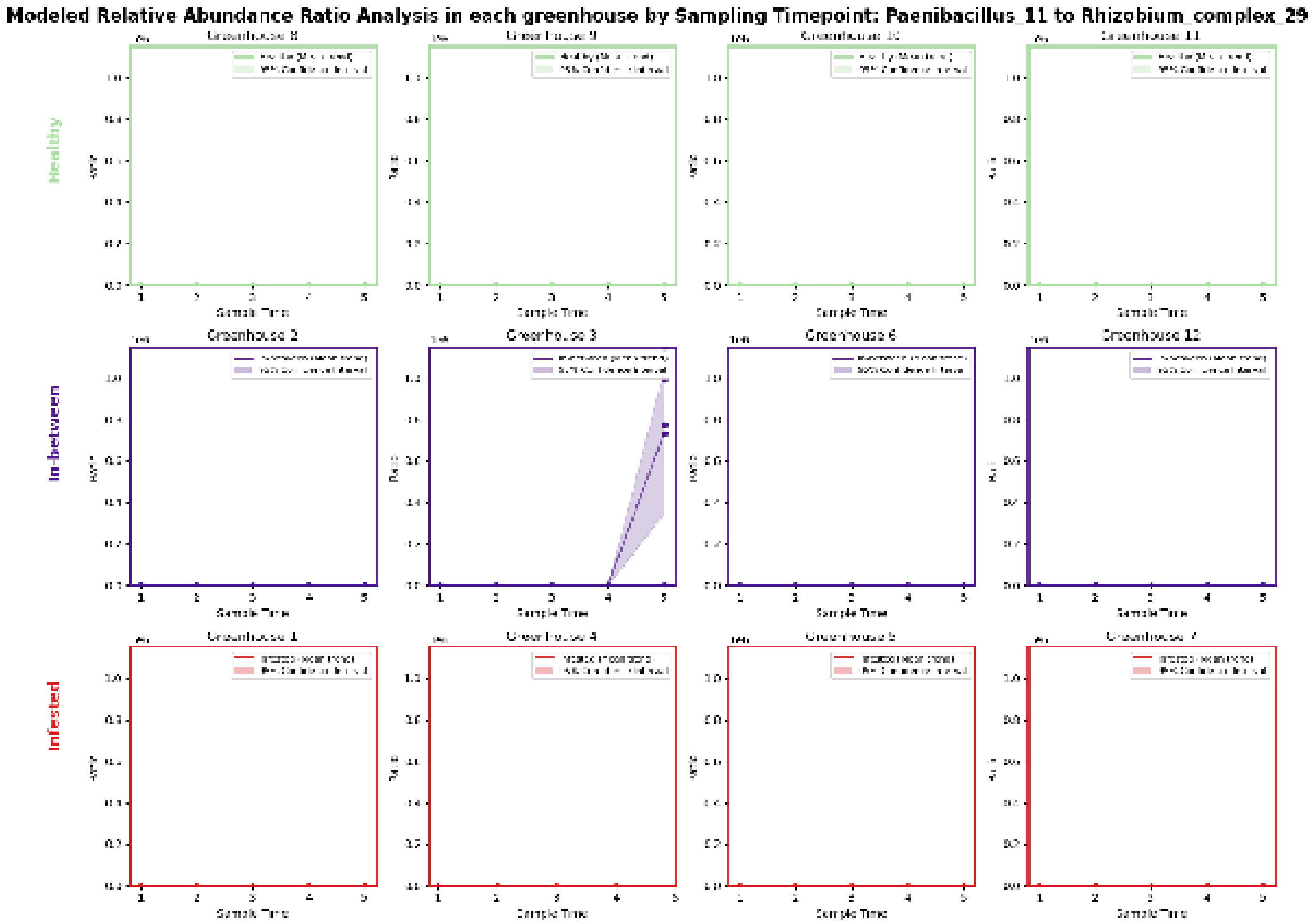
Modeled Relative Abundance Ratio Analysis in each greenhouse by Sampling Timepoint: Paenibacillus_11 to Rhizobium_complex_29
Healthy
In-between
Infested
Greenhouse 2
Greenhouse 3
Greenhouse 6
Greenhouse 12
Greenhouse 1
Greenhouse 4
Greenhouse 5
Greenhouse 7
Sample Time

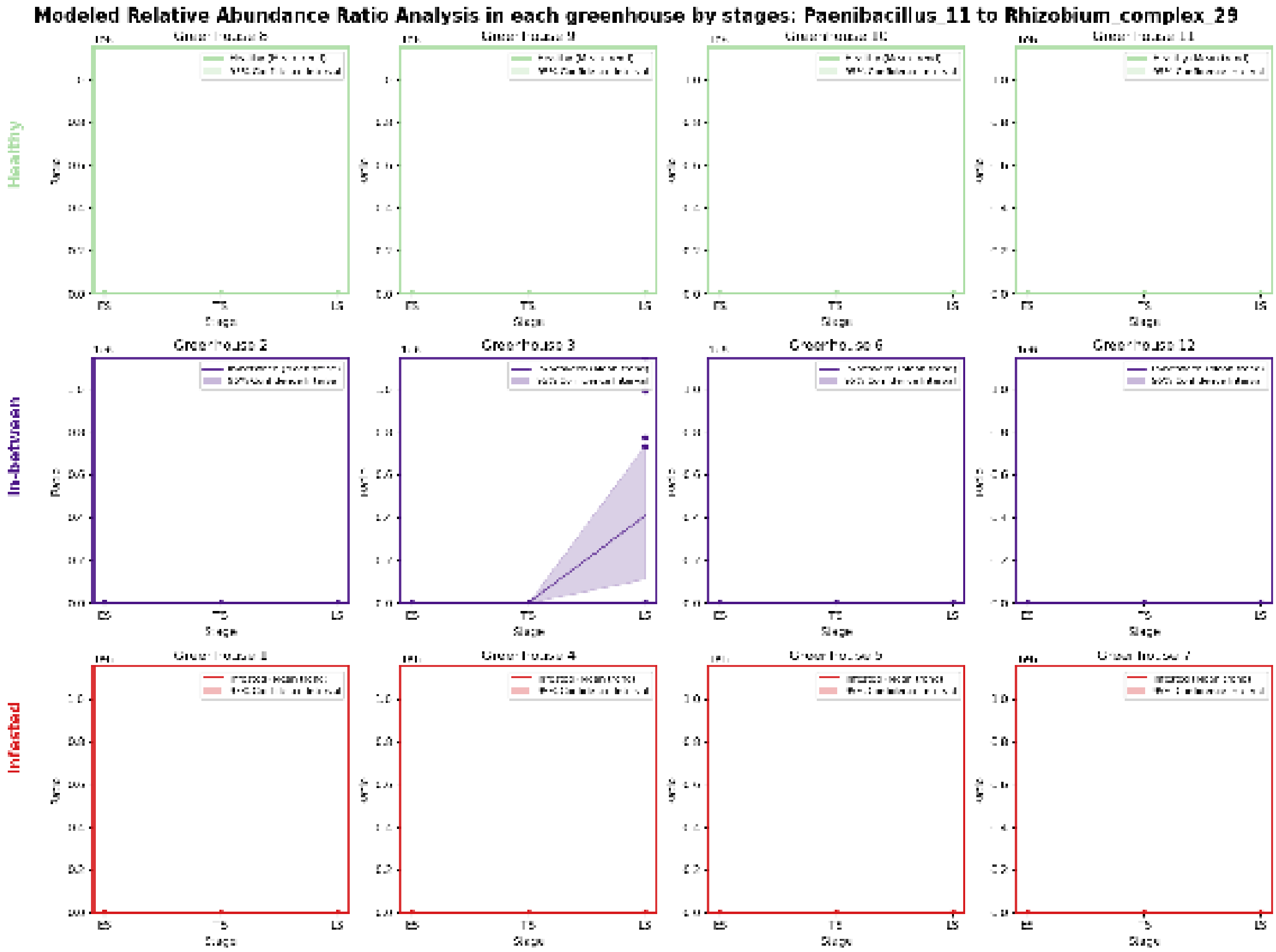
Modeled Relative Abundance Ratio Analysis in each greenhouse by stages: Paenibacillus_11 to Rhizobium_complex_29
Healthy
In-between
Infested
Greenhouse 2
Greenhouse 3
Greenhouse 6
Greenhouse 12
Stage

Modeled Relative Abundance Ratio Analysis in each greenhouse by Sampling Timepoint: Paenibacillus_15 to Rhizobium_complex_25

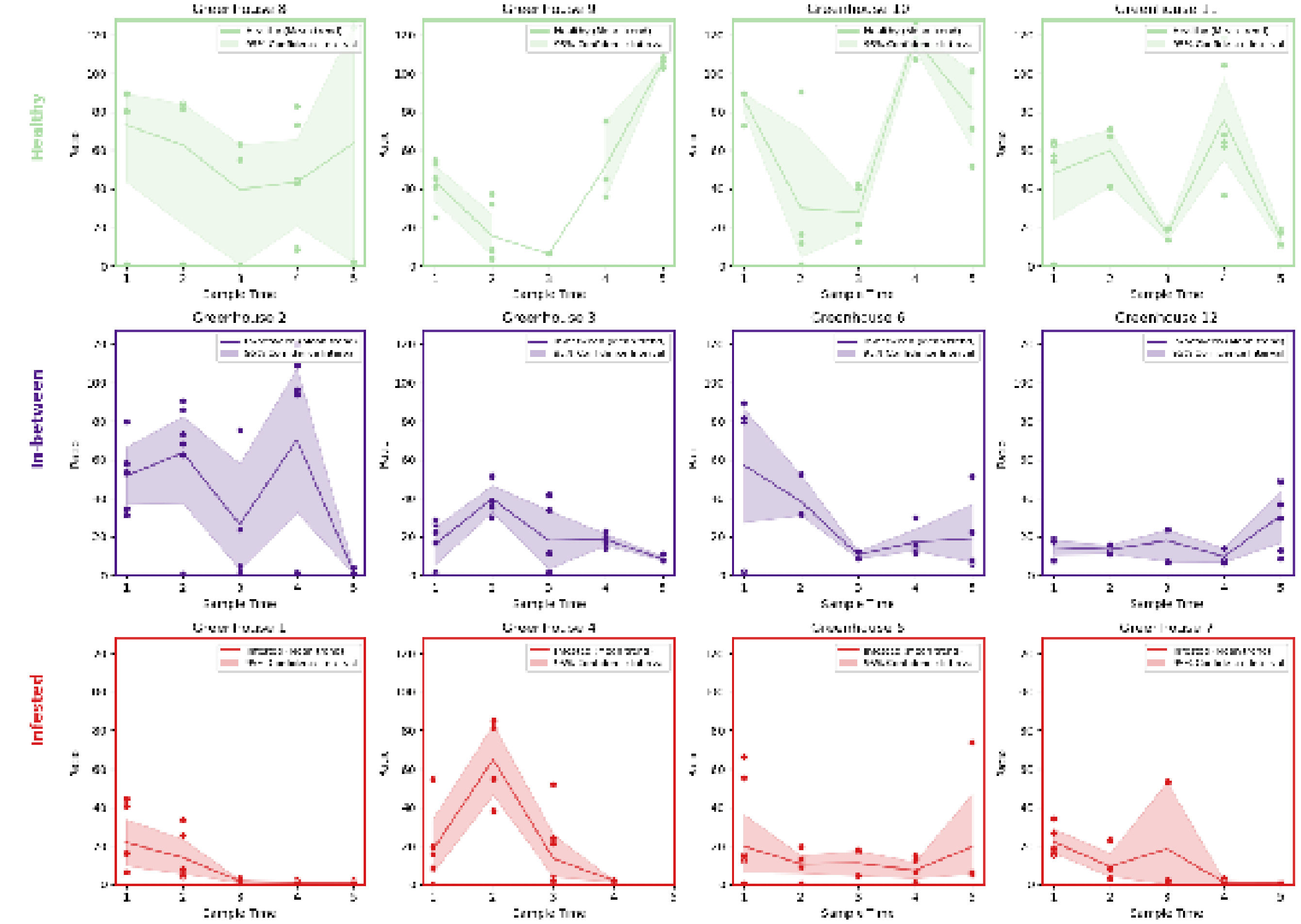

Modeled Relative Abundance Ratio Analysis in each greenhouse by stages: Paenibacillus_15 to Rhizobium_complex_25

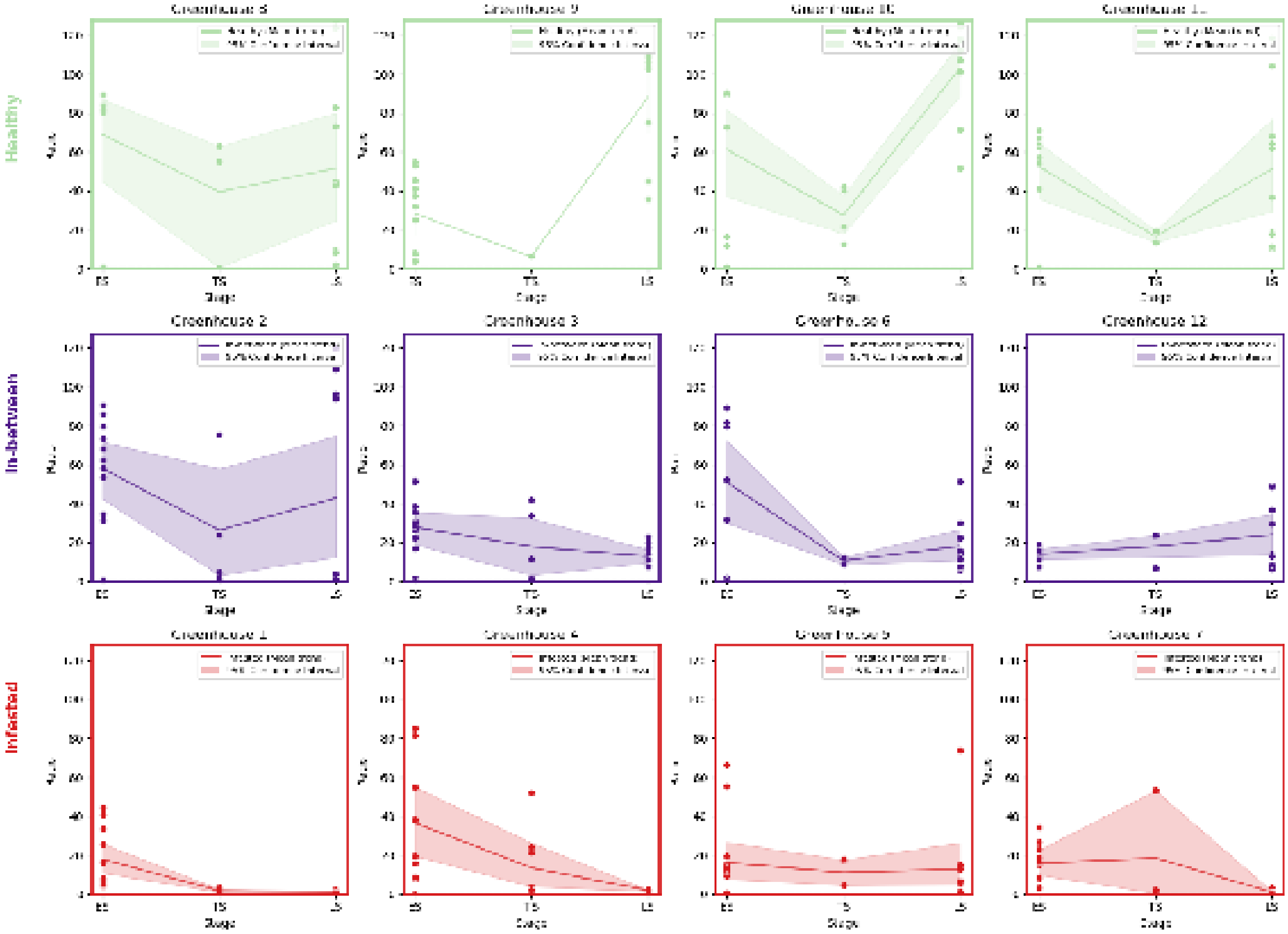

Modeled Relative Abundance Ratio Analysis in each greenhouse by Sampling Timepoint: Paenibacillus_15 to Rhizobium_complex_29

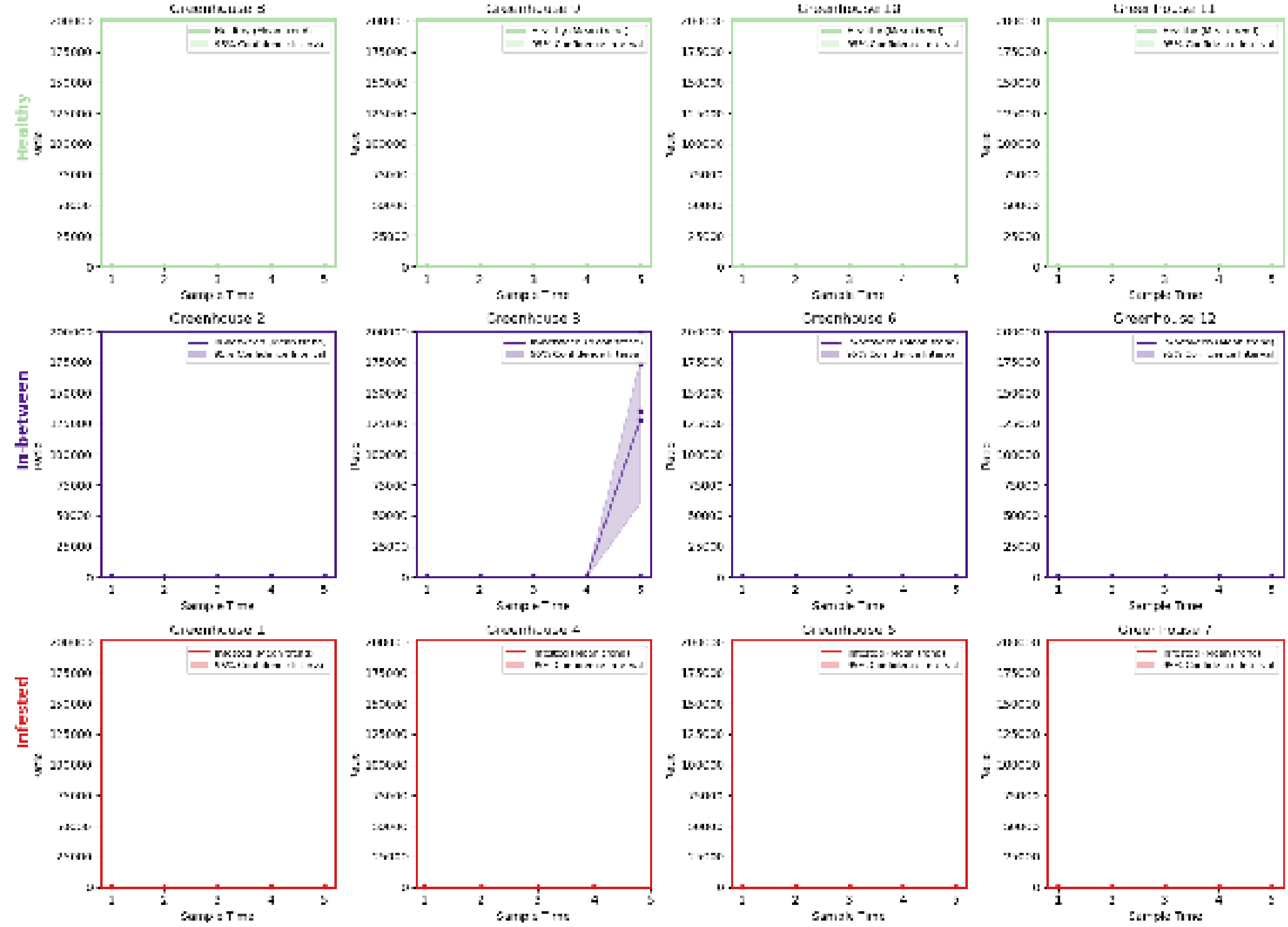

Modeled Relative Abundance Ratio Analysis in each greenhouse by stages: Paenibacillus_15 to Rhizobium_complex_29

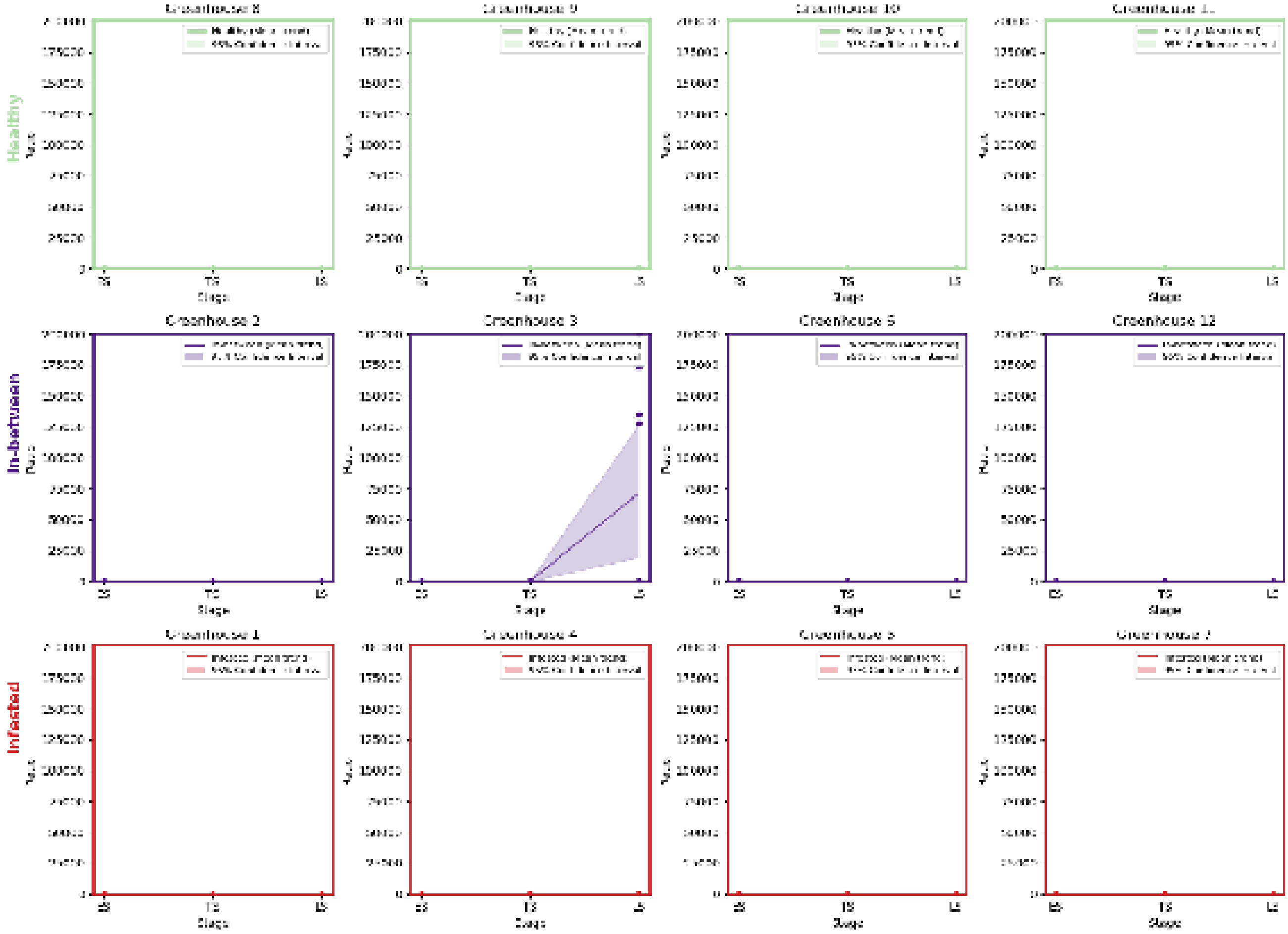

Modeled Relative Abundance Ratio Analysis in each greenhouse by Sampling Timepoint: Paenibacillus_16 to Rhizobium_complex_25

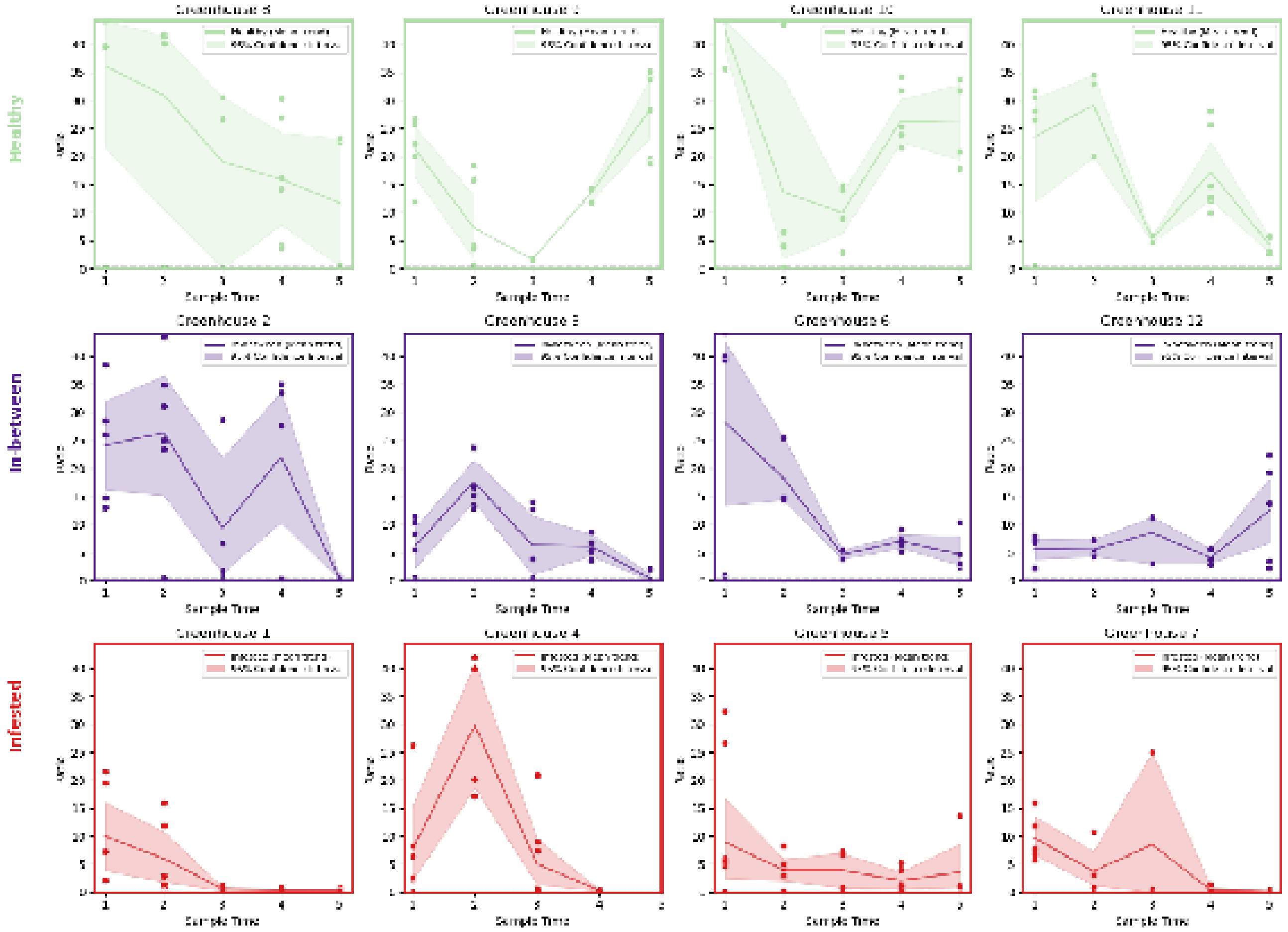

Modeled Relative Abundance Ratio Analysis in each greenhouse by stages: Paenibacillus_16 to Rhizobium_complex_25

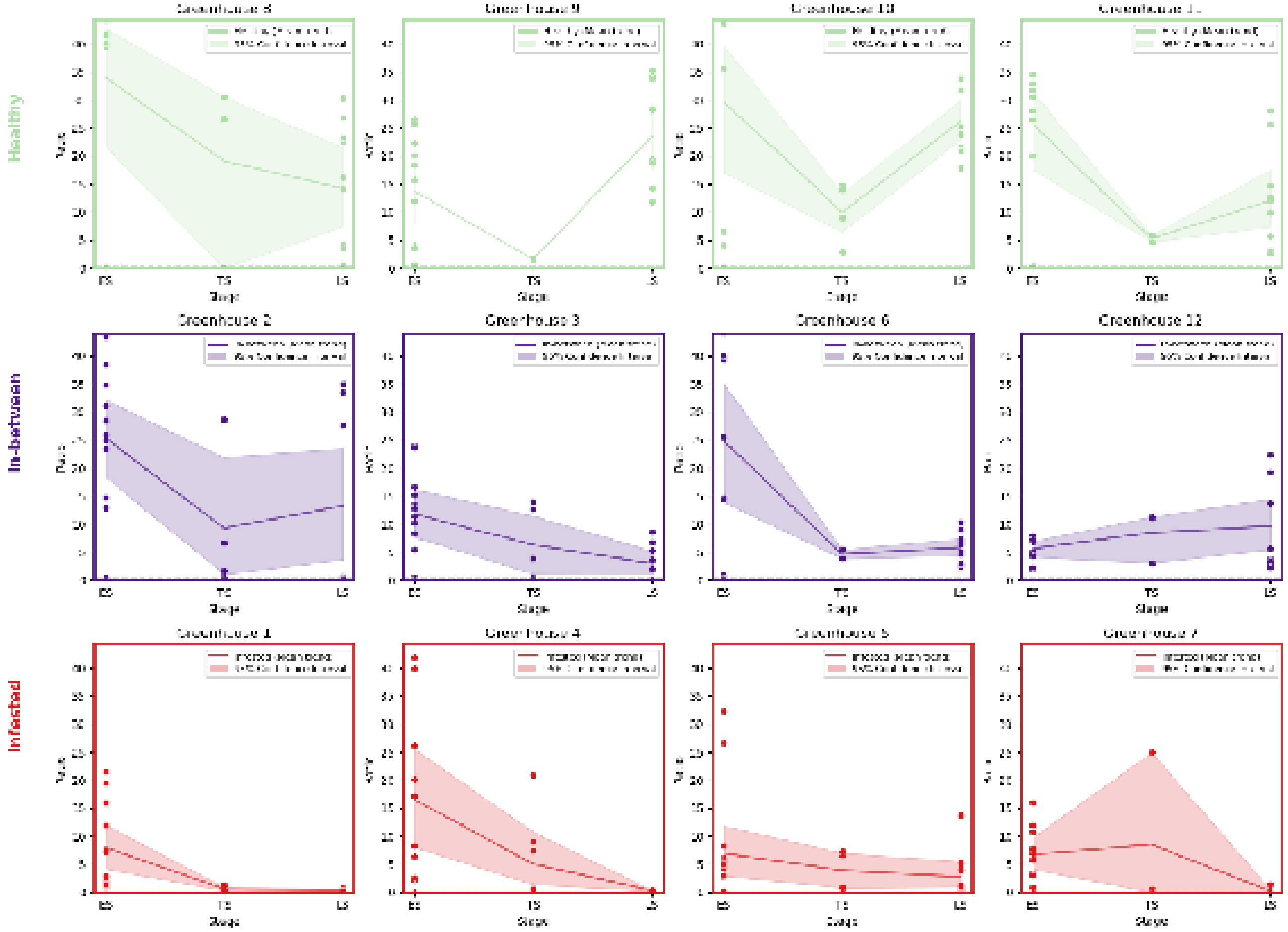

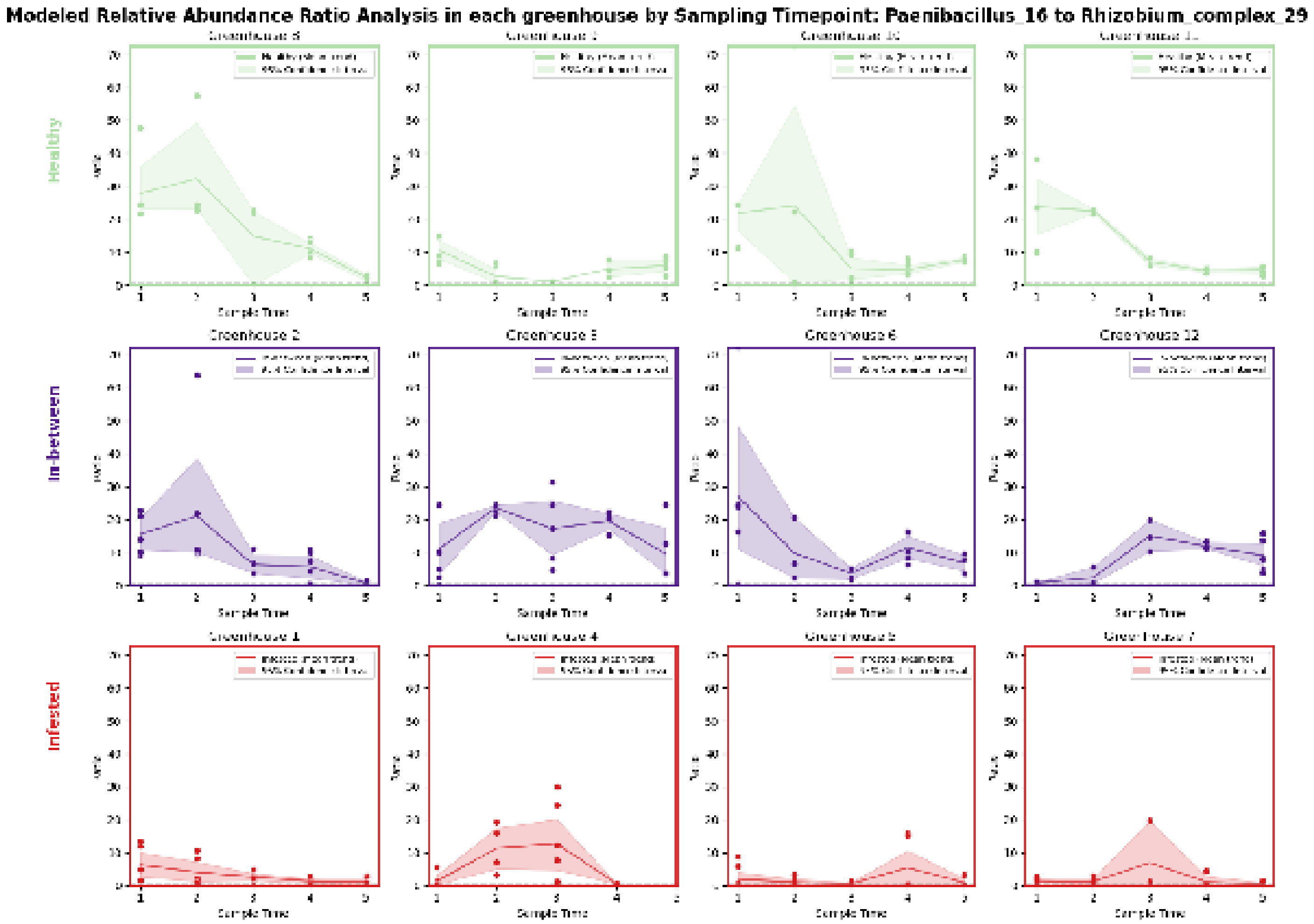
Modeled Relative Abundance Ratio Analysis in each greenhouse by Sampling Timepoint: Paenibacillus_16 to Rhizobium_complex_29
Healthy
In-between
Infested
Greenhouse 2
Greenhouse 3
Greenhouse 6
Greenhouse 12
Greenhouse 1
Greenhouse 4
Greenhouse 5
Greenhouse 7
Sample Time

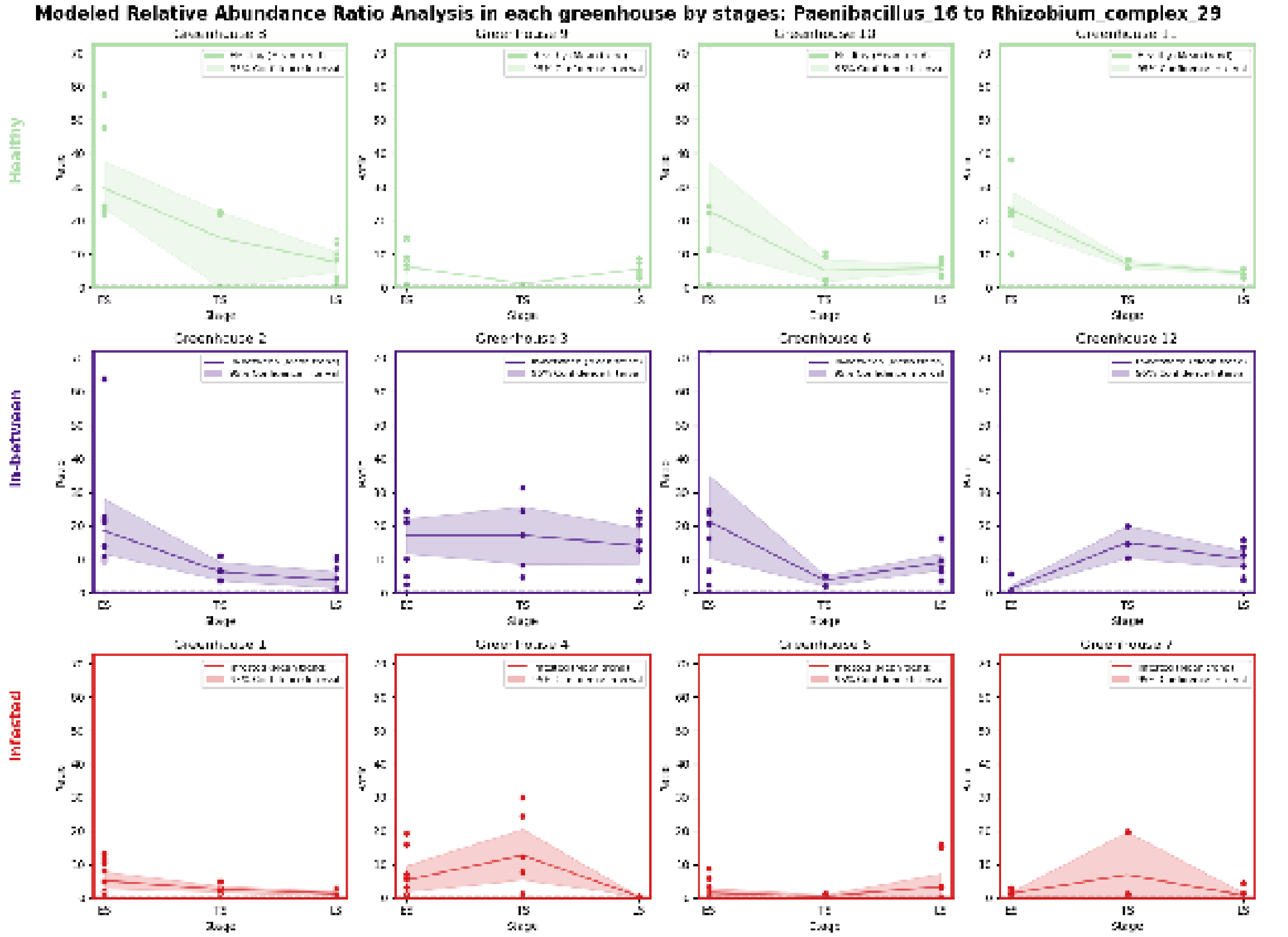
Modeled Relative Abundance Ratio Analysis in each greenhouse by stages: Paenibacillus_16 to Rhizobium_complex_29
Healthy
In-between
Infested
Greenhouse 2
Greenhouse 3
Greenhouse 6
Greenhouse 12
Greenhouse 1
Greenhouse 4
Greenhouse 5
Greenhouse 7
ES
TS
LS
Stage

Modeled Relative Abundance Ratio Analysis in each greenhouse by Sampling Timepoint: Paenibacillus_4 to Rhizobium_complex_25

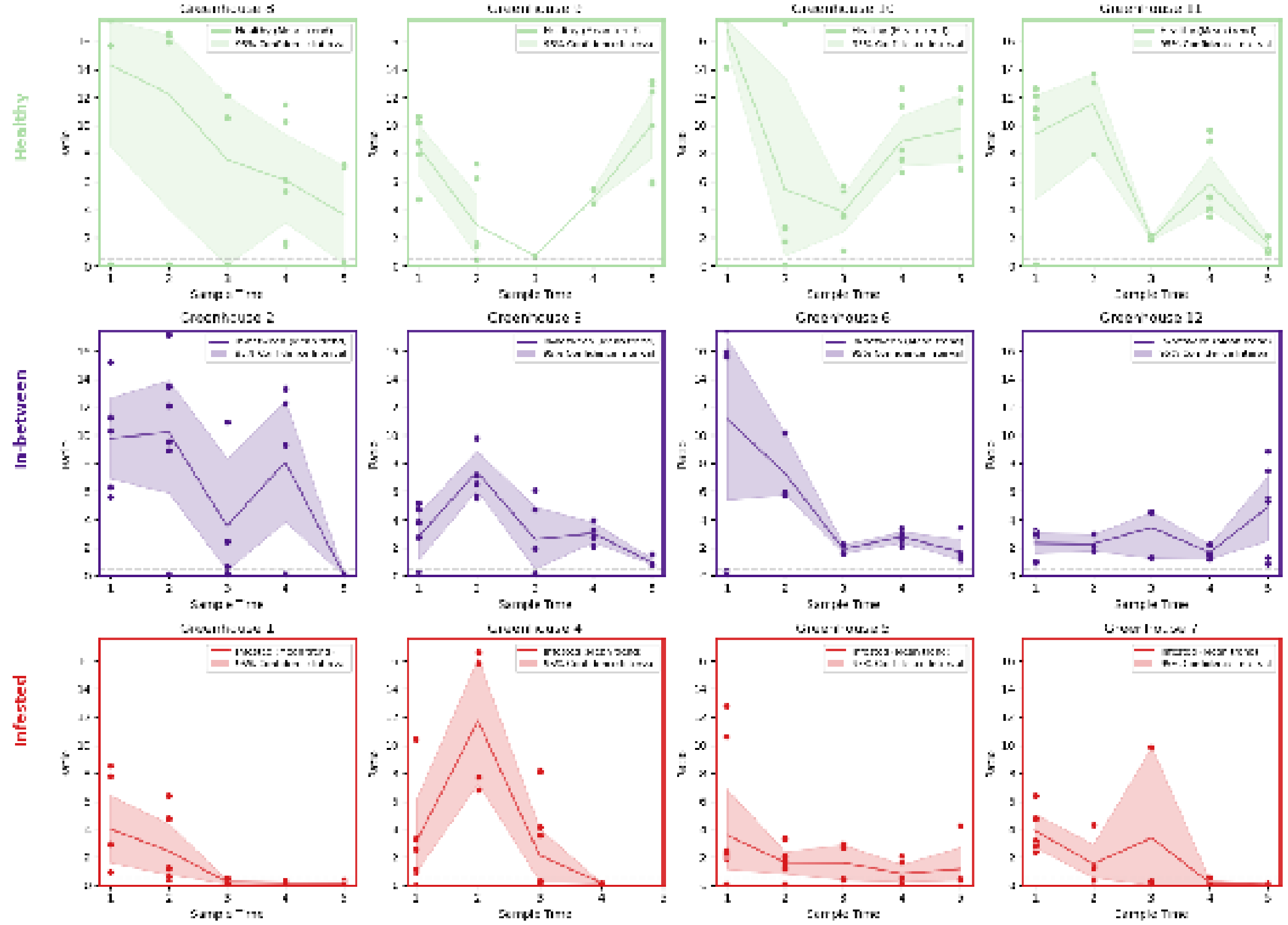

Modeled Relative Abundance Ratio Analysis in each greenhouse by stages: Paenibacillus_4 to Rhizobium_complex_25

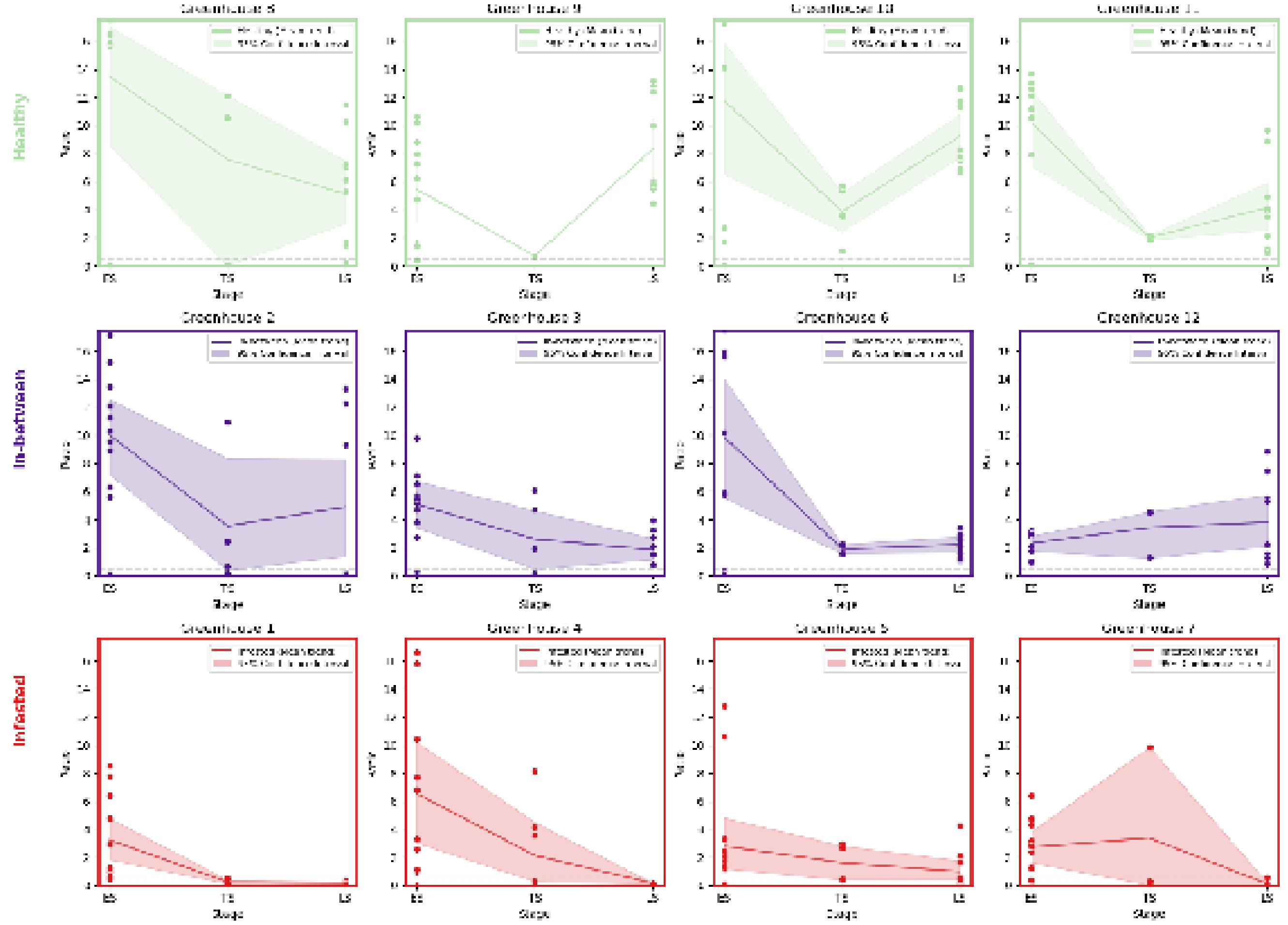

Modeled Relative Abundance Ratio Analysis in each greenhouse by Sampling Timepoint: Paenibacillus_4 to Rhizobium_complex_29

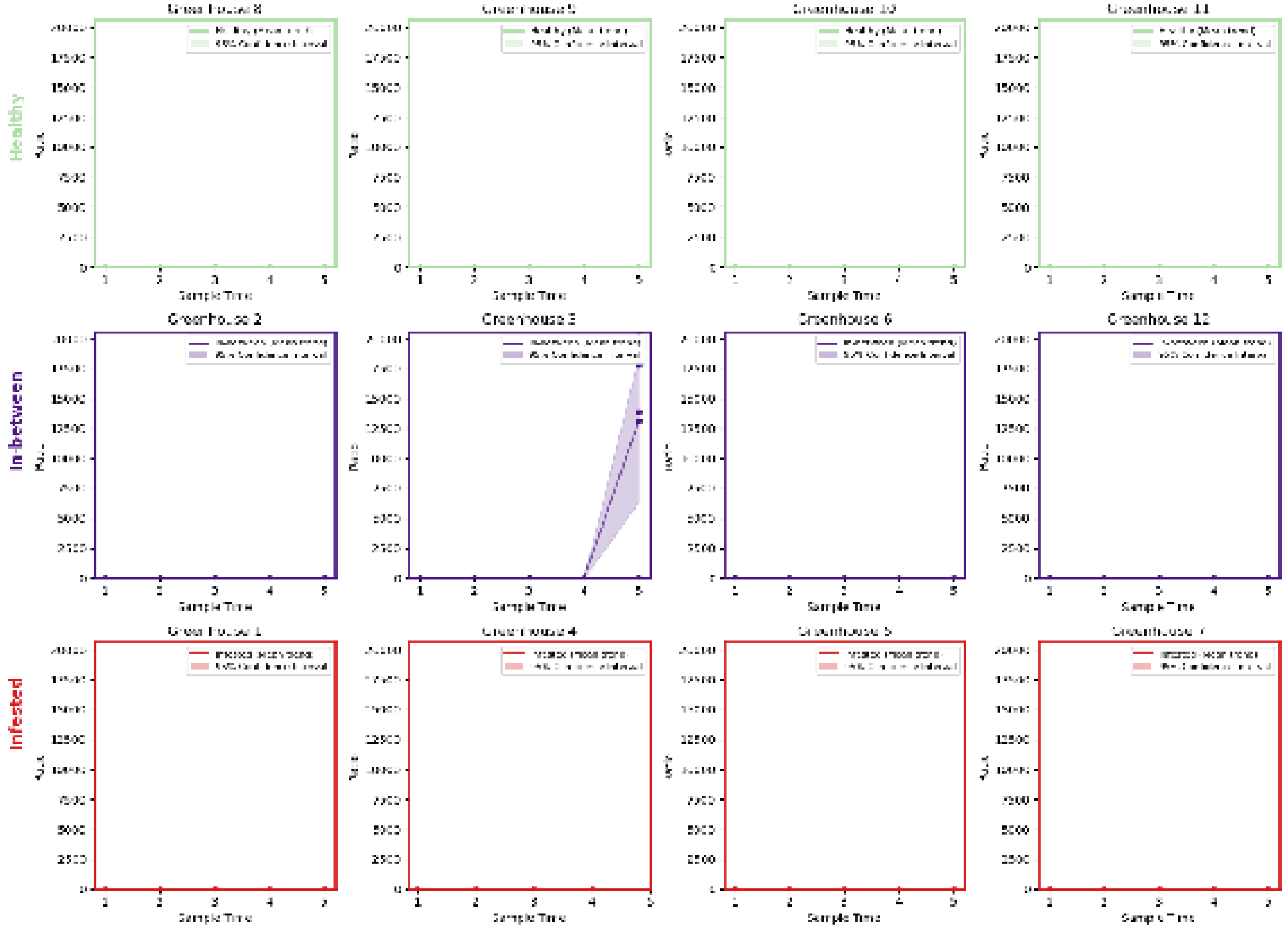

Modeled Relative Abundance Ratio Analysis in each greenhouse by stages: Paenibacillus_4 to Rhizobium_complex_29

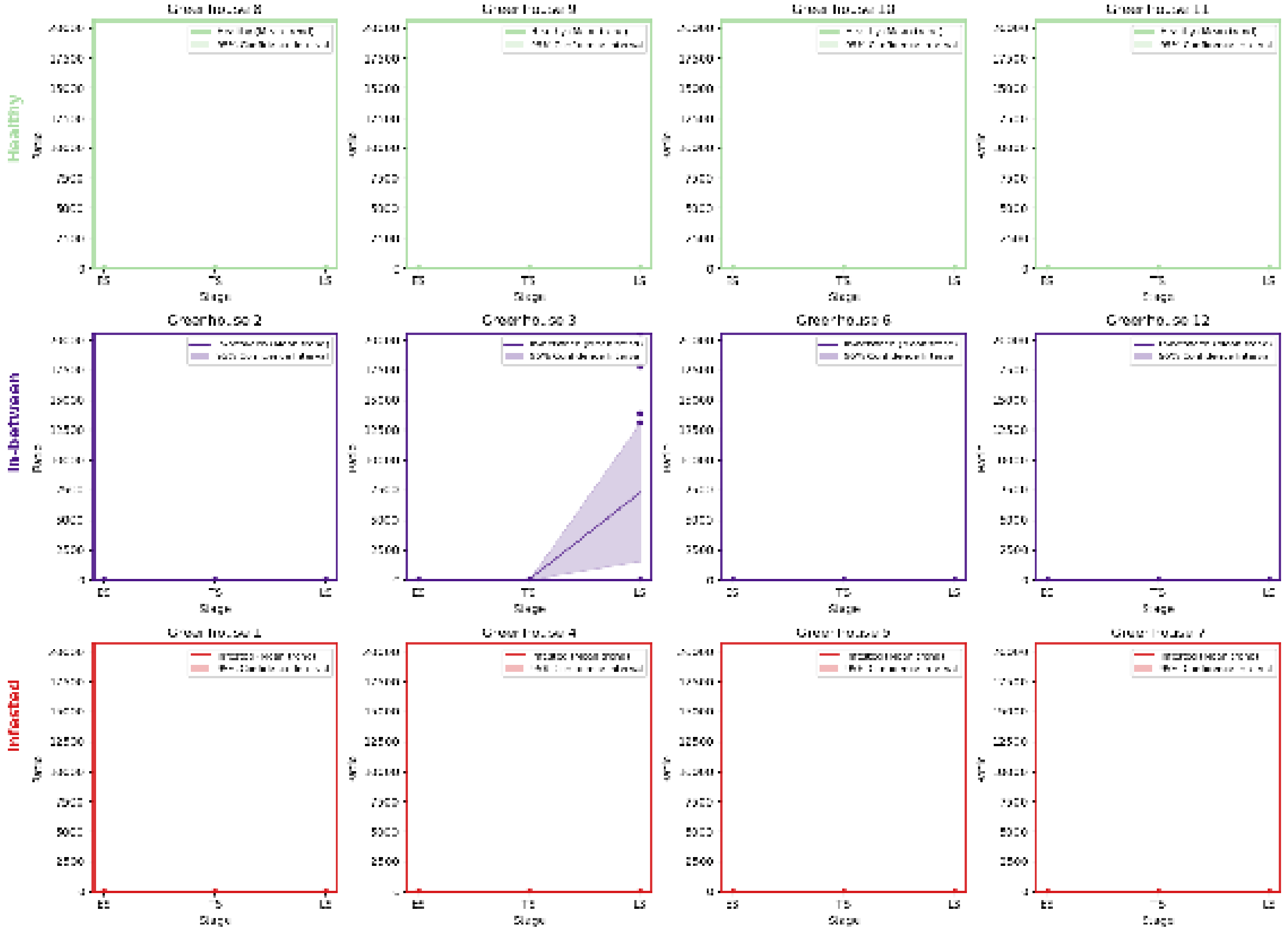

Supplement: Supplementary file 2 — Additional file 2. [file 40793_2025_822_MOESM2_ESM.pdf]
