# Supplementary material for: Latent Dirichlet Allocation reveals tomato root-associated bacterial interactions responding to hairy root disease
Source: Environ Microbiome. 2025 Nov 23;20:161. doi: 10.1186/s40793-025-00822-2 (PMC12751256; doi:10.1186/s40793-025-00822-2)
Supplement: Supplementary file 3 — Additional file 3. [file 40793_2025_822_MOESM3_ESM.docx]

Interactive heatmap visualization used during data analysis. Available at <https://marimo.io/p/@peiyang-huo/notebook-rvkcan> (For the application view, please click the three-dot menu in the top right and turn off ’show code’). Different heatmap ordering strategies are available through a dropdown menu, and brushing selection is available.

Below are static heatmaps by different ordering strategies.


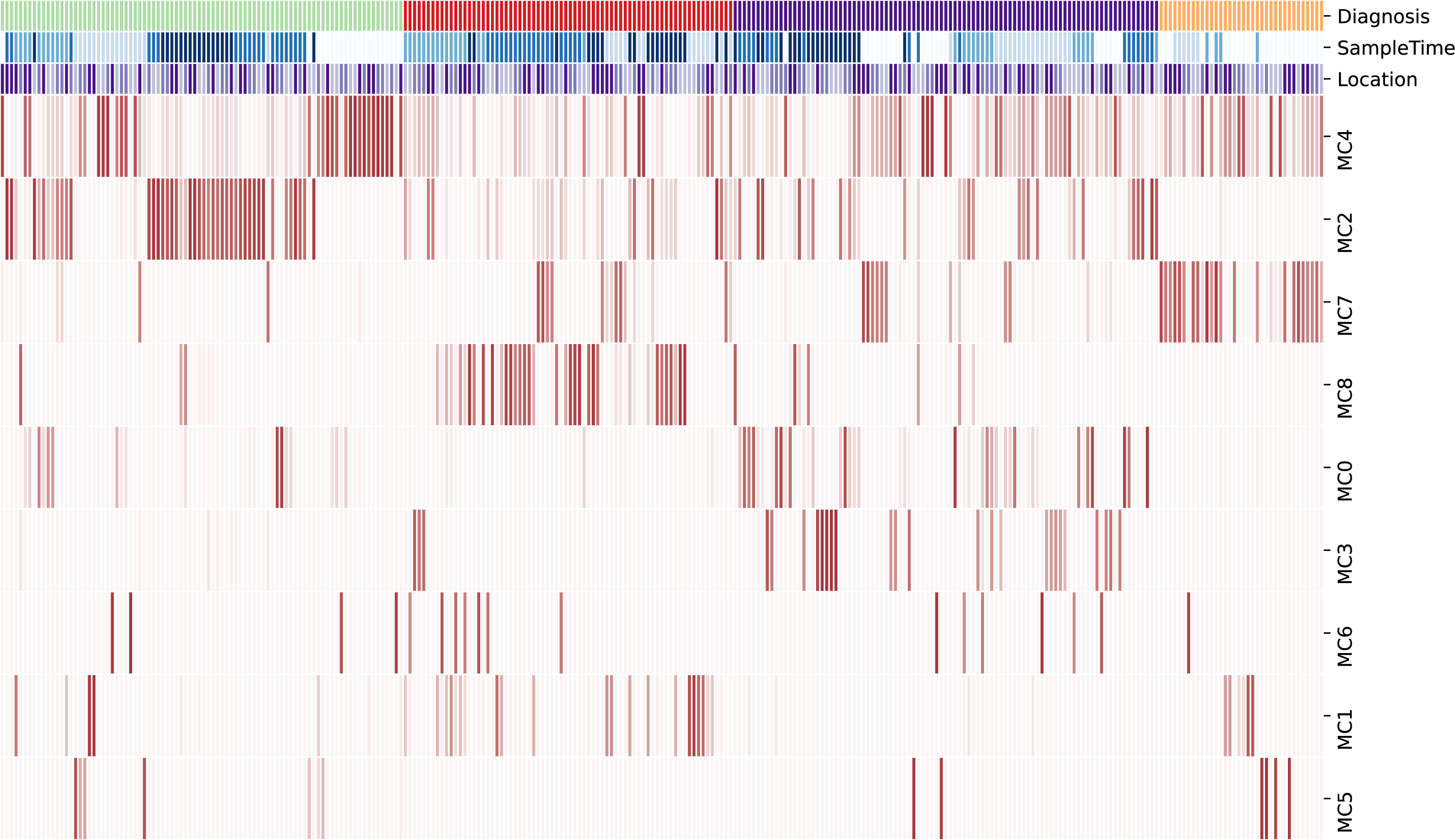


Fig. 1: Sample-MC heatmap ordered by Diagnosis. Sharing legend with Fig. 3 in the main manuscript.


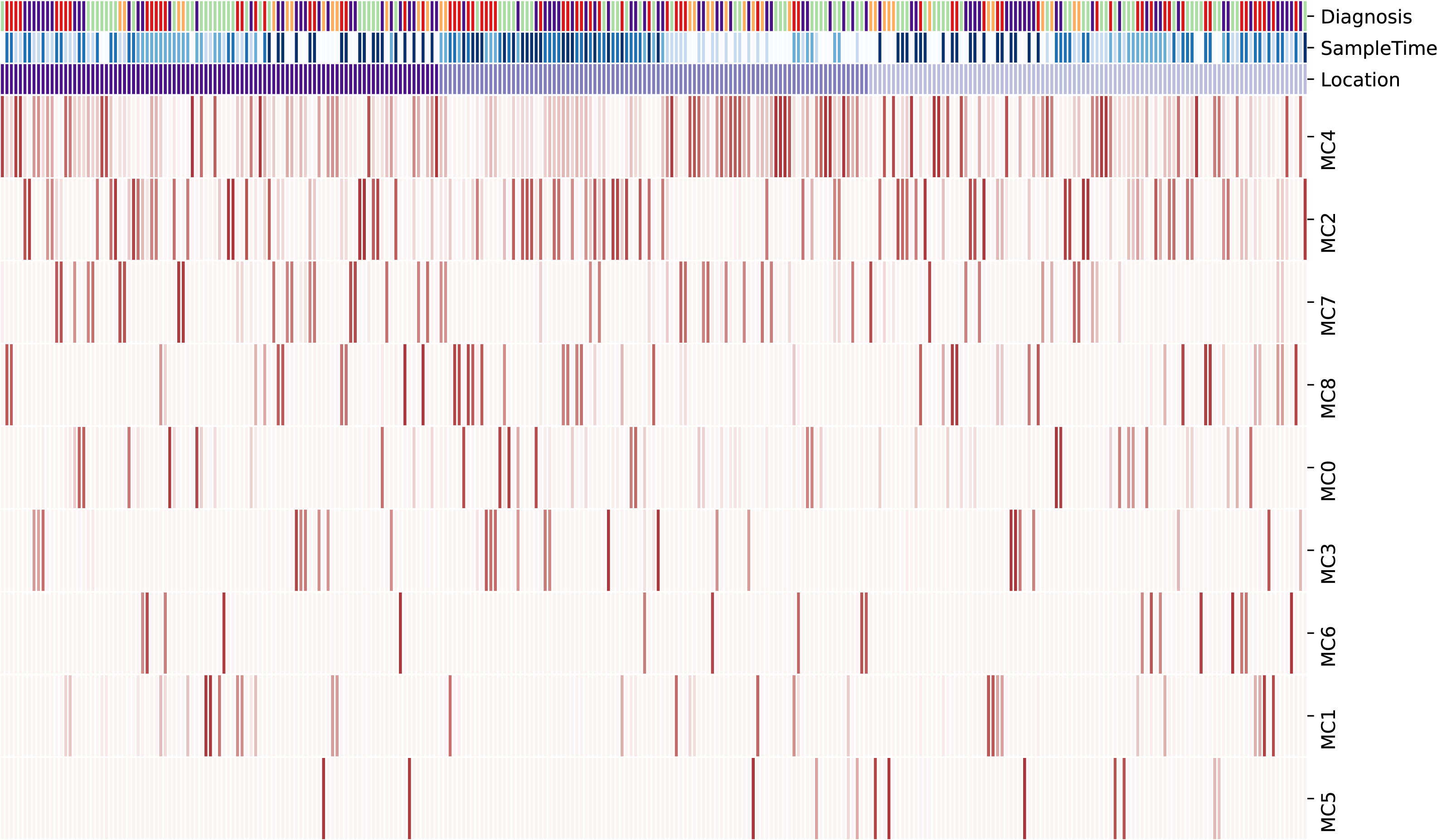


Fig. 2 : Sample-MC heatmap ordered by Location. Sharing legend with Fig. 3 in the main manuscript.


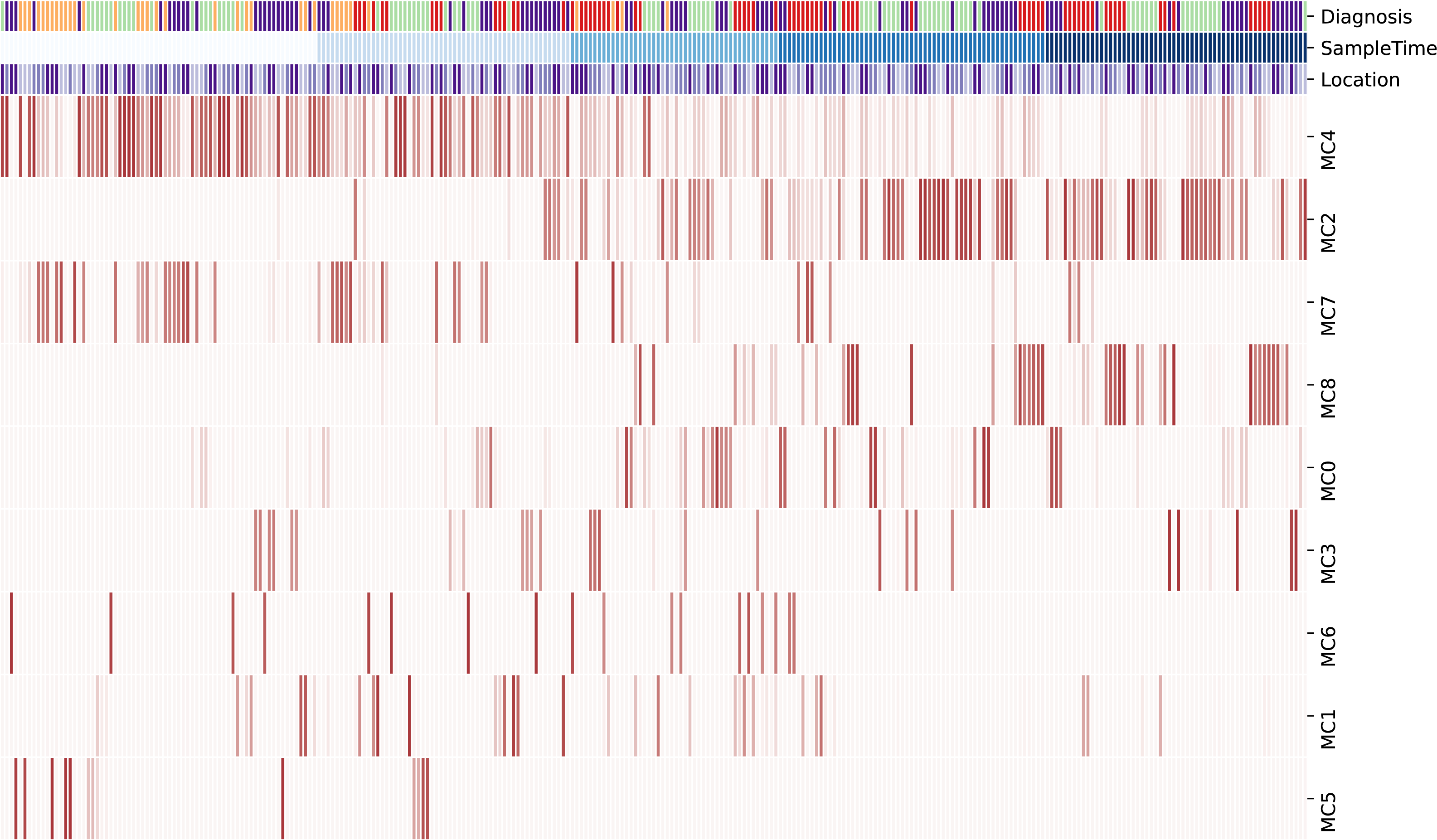


Fig. 3: Sample-MC heatmap ordered by Sample Time point. Sharing legend with Fig. 3 in the main manuscript.
